# A physics-informed eikonal model for simulating arrhythmias in the human heart in real-time

**DOI:** 10.1038/s41467-026-76050-0

**Published:** 2026-07-29

**Authors:** Thomas G. Schrotter, Matthias A. F. Gsell, Elena Zappon, Christoph M. Augustin, Aurel Neic, Simone Pezzuto, Gernot Plank

**Affiliations:** 1 Gottfried Schatz Research Center, Division of Medical Physics and Biophysics, Medical University of Graz, Neue Stiftingtalstraße 6, Graz, Austria; 2 NumeriCor GmbH, Conrad-von-Hötzendorfstraße 26/2, Graz, Austria; 3 BioTechMed-Graz, Mozartgasse 12/II, Graz, Austria; 4 Department of Mathematics, University of Trento, Via Sommarive 14, Trento, Italy; 5 Euler Institute, Universitá della Svizzera italiana, Via Buffi 13, Lugano, Switzerland

**Keywords:** Cardiovascular biology, Computational science

## Abstract

Personalized computational models of cardiac electrophysiology, referred to as cardiac digital twins, are considered a key technology for realizing precision cardiology by tailoring therapies to individual patients. However, the high computational demands of physically detailed simulations prohibit broader adoption of cardiac digital twins in practical applications. Here, we aim to address this challenge with a lightweight Physics-Informed Eikonal model that replicates the biophysical phenomena embedded within cardiac electrophysiology models along with clinical electrograms in near real-time, including arrhythmia scenarios. Validity of the proposed model is shown against a gold-standard cardiac electrophysiology model in complex arrhythmia simulations, including a scar-mediated ventricular tachycardia in a human whole-heart model. In this work, we show that the Physics-Informed Eikonal model delivers near-real time performance and retains high physical fidelity as required for the calibration of cardiac digital twins, thus providing a fundamental core technology to be leveraged in industrial and clinical applications.

## Abstract (web summary)

The authors present a model for real-time simulation of whole-heart electrophysiology and associated electrocardiograms with high physical fidelity, providing a technology for leveraging cardiac digital twins in clinical practice.

## Introduction

Computational models of cardiac electrophysiology (CEP) provide a mechanistic framework capable of precisely predicting real-world behavior of the heart. Their application scope is determined by the fidelity achieved through calibration procedures that aim to minimize the discrepancy between behaviors in real and virtual space. If the gap is small and the response of a heart to a perturbation is predictable in silico, with sufficient certainty, such models can be used to evaluate the efficacy and safety of device^1^ or drug^2^ therapies. Thus, they offer vast potential to reduce economic costs and improve patient outcomes^3^. A most advanced realization of such a predictive CEP model is the concept of a cardiac digital twin (CDT)^4^–a virtual replica of a real-world human heart that is synchronized with real-time data to provide a holistic view on a heart’s function.

However, realizing such high-fidelity CDTs remains a major challenge. First, the computational costs of numerically evaluating a mechanistically complete high-fidelity description of human CEP, such as the gold-standard cardiac bidomain model^5^, are prohibitively expensive^6^. Secondly, the process of calibrating a CDT by matching model predictions to observed data, referred to as functional twinning, constitutes a many-query application that requires a large number of model evaluations to perform parameter inference, sensitivity analysis and uncertainty quantification. Thus, realizing CDTs with current simulation technologies becomes unfeasible as the evaluation of a single patient takes days^7^ on high performance computing resources^6,8,9^ which prohibits broader adoption of CDTs.

Attempts to enhance computational efficiency focused on optimizing implementations by exploiting parallelization on Central Processing Units (CPUs)^6^, Graphics Processing Units (GPUs)^8–10^ or through spatio-temporal adaptivity^10,11^. However, none of these approaches achieved sufficient performance for leveraging feasible CDT applications due to the spatio-temporal discretization constraints imposed by reaction-diffusion (RD)-based CEP models. Relaxing constraints by coarsening the spatial resolution reduces computational costs, but yields increasingly lower fidelity solutions^6^ or even numerical artifacts that produce emergent phenomena which would not occur with adequate discretization^12^. Realizing CDTs therefore requires alternative methods that are fast to evaluate and retain the key CEP mechanisms necessary to maintain predictive capabilities. Recent developments in data-driven surrogate CEP modeling based on machine learning have shown promise, but are so far limited to simplified toy applications^13–18^. With regard to physiologically complex CDTs, evidence of feasibility is lacking and projections on applicability appear overly optimistic^19^.

An alternative approach is to recast the RD model into a traveling wave front problem that lifts the spatio-temporal discretization constraints^20^ to facilitate simulations of organ-scale CEP robustly at coarse resolutions in near real-time. Such methods employ an Eikonal-based wave propagation mechanism that can be coupled to RD models to support physiological wave front propagation at coarse spatial resolutions^21–23^, or directly recover a RD solution from a prescribed traveling wave front solution space^24^. However, the application scope of these approaches is limited as these yield solutions only for stable limit-cycle behaviors, but cannot account for dynamic history-dependence of CEP under irregular repetitive activations occurring under cardiac rhythm disturbances such as arrhythmias. Recent attempts to incorporate history-dependent physiology have been presented^23,25–27^, but limitations persist due to their incomplete physiological scope.

In this work, we present a Physics-Informed Eikonal (PIE) model that is capable of accurately reproducing the spectrum of biophysical phenomena–including arrhythmias–embedded in RD-based CEP models in near real-time, thus overcoming the limitations of state-of-the-art RD, data-driven or Eikonal-based approaches. High computational performance is achieved by enabling lightweight modeling on coarser anatomical grids, whereas physical fidelity is retained by measuring dynamic system properties in small-scale high-fidelity RD models, and encoding these in lookup maps that govern finite state transitions controlling physical behaviors. The PIE model is able to simulate CEP and associated clinically observable signals such electrocardiograms (ECGs) with high physical fidelity in near real-time, rendering like-for-like comparisons to clinical data feasible. Our model delivers the sought-after performance and fidelity required for the calibration of CDTs, providing the core technology for leveraging CDTs in industrial and clinical applications.

## Results

### Overview

**Fig. 1:**
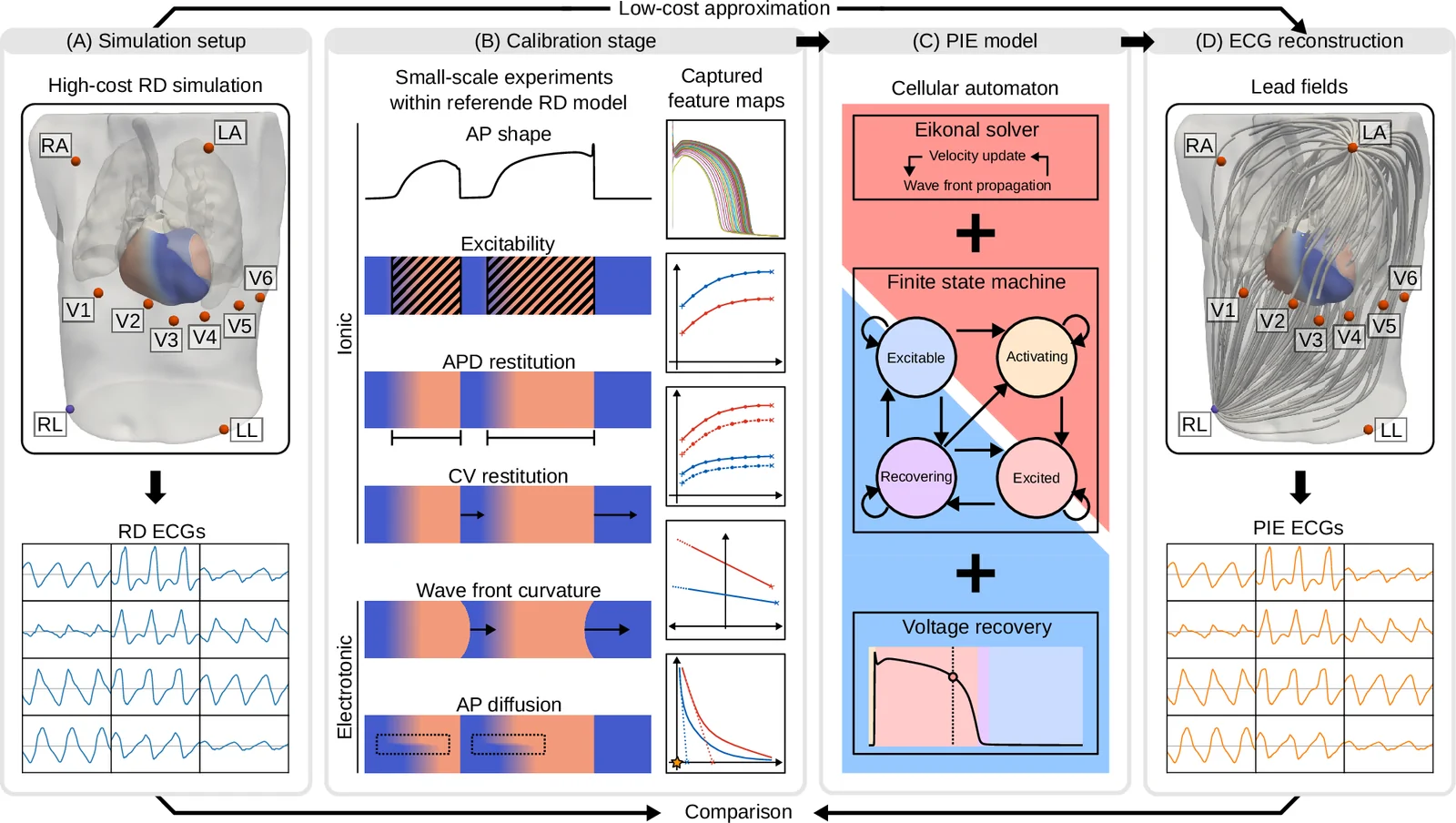
Physics-Informed Eikonal (PIE) simulation framework. **A** Example of a reference high-fidelity, high-cost RD model for simulating an infarct-mediated ventricular tachycardia (VT)and associated characteristic ECG. **B** The PIE model attains physiological fidelity during calibration from a reference RD model by measuring physiological properties efficiently in small-scale high-fidelity cell- and tissue-level experiments. For each type of excitable tissue in the simulation domain, history-dependent excitability, APD and CV restitution, as well as CV dependence on electrotonic curvature and diffusion effects are measured and encoded in feature maps. **C** The PIE model comprises an Eikonal wave propagation model coupled to a Finite State Machine (FSM) which controls AP phases according to the feature maps. Cardiac source distributions are recovered from the traveling wave solution by means of AP lookup in the measured feature map. **D** Lightweight ECG reconstruction using a lead-field approach.

The PIE framework aims to facilitate real-time cardiac electrophysiology (EP) simulation while retaining high physical fidelity comparable to the computationally expensive gold-standard RD-based bidomain model of CEP^5^. Crucially, setup and calibration of the PIE model is flexible and does not require expensive training associated with machine learning-based EP models. A high-level overview of the PIE framework is shown in Fig. 1A-D. The PIE model encodes physics by measuring dynamic system properties in small-scale high-fidelity reference RD models through a fully automated, standardized and low-cost calibration stage^28^ (Fig. 1B). The calibration procedure captures key CEP properties governing emergent physiological behaviors that dictate activation and repolarization patterns in the human heart under normal and diseased conditions, including complex irregular repetitive activation patterns as they occur during arrhythmias. Activation and repolarization patterns critically depend on the velocity and duration of propagating action potentials (APs). Their dynamics are governed by history-dependent factors referred to as cardiac restitution properties which entail a reduction in velocity, a change in shape of the AP and a shortening of action potential duration (APD) as a function of the time elapsed between the end of a previous AP and the onset of a new AP, a time period referred to as diastolic interval (DI). These effects are mediated by the intrinsic factors excitability–the ability of a cardiac cell in resting state to respond to an electrical stimulus by eliciting an AP, and refractoriness–a cellular state immediately following activation during which no further AP can be elicited until the cell recovers excitability when returning close to its resting state. In addition, all properties are modulated by diffusive electrotonic currents which act to smooth out wave backs by either shortening or prolonging the AP, and thus altering the instant of recovery from refractoriness over a spatial area of influence governed by the electrotonic space constant. The propagation of wave fronts and their conduction velocity (CV) is also influenced by diffusive currents by an altered source-sink ratio along curved wave fronts which accelerates or slows down CV for concave or convex wave fronts, respectively. Measurement of these properties and their encoding in feature maps used to steer the physiological behavior of the PIE model is illustrated in Fig. 1B.

Conceptually, the PIE model incorporates an Eikonal-based wave propagation mechanism operating over a short moving time horizon where all factors affecting propagation are dictated by a Finite State Machine (FSM) that imposes the measured history-dependent dynamics of cellular AP phase transitions and the impact upon propagation, as encoded in feature maps during calibration (Fig. 1C). The moving time horizon combined with the dynamic tracking of the tissue state facilitates repetitive activation, either driven by multiple stimuli or by reentrant wave fronts. Owing to this robust propagation mechanisms embedded in the PIE model, the strict spatio-temporal discretization constraints imposed by RD models are lifted. Thus, the PIE model is able to represent cardiac sources with high-fidelity at coarse spatial resolutions which yields, combined with lightweight lookup-based physics evaluations, large computational speedups to attain simulations with close to real-time performance. Importantly, all observable clinical signals such as the ECG can be recovered from the spatio-temporal distributions of cardiac sources efficiently with high physical fidelity (Fig. 1D) by leveraging a lead-field approach^24,29,30^ (Supplementary Sec. A.5). Thus, the PIE framework facilitates like-for-like comparison to clinical data in real-time, offering the performance needed for realizing CDTs.

### Evaluation

An established high-fidelity RD model of infarcted human ventricles served as a reference for validating the PIE model^7^ (Supplementary Sec. A.1 and A.2). Cell and tissue EP properties were set to characterize three different myocardial regions: normal ventricular tissue exhibiting healthy EP, electrically viable border-zone tissue adjacent to infarct scars with impaired EP, and, non-conductive infarct scar tissue. Within regions of healthy and border-zone tissue, cellular dynamics were represented by myocytes of epicardial phenotypes according to the Tusscher-Noble-Noble- Panfilov (TNNP) model^31^. For border-zone tissue, the peak sodium current, *I*_Na_, L-type calcium current, *I*_Cal_, and the potassium currents, *I*_Kr_ and *I*_Ks_, were reduced by 62%, 69%, 70% and 80%, respectively, resulting in a prolonged APD duration, a decreased upstroke velocity and a decreased AP amplitude. For each electrically viable tissue type, the reference RD model was calibrated for a prescribed target CV at a high spatial resolution of 250 *μ*m^28^. The calibrated reference RD model parameters are summarized in the Supplementary Sec. A.3.

For normal and border-zone tissue, EP properties of the RD model were measured according to the ForCEPSS standard^28^. The restitution of AP shapes and APD was recorded in single-cell experiments, whereas CV restitution curves, electrotonic space constants, and the curvature dependence of CV were measured in thin 3D strand or slab preparations. The calibration of the PIE model was carried out once for each considered tissue type, and were used then in all experiments.

**Fig. 2:**
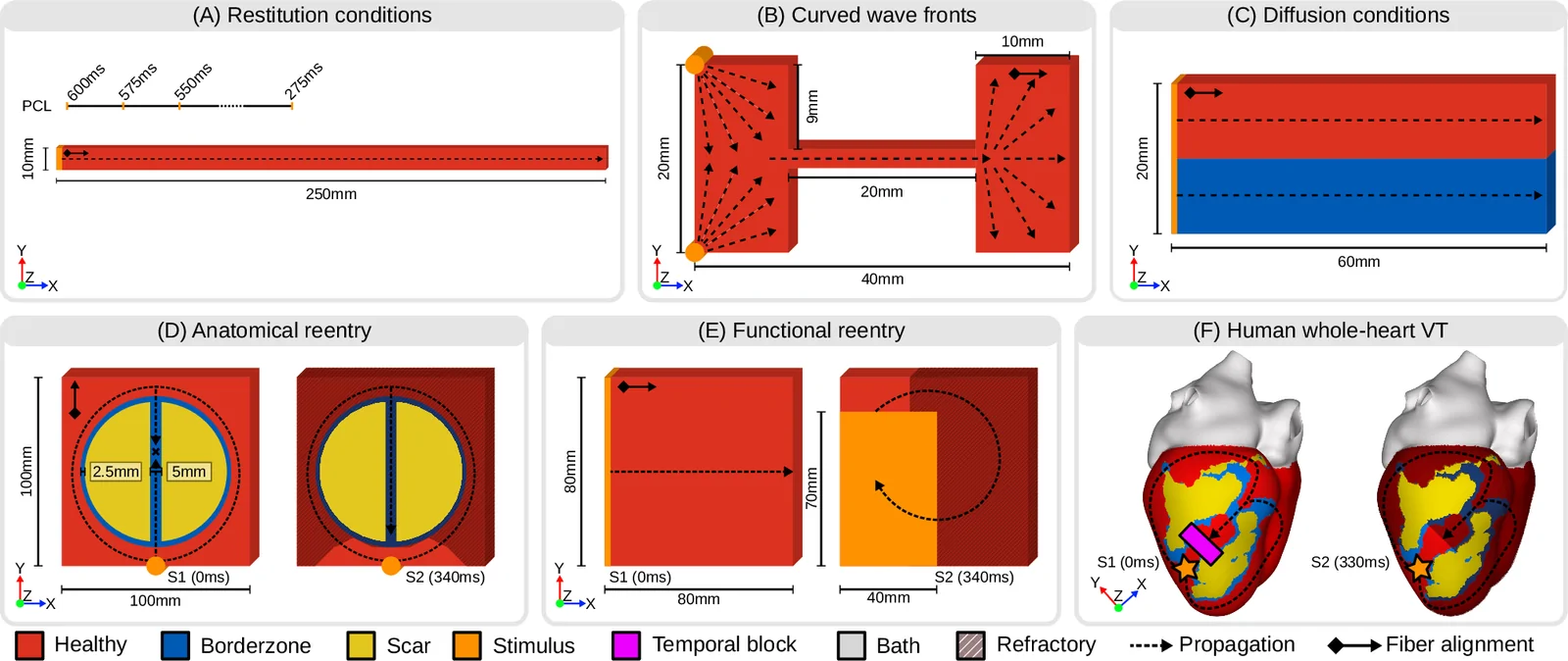
Experimental setups for evaluating physiological predictions. **A** evaluates APD and CV restitution behaviors by pacing the left face of the strip following a dynamic restitution protocol. **B** assesses wave front curvature effects by simultaneous pacing tissue on the left-hand side of an isthmus to produce concave, planar and convex wave fronts when approaching, traversing and exiting the isthmus, respectively. **C** analyzes diffusion effects across an interface between normal and border-zone tissue differing in CV and APD. Pace-induction and maintenance of arrhythmias are simulated with both RD reference and PIE model in setups of increasing complexity comprising (**D**) an infarct-mediated anatomical reentry, **E** a functional reentry in tissue slab models, and (**F**) an infarct-mediated VT in a human whole-heart model.

The ability of the PIE model to replicate the biophysical behavior captured by the calibration stage was validated in three dedicated experiments, depicted in Fig. 2A–C. These involve isolated testing of the history-dependent excitability of cardiac cells, APD and CV restitution, the CV dependence on wave front curvature, and the electrotonic diffusion effect. By comparing against reference RD model simulations, the fidelity of the calibrated PIE model was assessed based on the spatio-temporal transmembrane voltage maps, *V*_m_ (*t*, **x**), and on measures of activation and repolarization sequences. These are characterized by local activation time (LAT) maps, ${t}_{{{{\rm{a}}}}}\left({{{\mathbf{x}}}}\right)$ and CV maps, $v\left({{{\mathbf{x}}}}\right)$, as well as local repolarization time (LRT) maps, ${t}_{{{{\rm{r}}}}}\left({{{\mathbf{ x}}}}\right)$, respectively. LAT and LRT in the RD model were determined as the instants in time when *V*_m_ crosses a threshold of *V*_th_ = − 75 mV or of repolarization to 90% of the action potential amplitude with a positive or negative slope, respectively. CVs maps $v\left({{{\mathbf{x}}}}\right)$ were derived by inverting the delay vector computed as the gradient of the activation map, $\nabla {t}_{{{{\rm{a}}}}}\left({{{\mathbf{x}}}}\right)$, that is $v\left({{{\mathbf{x}}}}\right)=\parallel \nabla {t}_{{{{\rm{a}}}}}\left({{{\mathbf{x}}}}\right){\parallel }_{2}^{-1}$. The error maps were computed as signed differences between corresponding LAT, CV, and LRT maps per AP cycle.

The validity of the PIE model during reentrant scenarios was investigated by carrying out arrhythmia induction and maintenance experiments, described in Fig. 2D–F, with both the PIE and RD model. Model predictions were compared based on the spatio-temporal patterns of transmembrane voltages, *V*_m_ (**x**, *t*), the trajectories of rotor cores for functional reentry, and the ECG associated with the transmembrane voltage patterns for human whole heart simulations. In all validation experiments PIE and RD model were solved at mean spatial resolutions, *h*, of  ≈ 1000 *μ*m and  ≈ 250 *μ*m, respectively, over a 5 s simulation time window to assess long-term deviations during sustained reentrant activity between both models. All tissue models were initialized at a stable limit cycle equivalent to delivering a pacing train of 100 stimuli at a cycle length of 600 ms^28^. Stimuli to initiate propagation were delivered by clamping *V*_m_ at the stimulus location to 0 mV for a duration of 2 ms.

### Experiment A - restitution effects

**Fig. 3:**
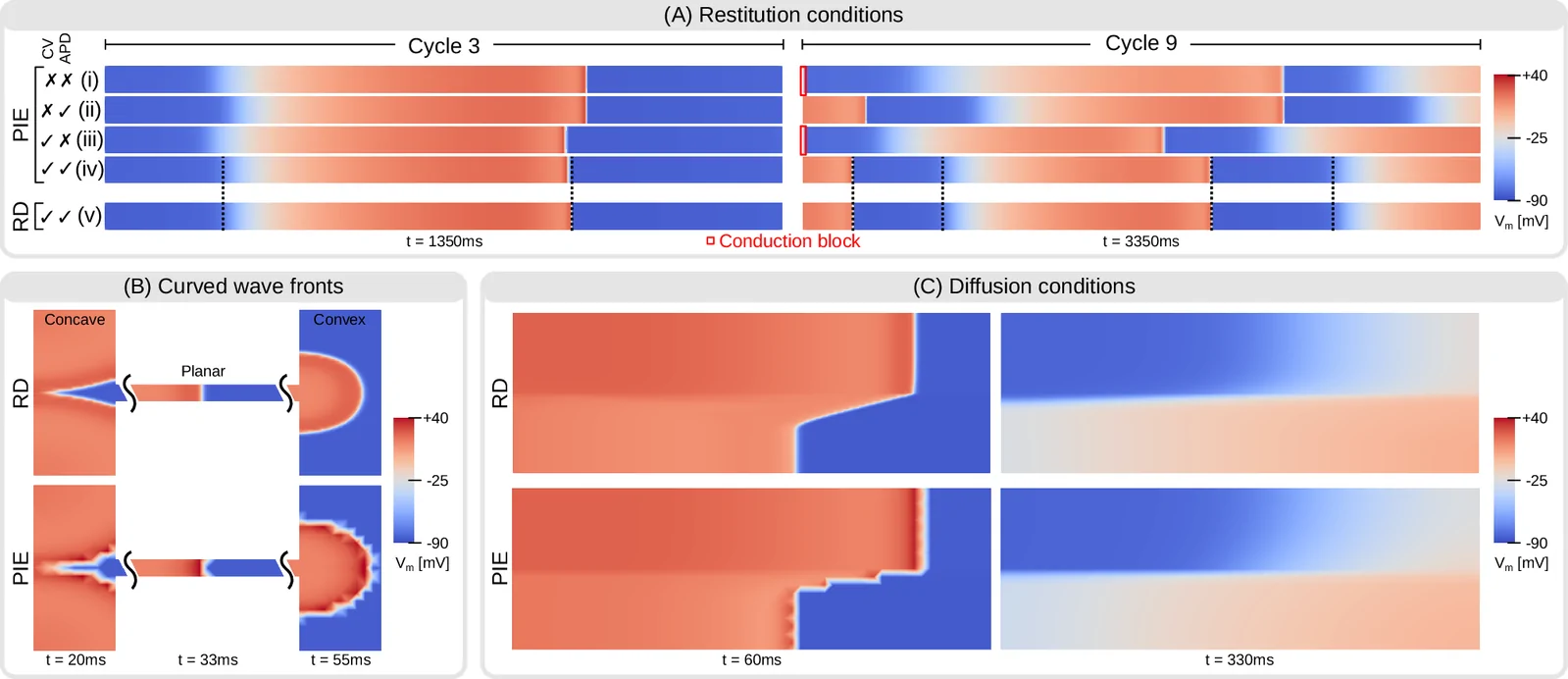
Model validation across electrophysiological restitution and wave dynamics. The ability of the PIE model to replicate electrophysiology of the RD model in terms of APD and CV restitution behavior, wave front curvature, and diffusion was evaluated. **A** Snapshots of the transmembrane voltage, *V*_m_, at longer and shorter PCLs are shown for the reference RD model relative to PIE models with four different restitution configurations: (i) no restitution, (ii) APD restitution only, (iii) CV restitution only, and (iv) with both APD and CV restitution. **B** Impact of concave (left) and convex (right) wave front curvature upon propagation. **C** Effect of diffusion upon spatio-temporally shifted wave fronts (left) and wavebacks (right) in RD (top) and PIE (bottom) model.

**Fig. 4:**
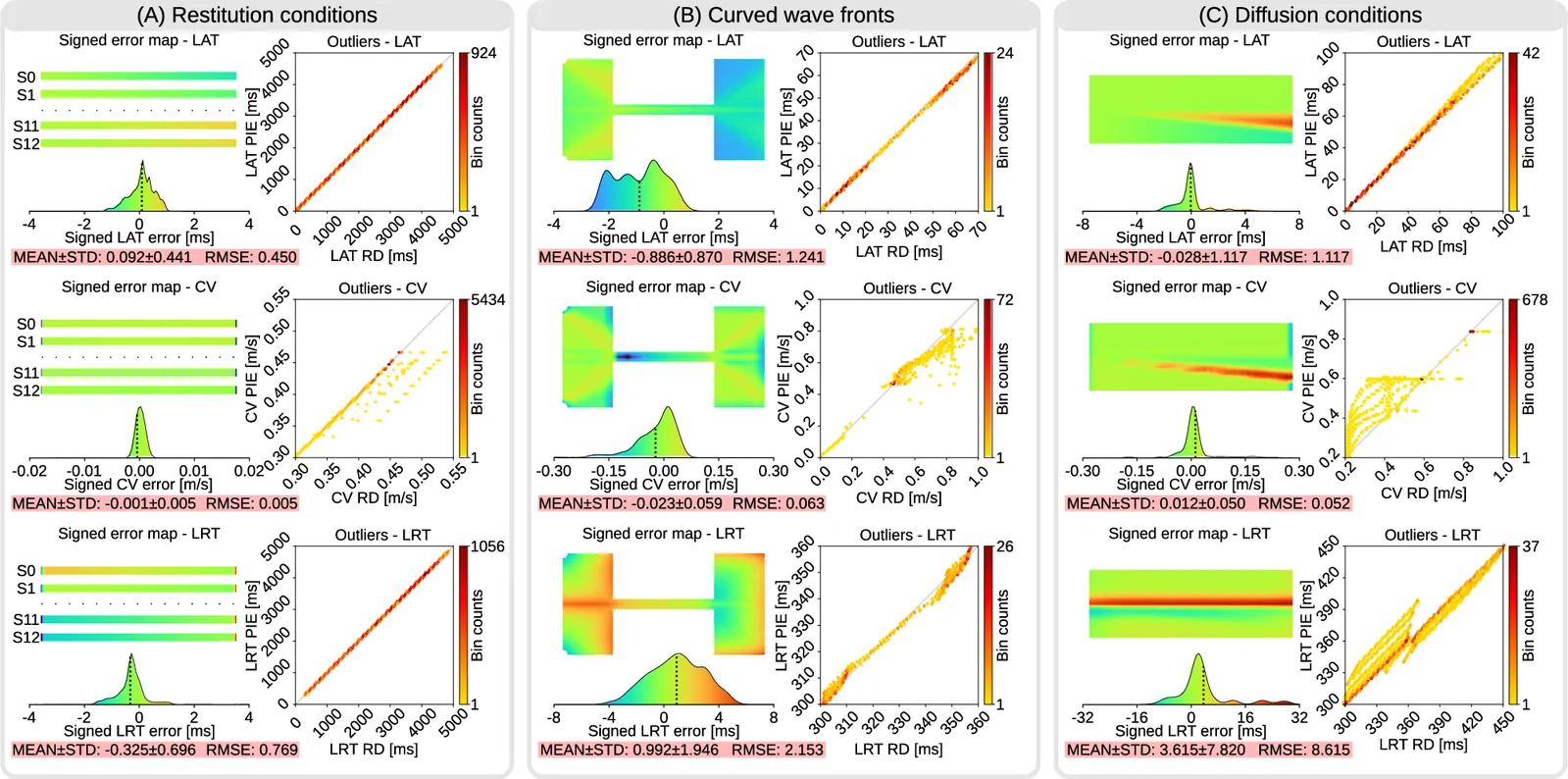
Quantitative analysis of restitution and wave dynamics experiments. Signed error maps, error density histograms and outlier plots of LATs, CVs and LRTs measured between the RD and the PIE model during (**A**) restitution conditions, (**B**) curved wavefronts and (**C**) diffusion conditions are shown. Each error map informs about the spatial error distribution for a single slice of nodal data. The corresponding error density histograms, computed from each error map, inform about error frequency across all data slices. Error maps and histograms feature shared color maps where a value smaller than zero (blue) corresponds to an underestimation, while a value larger than zero (red) corresponds to an overestimation of the PIE model. The black dotted line in each error density histogram marks the mean value over the distribution. Quantitative mean, standard deviation and RMSE values are given in red boxes for each distribution. Outliers are visualized as 2D hexagonal binning plots that compare the measurements from the RD model, aligned along the *x*-axis, against measurements from the PIE model, aligned along the *y*-axis. Hexagonal markers represent bins of nodal measurements from the PIE model that are colored based on their bin count. Bins are vertically compared against the gray identity line which represents matching values between both models. High measurement counts (red) located on or near the identity line indicate that outliers are scarce.

The ability of the PIE model to account for the dependency of the AP shape, APD, and CV upon the rate of activation was investigated using the setup in Fig. 2A. Planar wave front propagation was initiated by stimulating the left-hand face of a strip of tissue with healthy EP. A dynamic pacing protocol was used where the pacing cycle length (PCL) was gradually shortened down from 600 ms in decrements of 25 ms until loss of capture occurred after 12 cycles at a critical PCL of 250 ms. Four different configurations of the PIE model were employed: (i) without APD and CV restitution, (ii) with APD restitution only, (iii) with CV restitution only, and (iv) with both APD and CV restitution. The PIE model (iv) accounting for both APD and CV restitution was able to replicate the reference RD model, yielding virtually indiscernible solutions (Fig. 3A). Very minor errors in LAT, CV, and LRT were reported (Fig. 4A), with root mean square errors (RMSEs) of 0.450 ms, 0.005 m/s and 0.769 ms respectively. Only a sparse number of underestimated CV outliers were measured, located around the stimulation site and the sealed end where propagation terminates. All other PIE models (i)–(iii) ignoring APD or CV restitution led to major discrepancies in terms of wave front and back positions, wave length and spatial extent of the excitable gap, or to loss of capture at short PCLs ≤ 300 ms (Fig. 3A) .

### Experiment B - curvature dependency

The ability of the PIE model to replicate the dependency of CV upon wave front curvature was evaluated using the setup in Fig. 2B where wave fronts were initiated at the left-hand side corners. This forms a concave wave front when approaching the entrance into an isthmus, a planar wave front of zero curvature when traversing the isthmus, and a convex wave front of decreasing curvature when exiting the isthmus. Qualitatively, discrepancy between RD and PIE model were marginal, yielding the same wave front CV over the entire domain for both positive and negative curvature (Fig. 3B). Visual differences along the wave front are due to the coarser resolution of the PIE model, that is insufficient to smoothly resolve the steep wave front. Quantitatively, the density map in Fig. 4B showing the spatial distribution of errors reveal slightly more pronounced errors around stimulation sites, at locations of source-sink mismatch at the isthmus entrance and exit, and at sites where wave fronts collide with domain boundaries. These observations are mirrored by the outliers relating to the underestimation of high CV during the collision of concave wavefronts. LRT outliers were slightly overestimated near domain boundaries where wavefront propagation terminated, but underestimated near stimulation sites. Global RMSEs in LAT, CV and LRT were minor, with 1.241 ms, 0.063 m/s and 2.153 ms respectively.

### Experiment C - influence of diffusion

In experiment described by Fig. 2C, the effect of electrotonic diffusion across the interface between tissue domains of different electrophysiological properties was assessed. Planar wave front propagation was initiated along the interface between healthy and border-zone tissue, characterized by faster/slower CV and shorter/longer APD, respectively. Faster CV in healthy tissue leads to a dragged activation between tissue interfaces, limited in space by the transverse space constant *λ*_t_ (Fig. 3C, left panels). Similarly, diffusion in the electrotonic vicinity of the interface acts to smooth dispersion of repolarization, mediated by the combined effect of differences in wave front arrival times and intrinsic APD gradients (Fig. 3C, right panels). Quantitatively, both spatial distribution as well as global differences in terms of LAT, CV, and LRT are shown in Fig. 4C, with RMSEs of 1.117 ms, 0.052 m/s and 8.615 ms, respectively. More pronounced CV and APD outliers were witnessed, predominately emerging from the difference in asymptotic wavefront profile between the interface of healthy and border-zone tissue^32^. A detailed discussion on the sources of observed discrepancies is provided in the Supplementary Sec. C.1.

### Experiment D - anatomical reentry

**Fig. 5:**
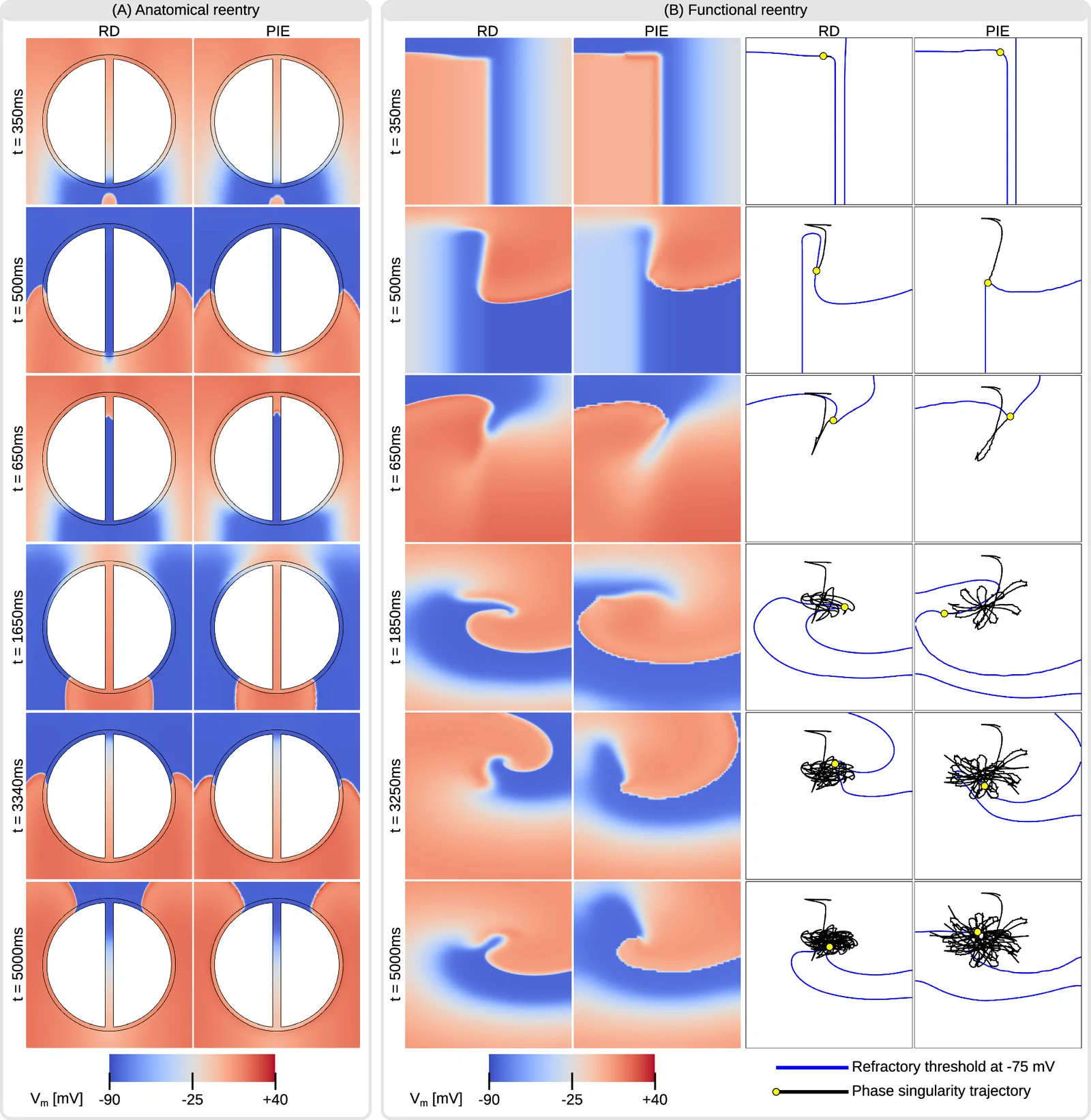
Induction and maintenance of anatomical and functional arrhythmia scenarios. In simplified 3D slabs of tissue, the spatio-temporal distribution of transmembrane voltages, *V*_m_, generated by the RD and PIE model is shown at selected snapshots in time. **A** The anatomy-mediated VT is induced by a premature S2 stimulus initiating a wave front that is blocked at the entrance into the isthmus at *t* = 350 ms to propagate along the outer loop around the scar at *t* = 500 ms, and reenters into the opposite entrance of the by now recovered isthmus at the at *t* = 650 ms. Wave fronts and wave backs move synchronously between the models over the entire simulated VT episode. **B** Functional reentrant activity is initiated in RD and PIE models. Owing to the sensitive non-linear behavior discrepancies in wave front and wave back dynamics emerge over time, but wave fronts remain spatio-temporally correlated and the phase singularity driving the spiral wave follow comparable trajectories covering the same areas.

The capability of the PIE model for capturing arrhythmia induction mechanisms in response to an S1-S2 arrhythmia induction protocol was investigated in a geometrically simplified 3D slab model of a ventricular wall with an anatomical substrate assumed to underlie monomorphic ventricular tachycardia (VT) in the human heart (Fig. 2D). The substrate is composed of a non-excitable infarct scar surrounded by border-zone tissue that is traversed by an electrically viable isthmus. The repetitive stimulation with a pulse train delivered at a location close to the entrance into the isthmus followed by a premature S2 stimulus delivered at 340 ms led to a unidirectional conduction block at the border-zone tissue which exhibited a longer APD of  ~ 360 ms. The S2 wave fronts traversed the outer loop around the scar at a slower CV due to CV restitution, providing sufficient time for the tissue at the opposite entrance to recover excitability into the isthmus. As the VT path length comprising outer loop and isthmus was long enough, an anatomical reentrant circuit was established that was maintained for the entire simulated period of 5 s (Fig. 5A).

The PIE model correctly recapitulated both induction and maintenance mechanism as observed in the RD model. Discrepancies in terms of transmembrane voltages *V*_*m*_(*t*, **x**) were marginal, with only modest differences in wave front and wave back dynamics. After 5 s of simulated VT activity wave fronts and wave back were located at virtually identical locations (Fig. 5A, bottom), indicating that the PIE model accurately represented the EP behavior of the high-fidelity RD model.

### Experiment E - functional reentry

**Fig. 6:**
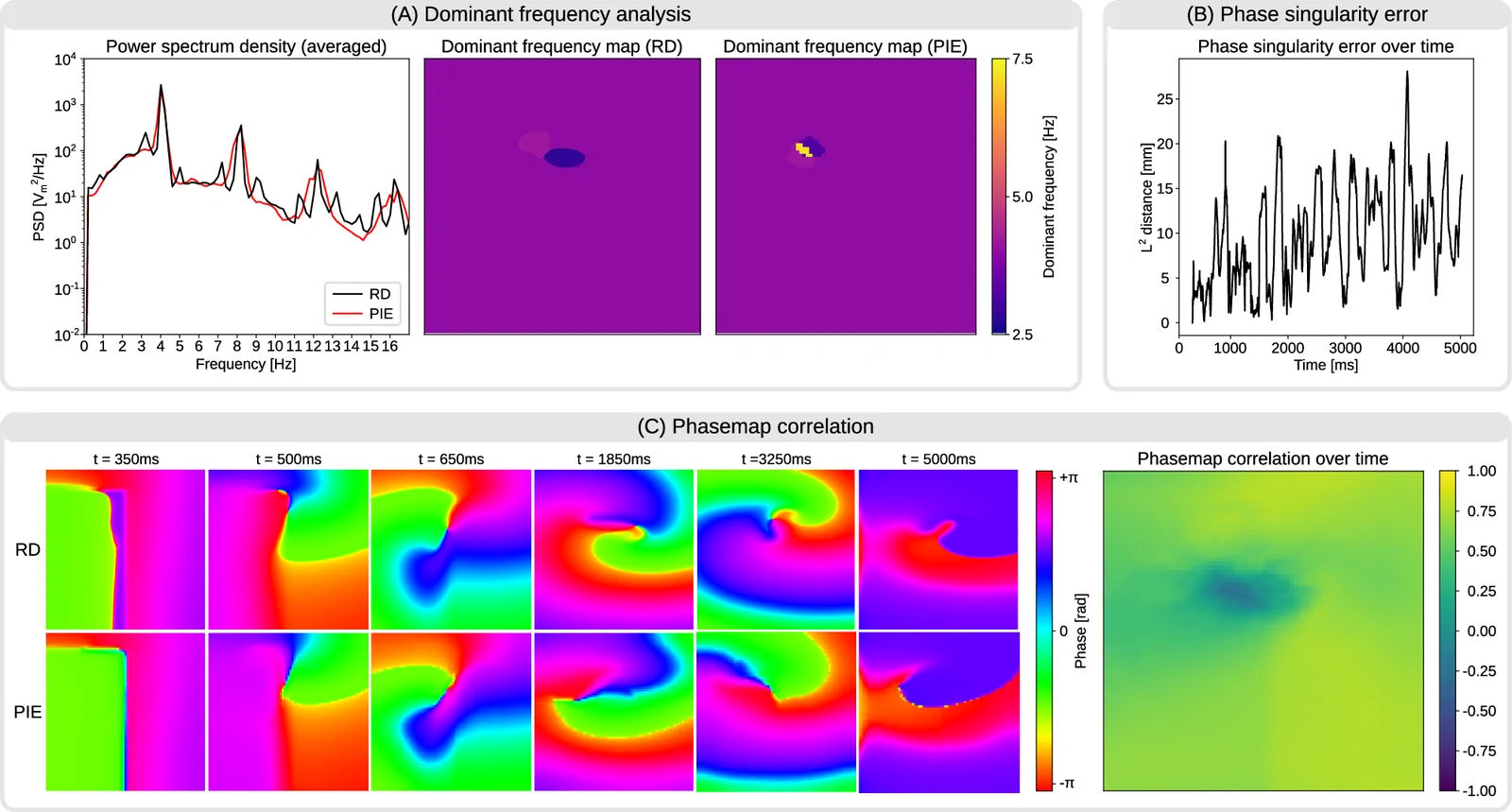
Quantitative analysis of the functional reentry experiment. **A** The PSD was computed for each nodal *V*_m_ trace to show the average PSD across all nodes over time (left) and corresponding dominant frequency maps in the RD (center) and the PIE model (right). The dominant frequency was selected as the frequency with the highest power peak in each nodal PSD. **B** The difference in phase singularity location between the RD and the PIE model was assessed based on the Euclidean distance error over the 5 s simulation window. **C** Phase maps between the RD and the PIE model were computed based on the Hilbert transform of *V*_m_^60^, ranging between [ − *π*, *π*]. The phase correlation map contains PCCs computed from each nodal phase trace and visualizes correlation between  − 1 (anti correlation) and 1 (correlation).

The ability to induce a functional reentry by applying a S1-S2 protocol was investigated in a 3D slab model of healthy tissue representing a portion of ventricular myocardium (Fig. 2E). In both RD and PIE model the S2 stimulus delivered prematurely at *t* = 340 ms led to an intersection of the critical stimulus strength isoline with the critical refractory isoline of the preceding S1 cycle’s wave back. This created a filament–a 3D line of phase singularities–around which a functional reentry in the form of a 3D scroll wave started to evolve with clockwise chirality. Agreement between RD and PIE model is analyzed qualitatively in Fig. 5B by visually comparing snapshots of transmembrane voltage maps *V*_m_ (*t*, **x**), as well as by tracking the motion of the filament at the surface over the whole simulated period of 5 s. For a quantitative comparison, the dominant frequency maps based on the power spectral density (PSD), the Euclidean distance in phase singularity location and the phase map correlation based on the Pearson correlation coefficient (PCC) were computed between both models and reported in Fig. 6.

The qualitative behavior of the PIE and RD model (Fig. 5B, left) was comparable, with wave fronts and wave backs remaining spatio-temporally correlated as they traversed the domain. The filament tracked at the surface followed comparable trajectories, covering a similar area and exhibiting a morphology consistent with previous reports^33^. The frequency analysis shown in Fig. 6A indicates that the PIE and RD models exhibit closely correlated dominant frequencies. However, the PIE model does not fully capture all frequency components of the RD reentrant activity, resulting in more irregular dynamics compared to the RD model. Frequency-related differences in reentrant behavior are also evident in the dominant frequency maps, where regions of elevated frequency are similarly localized in both PIE and RD simulations, yet differ markedly in their spatial distribution and amplitude.

The rotor in the PIE model appears less stable compared to the RD model (Fig. 5B, right), consistent with the temporal evolution of the phase singularity location error between the two models (Fig. 6B). The phase evolution of the reentrant activity (Fig. 6C) is qualitatively similar between both models. However, the PIE solution exhibits slightly reduced smoothness in phase definition near the wavefront and waveback. These differences are further reflected in the temporal evolution of phase correlation, which quantifies divergence between the two solutions over time. Phase map correlation analysis indicates that agreement is lowest in the vicinity of the rotor core, while remaining comparable in regions distal to the core.

Owing to the high sensitivity of non-linear functional reentrant behavior to initial conditions and parameter perturbations, identical dynamical behavior between two models that represent physics in fundamentally different form and are discretized with vastly different spatial resolutions cannot be achieved. This is not even the case for the high fidelity RD model where similar discrepancies emerge under minor parameter perturbations. This scenario was demonstrated in Supplementary Sec. C.2 by applying perturbed initial conditions for the RD and the PIE model during this experiment. Considering the similarity over a long observation period involving 19 activation cycles, the PIE model captures the reentrant dynamics of the RD model at a level of fidelity sufficient for most applications, at a small fraction of the computational costs (1.45%).

### Experiment F - whole-heart VT

The accuracy of the PIE model in predicting arrhythmic patterns and associated clinical signals, as required for generating CDTs, was investigated. In a most comprehensive experiment (Fig. 2F), an infarct-mediated VT with corresponding clinical 12-lead ECG was simulated in an image-based anatomically accurate human whole-heart torso model that incorporated a structurally accurate infarct scar of isthmus and outer loop topology. Monomorphic VTs in such substrates produce characteristic signatures in the ECG that are used clinically for identifying targets for ablation therapy^34^.

**Fig. 7:**
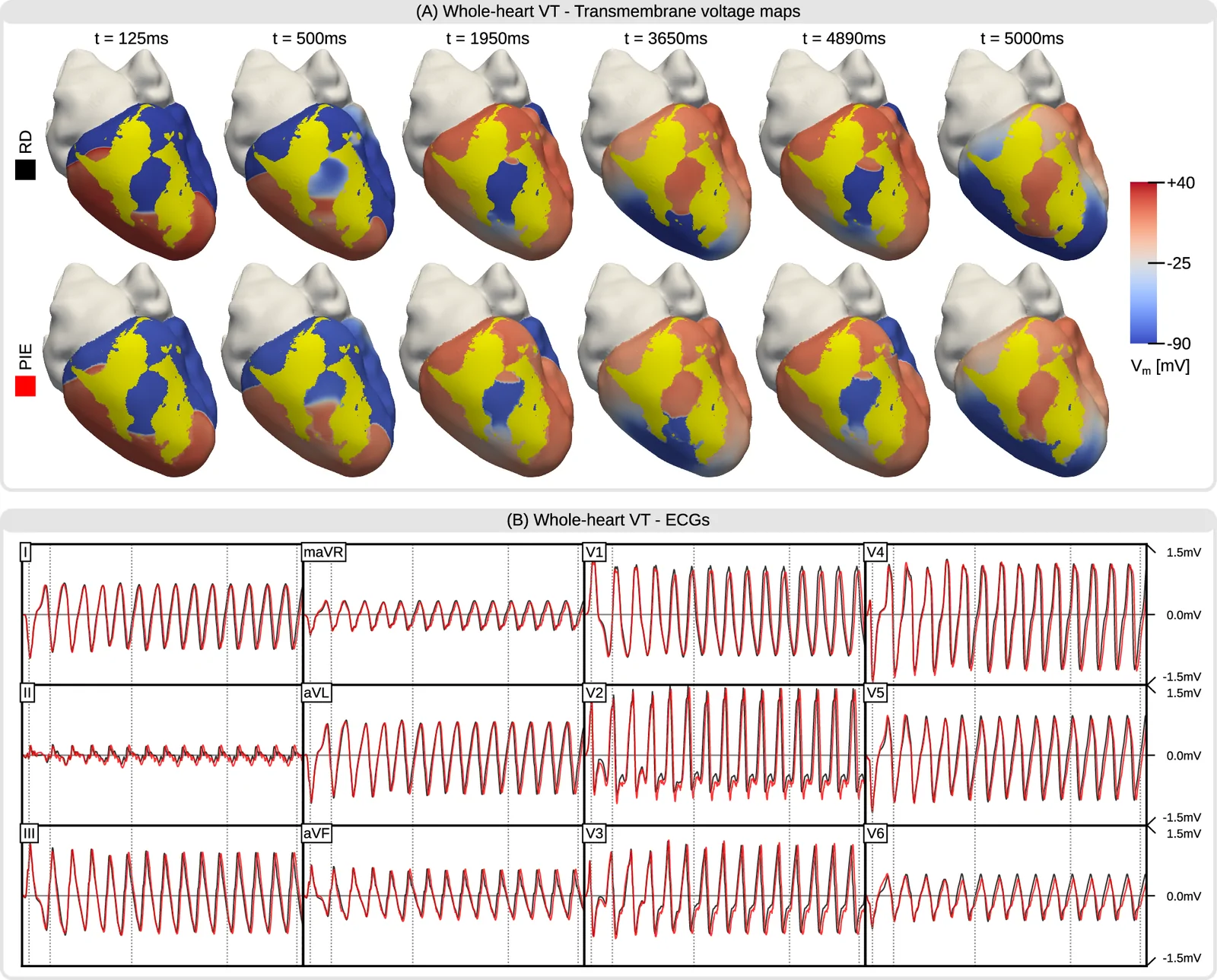
Whole-heart electrophysiology and ECG. A comparison of activation pattern and associated 12-lead ECG between RD and PIE model during an induced VT in an anatomically accurate human whole-heart torso model. **A** Snapshots taken at times *t*_*i*_ of the distribution of the transmembrane voltage, *V*_*m*_(***x***, *t*_*i*_), during 5 s of VT activity. **B** Clinical 12-lead ECG associated with VT pattern. Dotted lines refer to snapshots in time *t*_*i*_ shown in *V*_*m*_(***x***, *t*_*i*_).

In both PIE and RD models, a VT was initiated by delivering a depolarizing stimulus at the apical exit of the isthmus adjacent to a hyperpolarizing stimulus that imposed a transient block to impede propagation into the isthmus. The emerging dynamics were qualitatively assessed based on the spatio-temporal evolution of *V*_m_ (*t*, **x**) by comparing the position of wave front and wave back at various snapshots in time (Fig. 7A). Discrepancies in the predicted clinical ECGs were assessed visually in Fig. 7B and quantitatively in Table 1. Morphological similarity and correlation of predicted ECGs were assessed based on the dynamic time warping (DTW) distance measure^35,36^ and the PCC^37,38^, respectively. Long term temporal coherence between both models was assessed by comparing R-R intervals in each lead.

**Table 1:** Whole-heart ECG evaluation metrics

Metric	*Δx*[*μ*m]	I	II	III	maVR	aVL	aVF	V1	V2	V3	V4	V5	V6	MEAN ± STD
DTW [mV]	1000	0.608	1.556	0.601	0.797	**0.402**	0.772	1.566	**3.238**	0.937	0.876	1.474	1.679	1.209 ± 0.741
PCC [-]	1000	0.925	**0.707**	0.911	0.925	0.919	0.893	0.899	0.839	0.846	0.863	0.917	**0.932**	0.881 ± 0.061
R-R [ms]	250	327	333	333	327	327	333	333	332	331	328	327	327	330 ± 2
	1000	326	335	335	327	326	335	337	334	334	326	327	328	331 ± 4

A quantitative comparison between ECG lead signals computed by the reference RD, and the PIE, model based on the non-normalized DTW, distance measure^37,38^, the PCC,^35,36^ and measured R-R intervals during the whole-heart experiment (F). Lower DTW distance measures indicate a close match in morphology between signals, while the PCC describes the correlation of signals between  − 1 (anti correlation) and 1 (correlation). Bold entries mark the best and worst DTW and PCC results per row. The R-R intervals were measured as the interval between consecutive R-peaks, from which mean and standard deviation were computed across all leads.

Visual discrepancies in the spatio-temporal distribution of *V*_m_ (*t*, **x**) were negligible between PIE and RD model. A close correspondence in time of arrival of wave front and wave back was maintained over the entire 5 *s* of simulated activity, indicating that all physiological features affecting CV and APD and, thus, the wave length and excitable gap corresponding to the AP and DI in space, are captured by the PIE model with high-fidelity.

The agreement between models depended on calibration choices, such as the protocol used to measure restitution properties, as well as on numerical parameters such as mesh resolution and coarsening strategy. Because the latter factors influence both model fidelity and computational performance, we conducted a comprehensive quantitative analysis (Supplementary Sec. C.3).

The VT signature in the ECG corresponded very closely between both models. Differences were minor overall, with slightly more pronounced morphological and temporal deviations in limb lead II and precordial lead V2 (Fig. 7B). These discrepancies are mirrored by the quantitative DTW and PCC measures reported for those leads in Table 1. Across all leads, the reported average DTW and PCC measures of 1.209 mV and 0.881, respectively, indicate high similarity between the ECGs. The average R-R interval across all ECG leads of the PIE model (331 ± 4 ms) resides within the variance of those witnessed for the RD model (330 ± 2 ms), confirming high temporal coherence between both models over the 5 s simulation window.

### Performance benchmarks

**Fig. 8:**
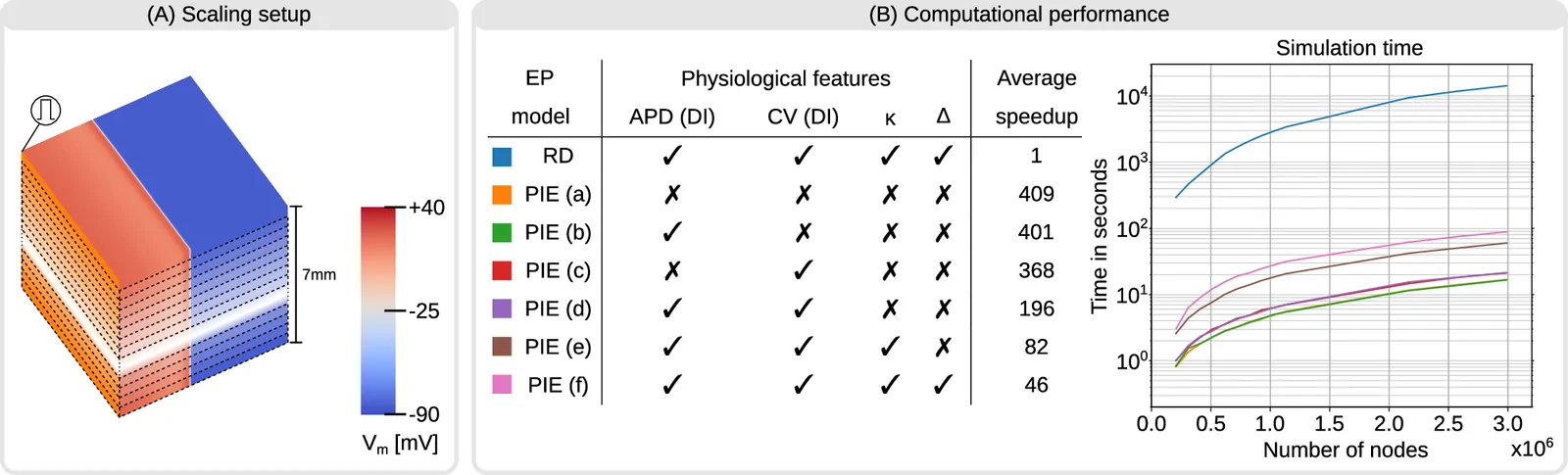
Computational performance and scaling benchmark. The computational performance of PIE models with varying physiological fidelity is compared to the reference RD model. **A** S1 stimuli were delivered to the left-hand face of a tissue slab to initiate planar wave front propagation. The transmural depth of the slab was increased from 250 *μ*m to 7000 *μ*m to increase the problem size. **B** Run time was measured for the RD model (blue) and for six configurations of increasing physical fidelity of the PIE model accounting for effects of APD restitution APD(DI), CV restitution CV(DI), curvature, *κ* and diffusion, *Δ*.

For both the PIE and RD model, computational performance and CPU time scaling as a function of mesh size were investigated based on the slab of tissue used in the functional reentry experiment (Fig. 2E) and the whole-heart experiment (Fig. 2F). The performance evaluation was conducted on either a workstation employing 32 CPU cores or on a high performance computing (HPC) cluster employing 192 cores, with detailed specifications provided in Supplementary Sec. C.4. Computational load was scaled up by increasing the transmural depth, from 250 *μ*m up to 7000 *μ*m (Fig. 8A). Compatibility with the RD model was preserved by keeping the mesh resolution constant at 250 *μ*m. The complexity of the PIE model was increased by incrementing the number of physiological features that are taken into account. This led to six distinct configurations of increasing physical fidelity that (a) ignore physiological adaptations, or consider (b) APD restitution, (c) CV restitution, (d) APD and CV restitution, (e) APD and CV restitution and curvature, and, (f) the full-physics PIE model with APD and CV restitution, curvature and diffusion effects. Only the S1 stimulus was delivered at a cycle length of 500 ms to initiate a planar wave front traversing the domain, to ascertain the same activation and repolarization pattern in all configurations. Activity was simulated over a time window of 1 s at a temporal output rate at 1 ms on the 32 core workstation for both models.

Scaling results are shown in Fig. 8B. Accounting for physiological features increased computational cost which reduced performance gains obtained with the PIE over the RD model, from a factor of 409 with (a) down to 46 with (f). Specifically, accounting for curvature and diffusion effects led to a marked increase in compute costs due to slower convergence rate and limited CPU-related parallel computing capabilities of the PIE solver. The ratio between PIE and RD solver execution time remained constant, indicating that scaling with problem size was comparable.

Runtime performance was quantitatively compared between the RD and the PIE, taking into account the time required for calibration, initialization and simulation of the whole-heart VT induction experiment. Simulating the RD model at 250 *μ*m required execution on the HPC cluster, while the PIE model was solved on the workstation at a resolution of 1000 *μ*m.

Performance measurements of simulating VT and associated ECG in a human whole-heart torso model are given in Supplementary Sec. C.4. Simulating 5 s of VT with the RD model required  ~ 17 h in total using 192 cores on the HPC cluster. The same simulation with the PIE model on the 1000 *μ*m mesh was executed within  ~ 4 min on the workstation using 32 cores. This corresponds to an effective speedup of  ~ 275 × while utilizing only 16% of the CPU cores with the PIE model.

Despite the vast speedup obtained, the performance achieved in the whole heart simulation at a discretization of 1000 *μ*m lagged real-time by a factor of  ~ 44 × when using the most accurate numerical settings. A further coarsening of mesh resolution led to a reduction in real-time lag by a factor of  ~ 7 × . When mesh coarsening preserved domain boundaries, ECG fidelity remained essentially unchanged (see Supplementary Sec. C.4). True real-time performance could readily be achieved with the PIE model by optimizing either numerical settings, e.g. increasing the time step of the Eikonal solver, or utilizing GPU acceleration. Overall, the computational performance of the PIE model enables high fidelity EP simulation on desktop machines within feasible time frames.

## Discussion

Our PIE modeling framework provides a lightweight approach for accurately simulating human CEP at the organ scale under a broad range of conditions in near real-time, with physical fidelity comparable to RD models, the gold-standard in CEP modeling. After a lightweight initial calibration where CEP key properties of the RD model are measured in small test setups and encoded in feature maps, the PIE model is able to replicate the most important emergent phenomena as predicted by a RD model such as wave front propagation dynamics and conduction blocks under varying heart rates as well as the induction and maintenance of both anatomical and functional reentrant arrhythmias, with high quantitative accuracy, at a fraction of the compute costs. The potential of the PIE model for clinical CDT applications was demonstrated by simulating a VT circuit, mediated by an infarct scar with isthmus and outer-loop topology as typically seen in VT patients^39^, in an anatomically accurate human whole-heart torso model.

The PIE model was shown to accurately match both the spatio-temporal dynamics of cardiac sources as well as the most prevalent clinically observable signal–the 12-lead ECG, which features a characteristic signal morphology linked to the discrete topological pathways of a VT circuit through the scar. The agreement between models was shown to be robust with respect to important numerical parameter choices such as mesh resolution. Our analyses showed that discrepancies remain small up to a mesh resolution of 3000 *μ*m, given that coarser meshes preserve the interface boundaries between domains of different EP properties of the higher resolution mesh used for RD simulations. Global coarsening not preserving interface boundaries induces topological changes that alter the VT circuit path length, and thus affect VT cycle and maintenance. For instance, even at the highest average resolution of 1000 *μ*m used for the PIE solver, the VT cycle length was affected, leading to a notable error accumulation over time. At a coarser resolution of 3000 *μ*m the VT was not sustained over the observation period (Supplementary Sec. C.3). For boundary-preserving mesh coarsening, discrepancies in predicted ECGs remained marginal in terms of morphology, magnitude and periodicity up to the coarsest resolution tested at 3000*μ*m. Overall, the observed model discrepancies can be considered marginal, particularly when viewed in the context of the significant overall uncertainty all model parameters of the high-dimensional RD model are afflicted with.

Based on its mechanistic completeness, accuracy, and computational speed, the PIE model is able to replace expensive high-fidelity RD models under a broad range of conditions, including arrhythmias. Combined with accurate predictions of observable signals, the PIE model provides the fundamental key technology for leveraging CDT applications for precision cardiology. A CDT for tailoring therapies relies on accurate calibration to ascertain that models operate within a tight physiological envelope matching a given patient^40,41^, and on physically complete models for correctly predicting the cardiac response to a variety of perturbations. Both calibration and predictive interrogation of a CDT constitute a many query problem, requiring a large number of forward simulation runs to constrain and sample the high dimensional physical parameter space. Due to the vast computational costs of RD models, requiring days to compute even with high performance computing resources, current clinical modeling studies based on RD models refrain from any patient-specific calibration^7,42^, and do not compute clinically observable signals to facilitate like-for-like comparisons between model and patient. Moreover, to keep simulations tractable, studies using RD models resort to coarse spatial discretization, which renders simulated arrhythmic behavior stochastic^12^.

The combined biophysical fidelity and real-time capability render the PIE model a competitive technology for realizing patient-specific predictive CEP models in time-critical clinical CDT applications geared toward tailoring therapies. Full-order RD models^43^ remain the gold-standard in terms of physiological fidelity, but even with optimized implementations using parallelization^6^ or hardware acceleration^8^ computational costs remain intractable, hindering their adoption in many-query CDT applications. Data-driven approaches emerging within the field of scientific machine learning have shown promise for generating lightweight surrogate CEP models that are sufficiently fast and accurate for many-query CDT applications. Model-order reduction or operator learning techniques^13,15,16,18^ may offer, in principle, the computational performance for solving CEP problems in near real-time. However, practicality and generalizability beyond toy problems remain to be investigated. Performance for generating solutions with sufficient spatio-temporal resolution with anatomically accurate organ models, as well as the reconstruction fidelity of observable signals, has not been shown yet. Moreover, the computational resources required for the generation of suitable training data are orders of magnitude more expensive compared to the time needed for calibrating the PIE model, which lasts only on the order of seconds. Further, unlike with the PIE model, where the initial calibration is universal, covering the full parameter space, with data-driven models, the parameter space to be interrogated must be narrowed down to keep off-line training tractable, which limits the flexibility for patient-specific CDT applications. The potential of real-time CEP modeling based on low-cost state-of-the-art Eikonal-based methods^24,27^ has been investigated, but these lacked physiological completeness to accurately replicate behaviors of full-order RD models under more general conditions, which limits their application scope.

While the PIE model covers a broad range of applications, it cannot be considered a universally applicable replacement of a RD model due to a number of important limitations. A major source of discrepancies between RD and PIE model relates to the mechanism used for approximating the effect of diffusion. Most pronounced differences were noted in simulations of functional reentry, where the PIE model was not able to accurately replicate the repolarization behavior around the phase singularity, which led to differences in the trajectory and shape of the rotor core. However, considering the highly non-linear and sensitive nature of functional reentrant activation, the overall emergent behavior was very comparable, and discrepancies were bounded within reasonable limits, even within the vicinity of the driving phase singularity. Moreover, it is important to note that an exact replication of functional arrhythmia simulations is currently also not feasible with RD models. Due to the highly non-linear nature, minor perturbations in model parameters, solver scheme and numerical settings such as solver tolerances or parallelization, discrepancies will inevitably emerge and grow over time. While such simulations are useful for the mechanistic interpretation of experimental data, caution is warranted in patient-specific modeling applications due to the vast model parameter uncertainty and the inherent stochasticity of model predictions. As functional reentry in the human heart is not directly observable clinically and model parameters are not constrained by measured data, evidence linking model predictions to patient physiology is lacking. In current clinical modeling studies that consider functional reentry, no attempts were undertaken to calibrate models to patient data, not even under baseline conditions^44^. Nevertheless, the broadly comparable behavior and the substantial computational advantage of the PIE may enable patient-specific assessments based on statistical analysis of large ensembles of representative simulations, acknowledging that individual simulations do not constitute one-to-one representations of a given patient.

Further discrepancies were attributable to stimulation and domain boundary effects on CV and APD. For instance, CV is reduced near initiation sites, increases during propagating toward a tissue boundaries, and wave fronts propagate perpendicular to boundaries due to no-flux boundary conditions. Boundary effects on electrotonic current influence repolarization and APD. These mechanisms are not captured in the PIE model.

Furthermore, no universally applicable formulation exists to prescribe restitution behavior solely as a function of the preceding diastolic interval, due to history dependence commonly referred to as cardiac memory^45^. The phenomenological representation of CV and APD restitution used by the PIE solver is based on measurements using the RD model under a chosen protocol. However, the choice of protocol captures different aspects of restitution dynamics. The dynamic protocol characterizes restitution at constant cycle length, whereas the S1-S2 protocol captures the immediate response to a DI perturbation from a steady-state limit cycle. The subsequent slow accommodation is described by constant-cycle restitution^46^. Thus, depending on the protocol used, predictions of APD and CV for the same DI differ^31^. As the sequence of DIs does not correspond to those observed in tissue-scale simulations, predictions of CV and APD differ between the PIE and RD models. However, the impact of these differences was marginal, as evidenced by the whole-heart VT simulations in which predictions of CV and APD remained in close agreement over a prolonged observation period.

A fundamental limitation of the current PIE model lies in its representation of cardiac tissue as a fully deterministic system characterized by static physiological properties measured only once during calibration (Fig. 1B). Consequently, inherently stochastic phenomena–such as the onset of alternans or spontaneous triggered activity arising from early or late afterdepolarizations, which can initiate arrhythmias–are not captured by the PIE model.

Further development is required to account for such phenomena. Several adaptation strategies are conceivable. For example, cellular dynamics models could be evaluated directly on-the-fly. However, this approach would incur substantial computational costs, resulting in markedly lower performance comparable to that of previous reaction-eikonal-type models^22,23^.

A more efficient approach could leverage higher-dimensional parameter-phenotype mappings that encode AP, APD, and CV dependence on parameters beyond DI, for example, using logistic regression to predict ectopic event probability^47^. Such extensions can be incorporated into the PIE framework, albeit with increased finite-state machine complexity and calibration cost. An alternative strategy is to employ surrogate reduced-order models to approximate cellular dynamics on-the-fly using Gaussian processes^48^ or machine learning^49^. This latter approach is particularly promising and the focus of ongoing and future investigations.

Independent of stochastic events at the cellular level, additional metrics of spatial coherence must be considered, as ectopy at the tissue scale is determined by the liminal length rather than by cellular triggering alone. Specifically, a sufficiently large population of cells must undergo spontaneous depolarization with adequate temporal synchrony to overcome the repolarizing influence of adjacent subthreshold tissue and initiate an ectopic activation^50^.

Finally, because the PIE model is statically calibrated under limit-cycle conditions, it does not readily generalize to non-equilibrium dynamics in which physiological properties evolve over time, such as transient responses following drug administration. These effects could be incorporated by periodically recalibrating the PIE model to update system properties (Fig. 1B), an approach that remains efficient since calibration is performed in single-cell or 1D strand models that are computationally inexpensive. Alternatively, a parameter-phenotype map could be employed to explicitly encode temporal evolution, at the cost of an increase in finite-state machine complexity.

Departing from this initial PIE implementation, further improvements in representing diffusion, in accounting for heterogeneity in cellular dynamics and its modulation by drugs, combined with technological advances to further enhance computational performance, are feasible and will be the topic of future research. Ultimately, the PIE strives to deliver full physical fidelity CDTs simulations with true real-time performance to support a broad spectrum of applications in precision cardiology, including intraprocedural online simulation.

## Methods

The PIE model builds upon three computational components: (i) a FSM encoding local cellular states, (ii) a wave propagation mechanism supporting repetitive activation, and (iii) an AP voltage recovery. The reconstruction of clinical signals is based on a lead-field approach and is described in Supplementary Sec. A.5. An overview of the functionality of each component of the PIE model and how each component attains physiological fidelity through calibration is given in Fig. 1. The complete algorithmic description of the PIE model is provided in Supplementary Sec. B.

### Encoding cellular states

**Fig. 9:**
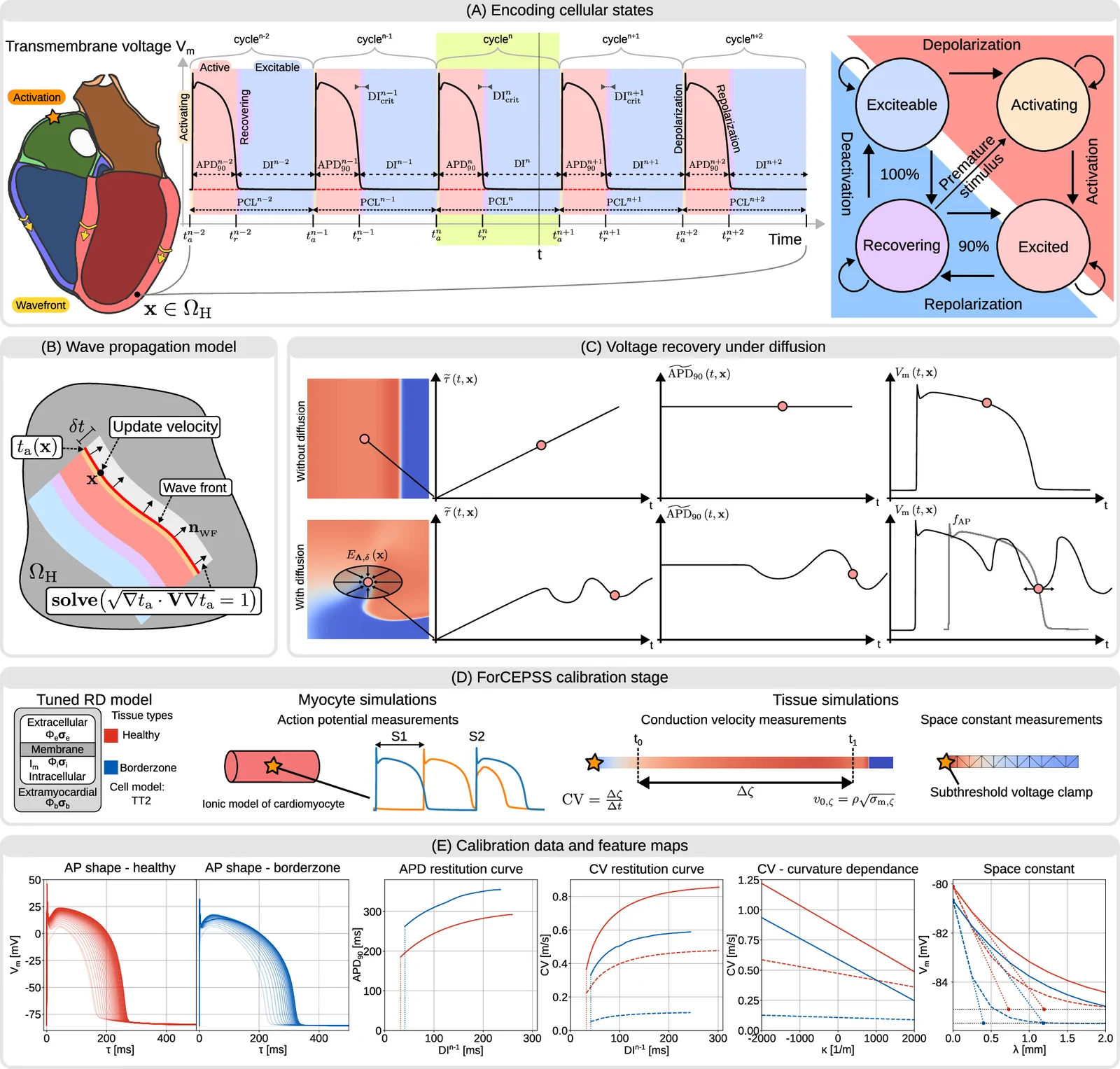
Detailed description of the PIE approximation methods. **A** Encoding cellular states using a FSM. **B** Reentrant Eikonal-based wave propagation mechanism. **C** Voltage recovery depending on diffusion. **D** Calibration of the PIE model using ForCEPSS by performing high-fidelity but low-cost cell- and tissue-level experiments. **E** Obtained PIE model calibration data, including AP shapes, APD and CV restitution curves, electrotonic curvature and space constant measurements employed by the diffusion mechanism.

In the heart domain *Ω*_H_, the traversal of wave fronts is dictated by the local electrical state of the tissue (Fig. 9A). Whether a wave front arriving at a site **x** ∈ *Ω*_H_ is propagated depends on the cellular state at **x**. If tissue at **x** ∈ *Ω*_H_ is in an excitable state and sufficient electrotonic current is provided, either by electrical stimulation or by an approaching wave front, to depolarize *V*_*m*_ (*t*, **x**) above the threshold *V*_th_, an active response is elicited in the form of an AP. This behavior is referred to as excitability. Once an AP has been elicited, the cell is in an excited but refractory state in which no new AP can be elicited over a refractory period, lasting until a cell recovers excitability. The states excitable and refractory can be considered mutually exclusive.

To control the excitability of cardiac cells within the domain *Ω*_H_, the PIE model employs a lightweight FSM that represents cardiac AP phases and the transitions between these. Similar to^25^, each AP cycle is divided into four phases indicated by a state variable, *s*, that represents the following physiological conditions:

- ACTIVATING: The short time interval of cellular depolarization comprising subthreshold capacitive loading, referred to as the foot of the AP, and the supra-threshold response manifesting in the fast upstroke of the AP.
- EXCITED: After activation, the cell transitions to an excited refractory state, during which the main depolarizing ion channels are inactivated, impeding the elicitation of an AP.
- RECOVERING: The cell is sufficiently repolarized to recover excitability, but a stronger stimulus is required for eliciting an AP.
- EXCITABLE: The cell is fully repolarized and depolarizing ion channels are recovered from inactivation, rendering cells excitable again.

Phase transitions of a cell located at **x** ∈ *Ω*_H_ within the *n*-th cardiac cycle are described based on the following notation:

- The LAT, ${t}_{{{{\rm{a}}}}}^{n}({{{\mathbf{x}}}})$, represents the instant of depolarization and marks the start of the *n*-th cycle.
- The PCL represents the interval between two supra-threshold stimuli, either delivered by a stimulus or by an approaching depolarization wave front. The PCL is determined at the end of a cycle terminated by a new activation ${t}_{{{{\rm{a}}}}}^{n}$, as
  1 $${{{{\rm{PCL}}}}}^{n-1}\left({{{\mathbf{x}}}}\right)={t}_{{{{\rm{a}}}}}^{n}\left({{{\mathbf{x}}}}\right)-{t}_{{{{\rm{a}}}}}^{n-1}\left({{{\rm{x}}}}\right).$$
- The refractory period of the *n*-th cycle denotes the time elapsed between complete activation until the instant at which the cell starts recovering excitability. The onset of the recovering phase, which depends on the state of ion channels, can be measured in any model of cellular dynamics. As this is closely linked to the repolarization of the AP to a given voltage level, it can be approximated by an AP-dependent surrogate marker, APD_90_, the instant at which the AP is repolarized to 90% of the AP amplitude. The end of the recovering phase, where excitability has been fully restored, is linearly approximated by APD_100_ = APD_90_/0.9. The onset of the recovering state of cycle *n* at location **x** ∈ *Ω*_H_ is denoted as LRT ${t}_{{{{\rm{r}}}}}^{n}({{{\mathbf{x}}}})$, given by
  2 $${t}_{{{{\rm{r}}}}}^{n}\left({{{\mathbf{x}}}}\right)={t}_{{{{\rm{a}}}}}^{n}\left({{{\mathbf{x}}}}\right)+{{{{\rm{APD}}}}}_{{{{\rm{90}}}}}^{n}\left({{{\mathbf{x}}}}\right).$$
- The DI refers to the interval between an instant of repolarization and the next activation:
  3 $${{{{\rm{DI}}}}}^{n-1}\left({{{\mathbf{x}}}}\right)={t}_{{{{\rm{a}}}}}^{n}\left({{{\mathbf{x}}}}\right)-{t}_{{{{\rm{r}}}}}^{n-1}\left({{{\mathbf{x}}}}\right).$$

To identify the current state of a cell located at **x** ∈ *Ω*_H_ at a given time $t\ge {t}_{{{{\rm{a}}}}}^{N}\left({{{\mathbf{x}}}}\right)$, where *N* = *N*(**x**) is a generic index referring to the current cycle, we introduce the relative cycle time $\tau :[{t}_{{{{\rm{a}}}}}^{N},\infty )\to [0,{T}_{{{{\rm{AP}}}}}]$ as

4 $$\tau \left( t,{{{\mathbf{x}}}} \right)=\min \left( {{{\mathrm{APD}}}}^{{{{\mathrm{ref}}}}}_{90} \, \frac{t - t^{N}_{a}({{{\mathbf{x}}}})}{{{{\mathrm{APD}}}}_{90}^{N}({{{\mathbf{x}}}})},T_{{{\mathrm{AP}}}}\right),$$

that maps the current solver time *t* to an cycle time *τ* defined for a prescribed one-dimensional AP template function ${f}_{{{{\rm{AP}}}}}:[0,{T}_{{{{\rm{AP}}}}}]\to {\mathbb{R}}$ with a basic cycle length of *T*_AP_ and a corresponding reference ${{{{\rm{APD}}}}}_{90}^{{{{\rm{ref}}}}}$. The cycle time is scaled by the ratio of the APD from the reference AP and the actual APD_90_, since the APD is altered over time due to physiological effects. Then, the cell state *s*(*t*, **x**) can be derived from *τ*(*t*, **x**) by

5 $$s\left(t,{{{\mathbf{x}}}}\right)=\left\{\begin{array}{ll}{\mathtt{ACTIVATING}}\quad &\,{{\mbox{if}}}\,\quad \tau \left(t,{{{\mathbf{x}}}}\right)=0,\hfill \\ {\mathtt{EXCITED}}\hfill &\,{{\mbox{if}}}\,\quad \tau \left(t,{{{\mathbf{x}}}}\right)\in (0,{{{{\rm{APD}}}}}_{90}^{{{{\rm{ref}}}}}],\hfill \\ {\mathtt{RECOVERING}}\quad &\,{{\mbox{if}}}\,\quad \tau \left(t,{{{\mathbf{x}}}}\right)\in ({{{{\rm{APD}}}}}_{90}^{{{{\rm{ref}}}}},\frac{{{{{\rm{APD}}}}}_{90}^{{{{\rm{ref}}}}}}{0.9}],\\ {\mathtt{EXCITABLE}}\hfill &\,{{\mbox{if}}}\,\quad \tau \left(t,{{{\mathbf{x}}}}\right)\in (\frac{{{{{\rm{APD}}}}}_{90}^{{{{\rm{ref}}}}}}{0.9},{T}_{{{{\rm{AP}}}}}]\hfill\end{array}\right.$$

for $t\ge {t}_{{{{\rm{a}}}}}^{N}\left({{{\mathbf{x}}}}\right)$ (see right panel of Fig. 9A). State transitions depend on relative cycle time *τ* and ${{{{\rm{APD}}}}}_{90}^{N}$ which, in turn, is modulated by the preceding DI and diffusion, both dictated by wave front dynamics. The dependency ${{{{\rm{APD}}}}}_{90}^{N}({{{{\rm{DI}}}}}^{N-1})$, referred to as action potential restitution, must be quantitatively measured in a full-order system during a calibration step, and can be used then to encode state transitions.

### Reentrant wave propagation model

Wave front propagation in the myocardium *Ω*_H_, as described by the bidomain model, can be expressed as a traveling wave front problem^20^. Given stimulation sites **x**_s_ ∈ *Ω*_S_ ⊂ *Ω*_H_ where wave propagation is initiated at instances *t*_s_(**x**_s_), a traveling wave front solution can be obtained by solving the anisotropic Eikonal equation

6 $$\sqrt{\nabla {t}_{{{{\rm{a}}}}}\,\cdot \,{{{\bf{V}}}}\,\nabla {t}_{{{{\rm{a}}}}}}=1\quad \,{\mbox{in}}\,\quad {{{\mathbf{x}}}}\in {\Omega }_{{{{\rm{H}}}}}\backslash {\Omega }_{{{{\rm{S}}}}}$$

7 $${t}_{{{{\rm{a}}}}}={t}_{{{{\rm{s}}}}}\quad \,{\mbox{in}}\,\quad {{{{\mathbf{x}}}}}_{{{{\rm{s}}}}}\in {\Omega }_{{{{\rm{S}}}}}$$

which yields the wave front arrival times *t*_a_(**x**) in *Ω*_H_. The Eikonal model is coupled to the FSM, which dynamically restricts the solution domain *Ω*_H_ to areas where tissue is in an excitable state^25^. Eq. (6) is incrementally solved over a short moving time horizon *δt* over the restricted domain (Fig. 9B). As tissue at **x** may transition from an excited to an excitable state, as dictated by the physiology of cellular dynamics encoded in the FSM, cells in *Ω*_H_ can be re-excited multiple times, thus providing the fundamental mechanism for supporting repetitive activation and self-sustained reentrant wave front propagation.

Propagation velocity of the wave front is controlled by the symmetric squared velocity tensor ${{{\bf{V}}}}\,\in \,{{\mathbb{R}}}^{3\times 3}$, defined by

8 $${{{\bf{V}}}}={v}_{f}^{2}\,\left({{{\bf{f}}}}\otimes {{{\bf{f}}}}\right)+{v}_{s}^{2}\,\left({{{\bf{s}}}}\otimes {{{\bf{s}}}}\right)+{v}_{n}^{2}\,\left({{{\bf{n}}}}\otimes {{{\bf{n}}}}\right),$$

where *v*_*f*_, *v*_*s*_, and *v*_*n*_ represent orthotropic CVs along the eigenaxes of tissue, referred to as fiber, **f**(**x**), sheet, **s**(**x**), and sheet-normal, **n**(**x**), direction, respectively. The intrinsic CV of wave fronts may be altered by the rate of activation and by wave front curvature. With faster activation rates CV reduces as sodium channels are increasingly less recovered from inactivation at short DIs. This behavior is referred to as CV restitution which can be characterized by measuring the function CV^*N*^(DI^*N*−1^). At the tissue scales wave front curvature modulates CV by altering the diffusive source-sink ratio^21,51^ which increases/reduces CV for concave/convex wave fronts, respectively.

To account for both effects, we assume that each factor acts independently, allowing to approximate the resulting CV of a wave front in propagation direction **n**_WF_, arriving at **x** ∈ *Ω*_H_ at the instant of local activation, $t={t}_{{{{\rm{a}}}}}^{N}({{{\mathbf{x}}}})$, in the current cycle *N* = *N*(**x**) by modifying the CV *v*_*ζ*_ along each anisotropy axis *ζ* ∈ {**f**, **s**, **n**} as

9 $${v}_{\zeta }={v}_{0,\zeta }\cdot {f}_{{{{\rm{CVC}}}}}\left(\kappa \right)\cdot {f}_{{{{\rm{CVR}}}},\zeta }\left({{{{\rm{DI}}}}}^{N-1}\right),$$

where *v*_0,*ζ*_ represents the nominal CV of a planar wave front measured at long DIs along the direction *ζ*. *f*_CVR,*ζ*_ is a relative normalized representation of the CV restitution function measured for planar wave fronts propagating in direction *ζ* at variable DIs, and *f*_CVC_ is a function quantifying the CV dependence on anisotropic curvature *κ*. Specifically, following^20,21,52^ we model the linear dependency of the CV on the anisotropic curvature as

10 $${f}_{{{{\rm{CVC}}}}}\left(\kappa \right)=\left(1-\frac{\kappa }{\rho \beta {C}_{{{{\rm{m}}}}}}\right)\quad \,{\mbox{with}}\,\quad \kappa=\nabla \cdot \left(\frac{{{{{\boldsymbol{\sigma }}}}}_{{{{\rm{m}}}}}\,{{{{\bf{n}}}}}_{{{{\rm{WF}}}}}}{\sqrt{{{{{\bf{n}}}}}_{{{{\rm{WF}}}}}\cdot {{{{\boldsymbol{\sigma }}}}}_{{{{\rm{m}}}}}\,{{{{\bf{n}}}}}_{{{{\rm{WF}}}}}}}\right)$$

where the wave front normal pointing in the direction of the propagation is given by

$${{{{\bf{n}}}}}_{{{{\rm{WF}}}}}=\frac{\nabla {t}_{{{{\rm{a}}}}}^{N}}{\parallel \nabla {t}_{{{{\rm{a}}}}}^{N}\parallel }$$

and *ρ* being the proportionality constant between CV of a planar wave front traveling along **n**_WF_ and the tissue conductivity along the same axis (Supplementary Sec. A.4). For a negative *κ*, which is associated with concave wave fronts, CV increases, while for a positive *κ*, which is associated with convex wave fronts, CV decreases relative to the velocity of a planar wave front with *κ* = 0. At a critical positive curvature *κ*_*ζ*,crit_ wave front propagation cannot be maintained and breaks down due to an unfavorable source-sink mismatch.

CV restitution curves as measured in reference RD models with suitable protocols^28^ feature a non-linear monotonically decreasing function with increasingly pronounced reduction at shorter DIs, until propagation failure occurs at a critically short DI. The relative normalized CV restitution function ${f}_{{{{\rm{CVR}}}},\zeta }:{\mathbb{R}}\to [0,1]$ is determined by measuring CV(DI), and scaling with the reference CV measured at a resting pacing cycle length. For slower pacing cycle lengths leading to increasingly larger DI it is assumed that *f*_CVR_ follows

11 $${{\lim }_{{{{\rm{DI}}}}\to \infty }}{f}_{{{{\rm{CVR}}}},\zeta }\left({{{\rm{DI}}}}\right)=1.$$

### Voltage recovery

The time course and duration of the AP at any **x** ∈ *Ω*_H_ are governed by the intrinsic cellular properties, the preceding diastolic interval DI^*N*−1^, and, electrotonic currents acting during repolarization to shorten or prolong APD. These functional relations AP(*t*, DI^*N*−1^) and APD^*N*^(DI^*N*−1^) can be measured in a reference RD model and, combined with known activation time *t*_a_ and anisotropic curvature *κ* of a wave front arriving at **x**, used to dictate state transitions in the FSM. Thus, with known instants of state transitions, *t*_a_ and *t*_r_, the temporal history-dependent evolution of *V*_*m*_ (*t*, **x**) at any **x** ∈ *Ω*_H_ can be recovered (Fig. 9C).

The implemented voltage recovery procedure assumes that the restitution and diffusion influence the AP independently. The restitution effect on the AP at location **x** ∈ *Ω*_H_ in the current cycle *N* = *N*(**x**) alters AP shape, *V*_*m*_(*t*), and duration, APD, as a function of the preceding DI^*N*−1^. This dependency is measured for each domain of differing EP properties by pacing a single cell according to a restitution protocol which is readily achieved, at low cost, by solving the system of ordinary differential equations (ODEs) given by Supplementary Eq. (A14). The measured restitution curve is stored then as a function

12 $${{{{\rm{APD}}}}}_{{{{\rm{90}}}}}^{{{{\rm{ref}}}}}={f}_{{{{\rm{APD}}}}}\left({{{{\rm{DI}}}}}^{N-1}\right)$$

and the precomputed AP wave profiles, *f*_AP_, with a cycle length, *T*_AP_, are stored as a two-dimensional table

13 $${V}_{m}(\tau )={f}_{{{{\rm{AP}}}}}\left(\tau,\,{{{{\rm{APD}}}}}_{{{{\rm{90}}}}}^{{{{\rm{ref}}}}}\right),$$

where each APD obtained from the restitution function *f*_APD_ corresponds to a specific AP profile *V*_*m*_(*τ*) within the table ${f}_{{{{\rm{AP}}}}}:[0,{T}_{{{{\rm{AP}}}}}]\times {{\mathbb{R}}}_{+}\to {\mathbb{R}}$. The voltage at site **x** within cycle *N*(**x**) at cycle time *τ*(*t*, **x**) as per Eq. (4), is recovered then by determining first ${{{{\rm{APD}}}}}_{{{{\rm{90}}}}}^{{{{\rm{ref}}}}}({{{\mathbf{x}}}})$ from DI^*N*−1^(**x**), and looking up the current value of *V*_m_ in Eq. (13). If diffusive effects are deemed negligible, Eq. (13) can be used to recover *V*_m_ as APD is fully determined at the moment of activation due to the linear progression of the cycle time *τ* (Fig. 9C, upper panels). In this case, the procedure of voltage recovery is a post-processing step, that is entirely independent of the FSM and the Eikonal-driven wave front propagation within the PIE implementation. Since the solution of the system of ODEs which is expensive at the tissue scale, is avoided, and only a comparably cheap solution at a single cell level is required, carried out only once in a pre-processing step, the computational costs incurring with voltage recovery are negligible.

Diffusive electrotonic currents act (i) during depolarization of the AP to mediate propagation of the wave front, and (ii) during repolarization to shorten or prolong APD, associated with changes in the AP wave form (Fig. 9C, bottom panels). The role of diffusion in propagation (i) does not require further attention as its physical effect is already accounted for by the Eikonal equation, Eq. (6), which is able to approximate the propagation of wave front propagation on much coarser computational grids than feasible with a RD model. Diffusion during repolarization (ii) is taken into account by phenomenologically approximating the main effect observed in RD models – an anisotropic smoothing of the repolarizing wave back. Thus, diffusion strongly influences clinically observable extracellular signals such as the ECG by attenuating transmembrane voltage gradients  ∇ *V*_*m*_(**x**) as seen in Supplementary Eq. (A18). In the PIE model these effects are approximated by evaluating the AP template function during voltage recovery in Eq. (13) with locally smoothed state variables, that is

14 $${V}_{m}(t,{{{\mathbf{x}}}})={f}_{{{{\rm{AP}}}}}\left(\widetilde{\tau }\left(t,{{{\mathbf{x}}}}\right),{\widetilde{{{{\rm{APD}}}}}}_{90}(t,{{{\mathbf{x}}}})\right),$$

where $\widetilde{\tau }(t,{{{\mathbf{x}}}})$ is a smoothed cycle time, suitably averaged over the volume influenced by electronic currents.

To keep the diffusion mechanism within the PIE model lightweight, we approximate global diffusion of RD models with local diffusion^53^ based on the observation that any local perturbation decays exponentially in space where the rate of decay along a direction *ζ* is governed by the electrotonic space constant, *λ*_*ζ*_. The volume of electrotonic influence is therefore bounded by an elliptic subdomain ${E}_{{{{\mathbf{\Lambda }}}},\delta }\left({{{\mathbf{x}}}}\right)\subset {\Omega }_{{{{\rm{H}}}}}$ around **x** ∈ *Ω*_H_ defined by

15 $${E}_{{{{\mathbf{\Lambda }}}},\delta }\left({{{\mathbf{x}}}}\right)=\left\{{{{\bf{y}}}}\in {\Omega }_{{{{\rm{H}}}}}\left\vert \right.{\left({{{\mathbf{x}}}}-{{{\bf{y}}}}\right)}^{\top }{{{{\mathbf{\Lambda }}}}}^{-1}\left({{{\mathbf{x}}}}-{{{\bf{y}}}}\right)\le \delta \right\},$$

with the space constant tensor defined as ${{{\mathbf{\Lambda }}}}={\lambda }_{f}\,\left({{{\bf{f}}}}\otimes {{{\bf{f}}}}\right)+{\lambda }_{s}\,\left({{{\bf{s}}}}\otimes {{{\bf{s}}}}\right)+{\lambda }_{n}\,\left({{{\bf{n}}}}\otimes {{{\bf{n}}}}\right)$, where *λ*_*ζ*_ are the orthotropic space constants along each eigenaxis *ζ*. The smoothed action potential duration ${\widetilde{{{{\rm{APD}}}}}}_{90}^{N}$ and smoothed cycle time $\widetilde{\tau }$ are solutions to local anisotropic diffusion problems, given by

16 $${\widetilde{{{{\rm{APD}}}}}}_{90}(t,{{{\mathbf{x}}}})=\int\limits_{{E}_{{{{\mathbf{\Lambda }}}},\delta }\left({{{\mathbf{x}}}}\right)}K(t-{t}_{{{{\rm{a}}}}}^{N}({{{\mathbf{x}}}}),{{{\mathbf{x}}}}-{{{\bf{y}}}})\,{{{{\rm{APD}}}}}_{90}^{{{{\rm{ref}}}}}({{{\bf{y}}}})\ {{{\rm{d}}}}{{{\bf{y}}}}$$

and

17 $$\widetilde{\tau }(t,{{{\boldsymbol{x}}}})=\min \left(\int\limits^{t}_{t^{N}_{{{{\rm{a}}}}}({{{\rm{x}}}})}\int\limits_{{E}_{{{{\mathbf{\Lambda }}}},\delta }\left({{{\boldsymbol{x}}}}\right)}K(t-s,{{{\boldsymbol{x}}}}-{{{\bf{y}}}})\,\frac{{{{{\rm{APD}}}}}^{{{{\rm{ref}}}}}_{90}({{{\bf{y}}}})}{{\widetilde{{{{\rm{APD}}}}}}_{90}(s,{{{\bf{y}}}})}\ {{{\rm{d}}}}{{{\bf{y}}}}\,{{{\rm{d}}}}s,{T}_{{{{\rm{AP}}}}}\right)$$

for **x** ∈ *Ω*_H_ and $t > {t}_{{{{\rm{a}}}}}^{N}({{{\mathbf{x}}}})$. Diffusivity within the subdomain is controlled by the anisotropic heat kernel

18 $$K(t,{{{\mathbf{x}}}})=\frac{1}{\sqrt{{\left(4\pi t\right)}^{3}| \det {{{\bf{D}}}}| }}\exp \left(-\frac{{{{{\mathbf{x}}}}}^{\top }{{{{\bf{D}}}}}^{-1}{{{\mathbf{x}}}}}{4t}\right).$$

using a locally constant, symmetric, and invertible diffusion tensor ${{{\bf{D}}}}\in {{\mathbb{R}}}^{3\times 3}$ with its eigenvalues given by

19 $${D}_{\zeta }=\frac{{\sigma }_{m,\zeta }}{\beta {C}_{{{{\rm{m}}}}}},$$

derived from the harmonic-mean conductivity tensor of the RD model in Supplementary Eq. (A16). Without diffusion, *δ* = 0, the Eqs. (16) and (17) reduce to the previous Eqs. (12) and (4).

### Model calibration

The calibration stage of the PIE model measures individual EP properties for each electrically viable tissue type in the reference RD model (Fig. 9D), and maps these onto the PIE model as prescribed functions and constants (Fig. 9E). Specifically, AP shapes, APD and CV restitution curves, as well as diffusive space constants, were quantitatively measured in cellular and tissue-level experiments using the ForCEPSS^28^ framework:

- APD restitution curves *f*_APD_ were measured in single cells following a S1-S2 protocol where a stable limit cycle was approximated using a train of 20 stimuli, delivered at a S1 cycle length of 600 ms (Fig. 9D). At premature S2 cycles, APD^*N*^, and preceding DI^*N*−1^ were measured to construct *f*_APD_ according to Eq. (12). Premature S2 AP shapes were stored in a two-dimensional lookup table (Fig. 9E, leftmost panel), as given in Eq. (13).
- CV restitution was measured in 3D strand models along each eigenaxis *ζ* using a dynamic pacing protocol^28^. Starting at a PCL of 600 ms, the PCL was gradually shortened in decrements of 25 ms until loss of capture occurred. CV was determined by measuring the difference in wave front arrival times, Δt, between two reference location along the strand, placed at a distance Δ*ζ*, yielding the eigenvelocities *v*_*ζ*_ = Δ*ζ*/Δt (Fig. 9D). The diastolic interval DI^*N*−1^ was measured at the center of the strand to construct CV restitution curves *f*_CV R,*ζ*_ (Fig. 9E), as given in Eq. (11).
- The dependency of the CV on the anisotropic curvature, described by the function *f*_CVC_ given in Eq. (10), was determined by deriving *ρ* from the proportional relationship between the harmonic mean conductivities and the measured planar wavefront velocities in each axis (described in Supplementary Sec. A.4). The conduction properties, the surface-to-volume ratio *β* and the membrane capacitance *C*_m_ were provided by the calibrated reference RD model.
- The strength of local diffusion, described by the diffusion coefficients along each tissue eigenaxes D_*ζ*_ according to Eq. (18), were derived from the relationship given in Eq. (19) with the parameters of the calibrated reference RD model. Eigenvalues *λ*_*ζ*_ of the space constant tensor ***Λ*** bounding the volume of local diffusion in Eq. (15) were determined in 3D strand models under subthreshold conditions, without triggering wave front propagation, by applying a  + 5 mV voltage clamp. After arriving at a steady state, *λ*_*ζ*_ was estimated as the exponential decay rate in space of *V*_*m*_(**x**) (Fig. 9E, rightmost panel).

### Error metrics

The similarity between obtained multichannel ECGs was measured using the PCC^37,38^ and the DTW distance measure^35,36^. Since the ECG simulations featured the same start time, end time and time step, all measured ECG signals were aligned and of equal length. Given two discrete time series of equal length *N*, **A** and **B**, the PCC between both time series is computed by

$${{{\rm{PCC}}}}\left({{{\bf{A}}}},{{{\bf{B}}}}\right)=\frac{1}{N-1} {\sum }_{i=1}^{N}\left(\frac{{A}_{i}-{\mu }_{A}}{{\sigma }_{A}}\right)\left(\frac{{B}_{i}-{\mu }_{B}}{{\sigma }_{B}}\right),$$

where each time series is normalized with respect to their means, *μ*_*A*_ and *μ*_*B*_, and standard deviations, *σ*_*A*_ and *σ*_*B*_.

The DTW distance measure between the two given time series represents the solution to the optimization problem

$${{{\rm{DTW}}}}\left({{{\bf{A}}}},{{{\bf{B}}}}\right)={{\min }_{p\in {{{\mathcal{P}}}}({{{\bf{A}}}},{{{\bf{B}}}})}}\sqrt{\sum\limits_{(i,j)\in p}d{\left({A}_{i},{B}_{j}\right)}^{2}},$$

where $p\in {{{\mathcal{P}}}}$ represents all possible alignments described as paths spanned by the two time series **A** and **B**. A path represents a sequence of index pairs where the cost to reach each index pair is given by the recursive distance function

$$d{\left({A}_{i},{B}_{j}\right)}^{2}={\left\Vert {A}_{i}-{B}_{j}\right\Vert }_{2}^{2}+\min \left(d{\left({A}_{i-1},{B}_{j-1}\right)}^{2},d{\left({A}_{i},{B}_{j-1}\right)}^{2},d{\left({A}_{i-1},{B}_{j}\right)}^{2}\right).$$

Furthermore, valid paths of length *K* must satisfy the start- and endpoint condition, *p*_0_ = (0, 0) and *p*_*K*−1_ = (*N* − 1, *N* − 1), and the initial condition *d*_0_ = 0. Following^36^, we accumulated the DTW distance measures over each subsequence of each ECG lead signal, where subsequences were extracted based on local peak detection. In our specific case, we decided to omit the z-normalization of each subsequence in order to quantify and compare DTW distance measures in mV.

For the analysis of dominant frequency maps in the functional reentry experiment (E), the PSD was computed over each discrete nodal transmembrane voltage trace using Welch’s method^54,55^. Given the *m*-th windowed, zero-padded frame from a signal *x* of length *N*, where *x*_*m*_[*n*] = *w*[*n*] × [*n* + *mR*] with window size *M* and hop size *R*, the periodogram for each frame *k* ∈ *K*, given by

20 $${P}_{{x}_{m},M}\left({\omega }_{k}\right)=\frac{1}{M}{\left\vert {{\mbox{FFT}}}_{N,k}\left({x}_{m}\right)\right\vert }^{2},$$

is averaged over time to estimate the PSD, given by

21 $${\hat{S}}_{x}\left({\omega }_{k}\right)=\frac{1}{K} {\sum }_{m=0}^{K-1}{P}_{{x}_{m},M}\left({\omega }_{k}\right).$$

### Mesh generation and solvers

The meshes employed in the experiments simulating anatomical and the functional reentry in 3D slab models and the detailed validation experiments in Supplementary Sec. C were generated using mesher and processed using MeshTool^56^, as provided in ref. ^43^. Coarse- and fine-resolution whole heart models were carried over from our previous study^41^. Utilizing universal ventricular coordinates^57^, the scar region was mapped from a pig heart^58^ into the left ventricle of the coarse whole heart model. Since the fine and coarse models almost superimpose spatially, elements in the fine model that lie within the scar area of the coarse model were simply retagged.

All RD simulations were solved using the finite element solver framework openCARP^43^, configured with an implicit-explicit Crank-Nicolson scheme with operator-splitting and mass-lumping turned off, and solved iteratively^8^ using a time step of *dt* = 25 *μ*s (see openCARP user’s manual, pp. 135). The complete parameter set is deposited in the GitHub database under https://github.com/medunigraz/PIE-Model-Experiments and in the Zenodo database^59^ under accession code (https://doi.org/10.5281/zenodo.17198150).

### Whole-heart model acquisition and ethical statement

This study involved only computational modeling using a previously developed anatomical model^41^ and did not involve the recruitment of participants or the collection of new human data. Consequently, participant demographic characteristics, including race and ethnicity, were not relevant to this study. The study complies with applicable research ethics requirements.

### Statistics and reproducibility

This study presents a computational methodology based on an anatomical model of a single male subject. As no biological or experimental samples were analyzed and no statistical comparisons were performed, no statistical methods were used to predetermine sample size. No data were excluded from the analyses and the experiments were not randomized.

The software for reproducing all experimental results is publicly available in the GitHub database under https://github.com/medunigraz/PIE-Model-Experiments and in the Zenodo database^59^ under accession code (https://doi.org/10.5281/zenodo.17198150). All reference results can be reproduced with open source simulation software openCARP. For details we refer to the Data and Code Availability sections.

### Reporting summary

Further information on research design is available in the Nature Portfolio Reporting Summary linked to this article.

## Supplementary information


Supplementary Information
Reporting Summary
Transparent Peer Review file


## Data Availability

The whole-heart model used in this study was carried over from a previous study^41^ which is publicly accessible under https://doi.org/10.1016/j.media.2021.102080. All experimental setups employed in this study and its supplementary material, the calibration data of the RD and the PIE model, the generated ECGs and rendered videos of each experiment are publicly available in the GitHub database under (https://github.com/medunigraz/PIE-Model-Experiments). A complete dataset including the calibration data, the generated meshes, the anonymized human whole-heart model and respective lead-field data, computed simulation data and rendered videos of each experiment have been deposited in the Zenodo database^59^ under accession code (https://doi.org/10.5281/zenodo.17198150).

## Code availability

All reference experiments using the RD model were simulated with the openCARP framework (version 18.0), which includes the software for the finite element solver, mesh generation, manipulation and the measurement of physiological features encoded in ForCEPSS^28^. All codes for replication of RD simulations are freely available through (https://opencarp.org/download/). The code for the evaluation of the experiments is publicly available in the GitHub database under https://github.com/medunigraz/PIE-Model-Experiments and in the Zenodo database^59^ under accession code (https://doi.org/10.5281/zenodo.17198150). The implementation of the PIE solver (version 1.0) requires proprietary libraries which cannot be publicly disclosed. Binary executables compiled for the major platforms (Linux, macOS or Windows) can be provided upon request within a complete Docker image for replicating the results of this study.
