# Supplementary material for: A physics-informed eikonal model for simulating arrhythmias in the human heart in real-time
Source: Nat Commun. 2026 Jul 29;17:9209. doi: 10.1038/s41467-026-76050-0 (PMC13522352; doi:10.1038/s41467-026-76050-0)
Supplement: Supplementary file 1 [file 41467_2026_76050_MOESM1_ESM.pdf]

Supplementary Information: A Physics-Informed Eikonal Model for Simulating Arrhythmias in the Human Heart in Real-Time

Thomas G. Schrotter[1,2], Matthias A. F. Gsell[1], Elena Zappon[1], Christoph M. Augustin[1,2,3], Aurel Neic[2], Simone Pezzuto[4,5], Gernot Plank[1,2,3*]

[1*]Gottfried Schatz Research Center, Division of Medical Physics and Biophysics, Medical University of Graz, Neue Stiftingtalstraße 6, 8010, Graz, Austria.
[2]NumeriCor GmbH, Conrad-von-Hötzendorfstraße 26/2, 8010, Graz, Austria.
[3]BioTechMed-Graz, Mozartgasse 12/II, 8010, Graz, Austria.
[4]Department of Mathematics, University of Trento, Via Sommarive 14, 38123, Trento, Italy.
[5]Euler Institute, Università della Svizzera italiana, Via Buffi 13, 6900, Lugano, Switzerland.

Appendix A Models of Cardiac Electrophysiology

A.1 Bidomain Model

The cardiac reaction-diffusion (RD) bidomain model [1] provides the most physiologically-realistic representation of cardiac electrophysiology (CEP) at the tissue and organ scale. It accounts for the effects of the extracellular potential field upon wave front dynamics through representation of a surrounding conductive media, such as the blood pool or torso. Let $\Omega_{\mathrm{H}} \subset \mathbb{R}^3$ be a heart entirely immersed in a conductive bath $\Omega_{\mathrm{B}} \subset \mathbb{R}^3$ such that $\Omega = \Omega_{\mathrm{H}} \cup \Omega_{\mathrm{B}}$. The bidomain equations state that transmembrane currents, described by the current density I_{m} (in $\mathrm{A\,m^{-2}}$), enter intracellular and extracellular spaces by crossing the cell membrane. This produces sources of intracellular and extracellular potentials, Φ_{i} and Φ_{e}, governed by the system

$$\nabla \cdot \boldsymbol{\sigma}_{\mathrm{i}} \nabla \Phi_{\mathrm{i}} = \beta I_{\mathrm{m}} \qquad \text{in } \Omega_{\mathrm{H}}, \tag{A1}$$

$$\nabla \cdot \boldsymbol{\sigma}_{\mathrm{e}} \nabla \Phi_{\mathrm{e}} = -\beta I_{\mathrm{m}} - I_{\mathrm{ei}} \qquad \text{in } \Omega_{\mathrm{H}}, \tag{A2}$$

$$I_{\mathrm{m}} = C_{\mathrm{m}} \frac{\partial V_{\mathrm{m}}}{\partial t} + I_{\mathrm{ion}}\left(V_{\mathrm{m}}, \boldsymbol{\eta}\right) - I_{\mathrm{s}} \qquad \text{in } \Omega_{\mathrm{H}}, \tag{A3}$$

$$\frac{\partial \boldsymbol{\eta}}{\partial t} = f\left(\boldsymbol{\eta}, t\right) \qquad \text{in } \Omega_{\mathrm{H}}, \tag{A4}$$

$$\nabla \cdot \boldsymbol{\sigma}_{\mathrm{b}} \nabla \phi_{\mathrm{e}} = -I_{\mathrm{eb}} \qquad \text{in } \Omega_{\mathrm{B}}, \tag{A5}$$

yielding the transmembrane voltage $V_{\mathrm{m}} = \Phi_{\mathrm{i}} - \Phi_{\mathrm{e}}$ (in V) at every point in space and time. The anisotropic conductive tissue properties in intracellular and extracellular spaces depend on cardiac fiber orientation and are described by symmetric positive semi-definite tensors, $\boldsymbol{\sigma}_* \in \mathbb{R}^{3\times 3}$ with $* \in \{\mathrm{i}, \mathrm{e}\}$ (in $\mathrm{S\,m^{-1}}$), given by

$$\boldsymbol{\sigma}_* = \sigma_{*,\mathrm{f}}\ (\mathbf{f} \otimes \mathbf{f}) + \sigma_{*,\mathrm{s}}\ (\mathbf{s} \otimes \mathbf{s}) + \sigma_{*,\mathrm{n}}\ (\mathbf{n} \otimes \mathbf{n})\,, \tag{A6}$$

where $\mathbf{f}(\mathbf{x})$, $\mathbf{s}(\mathbf{x})$, $\mathbf{n}(\mathbf{x}) \in \mathbb{R}^3$, $\mathbf{x} \in \Omega_{\mathrm{H}}$, are the orthonormal eigenaxes along the fiber, sheet, and sheet-normal direction, with $\sigma_{*,\mathrm{f}}$, $\sigma_{*,\mathrm{s}}$ and $\sigma_{*,\mathrm{n}}$ as the associated conductivities, respectively. $\boldsymbol{\sigma}_{\mathrm{b}}$ describes the extracellular conductivity tensor in the bath Ω_{B}. The surface-to-volume ratio β (in $\mathrm{m^{-1}}$) and the capacitance per unit area C_{m} (in $\mathrm{F\,m^{-2}}$) represent properties of the cardiac cell membrane. The cellular dynamics are represented by a system of ordinary differential equations (ODEs), as shown in Eq. (A3), where the membrane ionic current density, I_{ion} (in $\mathrm{A\,m^{-2}}$), is governed by a set of ionic state variables, $\boldsymbol{\eta}$. I_{ei} and I_{eb} emerge from extracellular stimuli applied in the interstitial space or the bath, respectively, while I_{s} represents a transmembrane stimulus current density (in $\mathrm{A\,m^{-2}}$). At the tissue-bath interface, that is the interface between interstitial space of the myocardium and the blood pool or the surrounding torso, $\Gamma_{\mathrm{HB}} = \overline{\Omega_{\mathrm{H}}} \cap \overline{\Omega}_{\mathrm{B}}$, continuity of Φ_{e} and the normal component of the extracellular current is enforced, i.e.

$$\Phi_{\mathrm{e}}|_{\Gamma_{\mathrm{HB}}^-} = \Phi_{\mathrm{e}}|_{\Gamma_{\mathrm{HB}}^+} \qquad \text{on } \Gamma_{\mathrm{HB}}, \tag{A7}$$

$$\mathbf{n}_{\mathrm{hb}} \cdot (\boldsymbol{\sigma}_{\mathrm{i}} + \boldsymbol{\sigma}_{\mathrm{e}})\, \nabla \Phi_{\mathrm{e}}|_{\Gamma_{\mathrm{HB}}^-} = \mathbf{n}_{\mathrm{hb}} \cdot \boldsymbol{\sigma}_{\mathrm{b}} \nabla \Phi_{\mathrm{e}}|_{\Gamma_{\mathrm{HB}}^+} \qquad \text{on } \Gamma_{\mathrm{HB}}, \tag{A8}$$

with normal vector $\mathbf{n}_{\mathrm{hb}}$ pointing from Ω_{H} to Ω_{B}. The intracellular space is sealed, which is approximated by imposing no flux boundary conditions on Φ_{i},

$$\mathbf{n}_{\mathrm{hb}} \cdot \boldsymbol{\sigma}_{\mathrm{i}} \nabla \Phi_{\mathrm{i}} = 0 \qquad \text{on } \Gamma_{\mathrm{HB}}. \tag{A9}$$

At the boundaries formed by the surrounding medium, i.e. either the body surface or the boundary of a bath $\Gamma_{\mathrm{B}} = \partial\Omega$, no flux boundary conditions for Φ_{e} are imposed

$$\mathbf{n}_{\mathrm{b}} \cdot \boldsymbol{\sigma}_{\mathrm{b}} \nabla \Phi_{\mathrm{e}} = 0 \qquad \text{on } \Gamma_{\mathrm{B}}, \tag{A10}$$

with normal outer vector $\mathbf{n}_{\mathrm{b}}$. Eqs. (A1)–(A5) can be recast and solved in the elliptic-parabolic form, given by

$$\begin{bmatrix} -\nabla \cdot (\boldsymbol{\sigma}_{\mathrm{i}} + \boldsymbol{\sigma}_{\mathrm{e}}) \nabla \Phi_{\mathrm{e}} \\ -\nabla \cdot \sigma_{\mathrm{b}} \nabla \Phi_{\mathrm{e}} \end{bmatrix} = \begin{bmatrix} \nabla \cdot \boldsymbol{\sigma}_{\mathrm{i}} \nabla V_{\mathrm{m}} + I_{\mathrm{ei}} \\ I_{\mathrm{eb}} \end{bmatrix}, \tag{A11}$$

$$\beta C_{\mathrm{m}} \frac{\partial V_{\mathrm{m}}}{\partial t} - \nabla \cdot \boldsymbol{\sigma}_{\mathrm{i}} \nabla V_{\mathrm{m}} = \nabla \cdot \boldsymbol{\sigma}_{\mathrm{i}} \nabla \Phi_{\mathrm{e}} - \beta \left(I_{\mathrm{ion}} \left(V_{\mathrm{m}}, \boldsymbol{\eta} \right) - I_{\mathrm{s}} \right), \tag{A12}$$

retaining the quantities of primary interest, V_{m} and Φ_{e}, where only Φ_{e} is observable.

A.2 Monodomain Model

The monodomain model represents a simplified form of the bidomain model in favor of substantially reduced computational complexity. This form is derived from the bidomain model by assuming that the intra- and extracellular conductivity tensors are proportionally coupled, i.e. $\boldsymbol{\sigma}_{\mathrm{i}} = k \cdot \boldsymbol{\sigma}_{\mathrm{e}}$ [1, 2]. Consequently, the parabolic (A12) and elliptic (A11) portions of the bidomain equations are effectively decoupled and can be solved independently. This yields the monodomain equations, defined over the myocardial domain $\Omega_{\mathrm{H}} \subset \mathbb{R}^3$, given by

$$\beta C_{\mathrm{m}} \frac{\partial V_{\mathrm{m}}}{\partial t} = \nabla \cdot \left(\boldsymbol{\sigma}_{\mathrm{m}} \nabla V_{\mathrm{m}} \right) - \beta \left(I_{\mathrm{ion}} \left(V_{\mathrm{m}}, \boldsymbol{\eta} \right) - I_{\mathrm{s}} \right) \qquad \text{in } \Omega_{\mathrm{H}}, \tag{A13}$$

$$\frac{\partial \boldsymbol{\eta}}{\partial t} = f \left(V_{\mathrm{m}}, \boldsymbol{\eta} \right) \qquad \text{in } \Omega_{\mathrm{H}}, \tag{A14}$$

$$\boldsymbol{\sigma}_{\mathrm{m}} \nabla V_{\mathrm{m}} \cdot \mathbf{n}_{\mathrm{h}} = 0 \qquad \text{on } \partial\Omega_{\mathrm{H}}, \tag{A15}$$

with initial conditions $\boldsymbol{\eta}(t=0) = \boldsymbol{\eta}_0$ and $V_{\mathrm{m}}(t=0) = V_{\mathrm{m},0}$. Here, $\mathbf{n}_{\mathrm{h}} \in \mathbb{R}^3$ is the outward normal vector of Ω_{H} defined on the domain's boundary $\partial\Omega_{\mathrm{H}}$. The anisotropic monodomain conductivity tensor $\boldsymbol{\sigma}_{\mathrm{m}}$ is related to the bidomain intra- and extracellular conductivity tensors by means of the harmonic equation

$$\sigma_{m,\zeta} = \sigma_{\mathrm{i},\zeta} \left(\sigma_{\mathrm{i},\zeta} + \sigma_{\mathrm{e},\zeta} \right)^{-1} \sigma_{\mathrm{e},\zeta}, \tag{A16}$$

along the orthonormal eigenaxes $\zeta \in \{\mathbf{f}, \mathbf{s}, \mathbf{n}\}$. The parameters of the calibrated monodomain model employed in this study, shown in Table S1, were obtained using the standardized CEP calibration framework ForCEPSS [3]. The extracellular potential

Region	$v_{0,l}$ $\left[\frac{\mathrm{m}}{\mathrm{s}}\right]$	$v_{0,t}$ $\left[\frac{\mathrm{m}}{\mathrm{s}}\right]$	$\sigma_{e,l}$ $\left[\frac{\mathrm{S}}{\mathrm{m}}\right]$	$\sigma_{e,t}$ $\left[\frac{\mathrm{S}}{\mathrm{m}}\right]$	$\sigma_{i,l}$ $\left[\frac{\mathrm{S}}{\mathrm{m}}\right]$	$\sigma_{i,t}$ $\left[\frac{\mathrm{S}}{\mathrm{m}}\right]$	$\sigma_{m,l}$ $\left[\frac{\mathrm{S}}{\mathrm{m}}\right]$	$\sigma_{m,t}$ $\left[\frac{\mathrm{S}}{\mathrm{m}}\right]$
Healthy	0.8533	0.4732	1.1784	1.0589	0.3281	0.0852	0.2566	0.0789
Border-Zone	0.5909	0.1072	1.1082	0.1796	0.3085	0.0145	0.2413	0.0134

Table S1 Parametrization of the reaction-diffusion (RD) model. Reference RD model parameters are shown for electrically viable normal and border-zone tissues with healthy and impaired EP. Given prescribed planar wave front conduction velocitys (CVs) in longitudinal $l \in \{\mathbf{f}\}$ and transverse $t \in \{\mathbf{s}, \mathbf{n}\}$ fiber orientations for each tissue type, the reference RD model was calibrated for a target spatial discretization of 250 µm, a cell surface-to-volume ratio of $\beta = 0.14\,\left[\mu\mathrm{m}^{-1}\right]$ and a cell membrane capacitance of $C_\mathrm{m} = 0.01\,\left[\mu\mathrm{F\,cm}^{-2}\right]$. The prescribed CVs, C_m and β were carried over from [3, 8].

field required to produce electro-cardiograms (ECGs) can be recovered from the cardiac source distribution V_m by either solving (A11), referred to as pseudo-bidomain model, or, more efficiently when extracellular potentials are evaluated only at discrete sites $\mathbf{x}_i$ to obtain $\Phi_\mathrm{e}(\mathbf{x}_i, t)$ potentials of the ECG, by employing the lead-field method [4] (see A.5).

A.3 Parameterization of the reference RD model

All simulations were carried out using the pseudo-bidomain model where the spatio-temporal evolution of the cardiac sources are given by the monodomain model as described in A.2. The parameters of the reference were calibrated to match the conduction velocitys (CVs) of the electrophysiology (EP) model of human ventricles with infarcted scar and border-zone tissue [5]. Cell and tissue EP properties were set to characterize normal ventricular tissue exhibiting healthy EP, and electrically viable border-zone tissue adjacent to infarct scars with impaired EP. Cellular dynamics were represented by myocytes of epicardial phenotypes according to the ten Tusscher-Noble-Noble-Panfilov (TNNP) model [6]. For border-zone tissue, the peak sodium current, I_Na, L-type calcium current, I_Cal, and the potassium currents, I_Kr and I_Ks, were reduced by 62%, 69%, 70% and 80%, respectively, resulting in a prolonged action potential duration (APD) duration, a decreased upstroke velocity and a decreased action potential (AP) amplitude. As the parameter set reported in [5] lead to discretization artifacts that may cause stochastic behavior [7], the reference RD model required calibration to yield prescribed target CVs for each viable tissue type. The RD model was calibrated at a high spatial resolution of 250 µm using ForCEPSS [3] to obtain converged model parameters, summarized in Table S1.

A.4 CV of planar wave front propagation in RD models

The CV of a propagating planar wave front in the full-order bidomain model is governed by the axial conductivities, $\boldsymbol{\sigma}_\mathrm{i}$ and $\boldsymbol{\sigma}_\mathrm{e}$, the excitable membrane surface per volume, β, and the excitability of cells governed by the availability of sodium currents encoded in I_ion. For a planar wave front propagating uniformly along a direction ζ the

Region	$\sigma_{m,l}$ $\left[\frac{\mathrm{S}}{\mathrm{m}}\right]$	$\sigma_{m,t}$ $\left[\frac{\mathrm{S}}{\mathrm{m}}\right]$	$v_{0,l}$ $\left[\frac{\mathrm{m}}{\mathrm{s}}\right]$	$v_{0,t}$ $\left[\frac{\mathrm{m}}{\mathrm{s}}\right]$	ρ_l $\left[\mathrm{m\,s^{-1}}\sqrt{\mathrm{m\,S^{-1}}}\right]$	ρ_t $\left[\mathrm{m\,s^{-1}}\sqrt{\mathrm{m\,S^{-1}}}\right]$
Healthy	0.2724	0.0835	0.8533	0.4732	1.6348	1.6378
Border-Zone	0.1213	0.0037	0.5909	0.1072	1.6965	1.7691

Table S2 Proportionality between CV and conductivity in RD models. The measurements of the proportional relationship between CV and the conductivity in RD models are shown. The RD model in Sec. A.3 was calibrated with ForCEPSS [3] to match the prescribed CVs on a fine 250 µm grid in longitudinal $l \in \{\mathbf{f}\}$ and transverse $t \in \{\mathbf{s}, \mathbf{n}\}$ fiber orientations of electrically viable normal and border-zone tissues. The proportional constants, ρ_ζ were determined for each eigenaxis $\zeta \in \{l, t\}$ based on the relationship Eq. (A17) from the measured CVs and the harmonic mean conductivities of the calibrated RD model.

following relation between velocity and conductivity along ζ holds

$$v_{0,\zeta} = \rho_\zeta \sqrt{\sigma_{m,\zeta}} \tag{A17}$$

where ρ_ζ is a proportionality constant which depends on the tissue surface-to-volume ratio β and other factors related to cellular dynamics such as sodium channel conductivity which affect propagation velocity. Table S2 lists experimentally determined proportionality constants ρ_ζ for the RD model and target CVs given in Sec. A.3 calibrated for a fine target resolution of 250 µm.

A.5 Lead-field Recovery

Full physics fidelity electrograms (EGMs) and ECG signals matching those of the gold-standard bidomain model can be recovered from a given source distribution $V_\mathrm{m}(t, \mathbf{x})$ and at an arbitrary electrode location ℓ employing the lead field formulation as proposed in [4]. The lead field is computed as the potential field Z_ℓ in the torso resulting from a unit current injected at the electrode location ℓ and withdrawn at a fixed but arbitrary reference electrode located at $\mathbf{r}$ by

$$\nabla \cdot ((\boldsymbol{\sigma_i} + \boldsymbol{\sigma_e})\nabla Z_\ell) = I_\ell\,\delta_\ell - I_\mathbf{r}\,\delta_\mathbf{r}$$

where $||I_\ell|| = ||I_\mathbf{r}|| = 1$ are unitary currents with $I_\ell - I_\mathbf{r} = 0$, and $\delta_\mathbf{y}$ is a shifted Dirac's delta function, i.e. $\delta_\mathbf{y}(\mathbf{x}) = \delta(\mathbf{y} - \mathbf{x})$. The lead field method thus yields the extracellular voltage $\phi_\mathbf{e}$, i.e. the EGMs or the ECGs trace, recorded at discrete locations ℓ corresponding to electrode placements relative to the location of the reference electrode at $\mathbf{r}$ by evaluating

$$\phi_\mathbf{e}(\ell) = \int_{\Omega_\mathrm{H}} \nabla Z_\ell \cdot \boldsymbol{\sigma_i}\,\nabla V_\mathrm{m}\,\mathrm{d}\mathbf{x} \tag{A18}$$

As the leadfields Z_ℓ can be pre-computed, the evaluation of EGMs and the ECG is computationally lightweight, and can be solved at negligible compute costs, as shown

previously [9–11]. It is important to note that this has no impact on the computation of the clinical observations ϕ_e for which only the shape of the AP is relevant.

Appendix B Algorithmic Description of the PIE Model

The three lightweight components of the Physics-Informed Eikonal (PIE) model are implemented within a cellular automaton framework that operates on spatio-temporally discretized domains. Thus, the simulation time frame $[t_{\text{start}}, t_{\text{end}}] \subset \mathbb{R}$ is discretized into $n \in \mathbb{N}$ time steps $\Delta t = (t_{\text{end}} - t_{\text{start}})/n$, such that the discrete simulation time is defined as $t = t_{\text{start}} + n \cdot \Delta t \in [t_{\text{start}}, t_{\text{end}}]$. Given a spatial target resolution $h \in \mathbb{R}_{>0}$, we denote by $\Omega_H = (\mathcal{T}_h, \mathcal{V}_h)$ the discretization of the myocardium Ω_H into N_T non-overlapping tetrahedral elements $T \in \mathcal{T}_h = \{T_i\}_{i=1}^{N_T}$ with N_V discrete vertices $\mathbf{v} \in \mathcal{V}_h = \{\mathbf{v}_i\}_{i=1}^{N_V}$. Furthermore, we define the element index set $\mathcal{I}_T = \{1, \ldots, N_T\}$, the vertex index set $\mathcal{I}_V = \{1, \ldots, N_V\}$, and the topology

$$\mathcal{N}_T(i) = \{j \in \mathcal{I}_T \mid \mathbf{v}_i \text{ is adjacent to } T_j \in \mathcal{T}_h\} \subset \mathcal{I}_T$$

as the index set of all elements that are adjacent to the vertex $\mathbf{v}_i \in \mathcal{V}_h$. Moreover, we define the topology

$$\mathcal{N}_V(i) = \{j \in \mathcal{I}_V \mid \exists\, k \in \mathcal{N}_T(j) : \mathbf{v}_i \text{ is adjacent to } T_k \in \mathcal{T}_h\} \subset \mathcal{I}_V$$

as the index set of all vertices that are connected to the vertex $\mathbf{v}_i \in \mathcal{V}_h$ by an element $T \in \mathcal{T}_h$. On the spatio-temporally discretized myocardium, cardiac fiber orientations are stored per tetrahedral element, while internal states of the cellular automaton are stored per vertex. This includes the cellular state variable $s \in \{\texttt{ACTIVATING}, \texttt{ACTIVE}, \texttt{RECOVERING}, \texttt{EXCITABLE}\}$, the local activation time, t_a, the action potential duration, APD_{90}, the AP cycle time, τ, and the transmembrane voltage, V_m. The algorithm of the PIE model is visualized by the flow chart in Fig. S1. After all states have been initialized as excitable region through an initialization phase, the cellular automaton controls finite state transitions based on the Finite State Machine (FSM) logic, alternating between an asynchronous depolarization and a synchronous repolarization phase in each time step, until the simulation concludes.

B.1 Depolarization phase

The depolarization phase of the cellular automaton handles state transitions related to stimulation and wave front expansion, which involves solving the Eikonal equation over a moving time horizon whilst considering physiological dependencies of the CV. To solve the Eikonal equation globally on the discretized domain Ω_H, the depolarization phase implements the Fast Iterative Method (FIM) [12]. This wave front expansion algorithm efficiently tracks `ACTIVATING` nodes via an active list to iteratively perform local and independent updates on the wave front arrival times t_a. Compared to other established algorithms (e.g. fast marching or sweeping methods), this design offers two

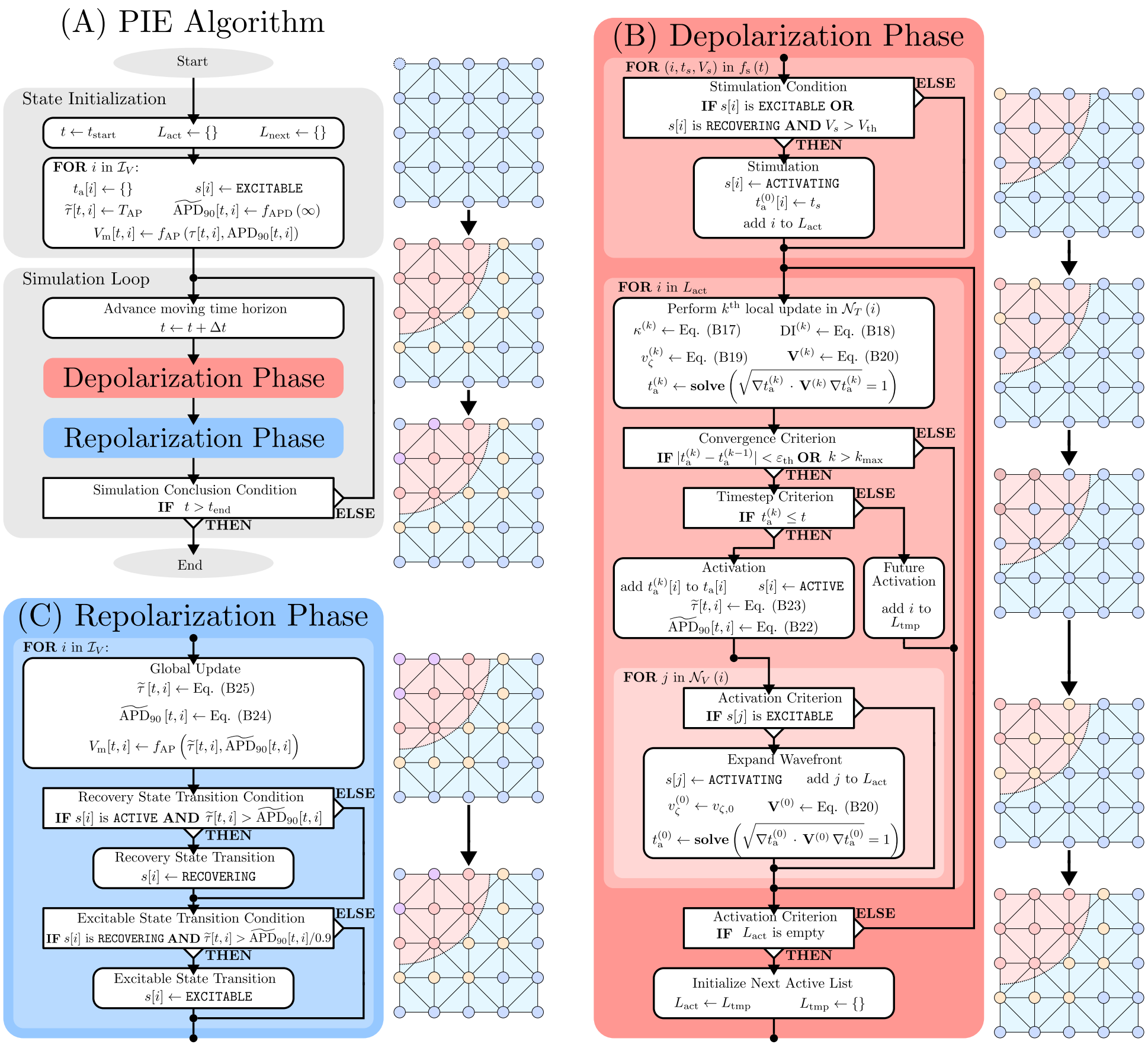

Fig. S1 Algorithmic description of the Physics-Informed Eikonal (PIE) model. The flow chart provides an algorithmic description of the PIE model as a cellular automaton (A), consisting of three phases. An initialization phase prepares nodal states in all myocardial regions accordingly to the simulation. (B) The depolarization phase covers state transitions related to stimulation and wave front propagation, i.e. (`EXCITABLE`, `RECOVERING`) → `ACTIVATING` and `ACTIVATING` → `ACTIVE`. This phase implements the Fast Iterative Method (FIM) that solves the conduction velocity (CV) dependent Eikonal solution of t_a over short moving time horizon Δt, enabling a reentrant wave propagation.(C) The repolarization phase implements the electrotonic diffusion mechanism, the transmembrane voltage recovery process and state transitions related to repolarization, i.e. `ACTIVE` → `RECOVERING` and `RECOVERING` → `EXCITABLE`.

important advantages: first, the independent updates can be computed in parallel, which enhances real-time capabilities on parallel computing architectures. Second, its iterative update approach allows to perform an intermediate CV update, depending on restitution and wave front curvature, by means of an iterative fixed-point scheme [13]. Let $k \in \mathbb{N}$ denote the index of the current fixed-point iteration performed on an `ACTIVATING` node i. In the initial $k = 0$ step, we assume planar wave front conditions,

that is $v_\zeta^{(0)} = v_{\zeta,0}$, to obtain an initial estimate of the activation time, $t_\mathrm{a}^{(0)}$, by solving the Eikonal equation (B24) once. In each fixed-point iteration, $k > 0$, the wave front curvature, $\kappa^{(k)}$, and the diastolic interval, $\mathrm{DI}^{(k)}$, are computed for each adjacent element, based on the activation time of the preceding iteration, $t_\mathrm{a}^{(k-1)}$, through

$$\kappa^{(k)} = \nabla \cdot \left(\frac{\boldsymbol{\sigma}_\mathrm{m}\, \mathbf{n}_\mathrm{WF}^{(k)}}{\sqrt{\mathbf{n}_\mathrm{WF}^{(k)} \cdot \boldsymbol{\sigma}_\mathrm{m}\, \mathbf{n}_\mathrm{WF}^{(k)}}} \right), \quad \text{with} \quad \mathbf{n}_\mathrm{WF}^{(k)} = \frac{\nabla t_\mathrm{a}^{(k-1)}}{\|\nabla t_\mathrm{a}^{(k-1)}\|}. \tag{B19}$$

The diastolic interval (DI) is projected into each element by

$$\mathrm{DI}^{(k)} = \frac{1}{4} \sum_{\mathbf{x}_j \in T} t_\mathrm{a}^{(k-1)}[j] - \widetilde{t}_\mathrm{r}[t - \Delta t, j], \tag{B20}$$

based on the diffused repolarization time, given by

$$\widetilde{t}_\mathrm{r}[t - \Delta t, i] = t - \widetilde{\tau}[t - \Delta t, i] + \widetilde{\mathrm{APD}}_{90}[t - \Delta t, i]. \tag{B21}$$

The CV is then updated accordingly in each adjacent element by evaluating the prescribed curvature and CV restitution functions

$$v_\zeta^{(k)} = v_{0,\zeta} \cdot f_\mathrm{CVC}\left(\kappa^{(k)}\right) \cdot f_{\mathrm{CVR},\zeta}\left(\mathrm{DI}^{(k)}\right), \tag{B22}$$

and computing the velocity tensor

$$\mathbf{V}^{(k)} = \left(v_f^{(k)}\right)^2 \cdot (\mathbf{f} \otimes \mathbf{f}) + \left(v_s^{(k)}\right)^2 \cdot (\mathbf{s} \otimes \mathbf{s}) + \left(v_n^{(k)}\right)^2 \cdot (\mathbf{n} \otimes \mathbf{n}), \tag{B23}$$

to obtain the activation time update $t_\mathrm{a}^{(k)}$ by solving the Eikonal equation, given by

$$\sqrt{\nabla t_\mathrm{a}^{(k)} \cdot \mathbf{V}^{(k)}\, \nabla t_\mathrm{a}^{(k)}} = 1 \quad \text{in} \quad \mathcal{N}_T(i), \tag{B24}$$

locally over all adjacent elements $\mathcal{N}_T(i)$. The fixed-point iteration process repeats until convergence, that is when the absolute difference between the current and previous activation times $|t_\mathrm{a}^{(k)} - t_\mathrm{a}^{(k-1)}|$ undercuts a prescribed threshold, ε_th, or a prescribed maximum number of iterations, k_max, is reached.

The depolarization phase combines this fixed-point iteration scheme and the FIM as follows. Upon entering the depolarization phase, a function $f_\mathrm{s}(t)$ may provide indices i of nodes to be stimulated within the current time step window $t_s \in [t - \Delta t, t]$. If a node provided by f_s is excitable, its initial activation time estimate $t_\mathrm{a}^{(0)}$ is initialized by the stimulation time t_s, its state s marked as `ACTIVATING` and its index i is added to the active list L_act. A node is excitable when its state is either `EXCITABLE` or the stimulation threshold V_th is exceeded on a `RECOVERING` node, leading to a premature activation. During each FIM iteration, all `ACTIVATING` wave front nodes within

the active list receive local fixed-point updates while their activation times have not converged. Converged nodes with an activation time outside of the current time step window are processed in a future time step, and thus moved to a temporary list L_{tmp}. Otherwise, the node becomes `ACTIVE`, is removed from the active list and its APD is predicted from the APD restitution curve based on the DI, though

$$\widetilde{\mathrm{APD}}_{90}\,[t-\Delta t, i] = \mathrm{APD}_{90}^{\mathrm{ref}}\,[i] = f_{\mathrm{APD}}\left(\mathrm{DI}^{(k)}\,[i]\right). \tag{B25}$$

As the node is activated, the cycle time is initialized by

$$\widetilde{\tau}\,[t-\Delta t, i] = t - \Delta t - t_{\mathrm{a}}^{(k)}\,[i] \tag{B26}$$

and the the wave front expansion is executed, which involves adding all excitable downwind 1-neighboring nodes to the active list. Qualified nodes receive a single activation time update and are set to `ACTIVATING`. The FIM performs iterative updates on nodes within the active list until all nodes within the moving time horizon have been updated. The depolarization phase concludes by initializing the active list for the next iteration with all nodes contained within the temporary list L_{tmp}.

B.2 Repolarization phase

The repolarization phase represents a synchronous update of every remaining nodal states that were not updated during the depolarization phase. This phase implements re-excitable state transitions, the voltage recovery and the effect of local electrotonic diffusion on state variables. The diffusion mechanism is applied within a local anisotropic region $E_{\mathbf{\Lambda},\delta}\,(\mathbf{x})$ onto the action potential duration $\widetilde{\mathrm{APD}}_{90}$ and the cycle time $\widetilde{\tau}$ of each node by computing

$$\widetilde{\mathrm{APD}}_{90}\,[t, i] = \sum_{T_k \in E_{\mathbf{\Lambda},\delta}(\mathbf{x}_i)} \frac{|T_k|}{4} \sum_{\mathbf{x}_j \in T_k} K\left(\Delta t, \mathbf{x}_i - \mathbf{c}_k\right) \widetilde{\mathrm{APD}}_{90}\,[t-\Delta t, j]\,, \tag{B27}$$

and

$$\widetilde{\tau}\,[t, i] = \sum_{T_k \in E_{\mathbf{\Lambda},\delta}(\mathbf{x}_i)} \frac{|T_k|}{4} \sum_{\mathbf{x}_j \in T_k} K\left(\Delta t, \mathbf{x}_i - \mathbf{c}_k\right) \min\left(\widetilde{\tau}\,[t-\Delta t, j] + \Delta t, T_{\mathrm{AP}}\right), \tag{B28}$$

using a heat kernel K as smoothing operator and the centers $\mathbf{c}_k$ of each tetrahedral element T_k. The transmembrane voltage at each point is recovered from the diffused action potential duration $\widetilde{\mathrm{APD}}_{90}$ and cycle time $\widetilde{\tau}$, given by

$$V_{\mathrm{m}}\,[t, i] = f_{\mathrm{AP}}\left(\widetilde{\tau}\,[t, i]\,, \widetilde{\mathrm{APD}}_{90}\,[t, i]\right), \tag{B29}$$

and is thus affected by diffusion. The lookup features bilinear interpolation between intermediate AP templates. By design of the calibration stage, the APD restitution

curve correlates with the AP template function, i.e. every APD from the restitution curve is uniquely linked to an AP within the template function. The repolarization phase concludes by applying a corresponding update of AP states using the FSM logic, given by

$$s[i] = f_{\mathrm{FSM}}\left(\widetilde{\tau}[t,i], \widetilde{\mathrm{APD}}_{90}[t,i]\right), \tag{B30}$$

which also depends on the diffused evaluation variables.

Appendix C Additional Results

C.1 Sources of Discrepancies between Models

Based on the results of the quantitative analysis of EP dynamics in Experiments A–C, as shown in Fig. 4, the sources of the discrepancies in CV and local repolarization time (LRT) between the RD and PIE models were further investigated. Observed errors in local activation time (LAT) were minor overall and thus not investigated in detail.

In the experiments A-C, the CV error maps showed a false estimation of CV by the PIE model in certain areas. The areas where discrepancies occur correspond to sites where wavefronts are initiated, collide, travel side by side at different speeds or terminate at a sealed end. These sites are represented by points below and above the gray identity line in the outlier plots from Fig. 4. In the RD model, these areas experience a diffusion induced source-sink imbalance, which accelerates or decelerates the wavefront. In the PIE model, the diffusion-driven wave propagation is modeled by the Eikonal formulation where diffusion-induced influences on propagation velocity are only considered in the presence of wavefront curvature. This deviation from the RD behavior explains the observed discrepancies in CV, most noticeable during the diffusion benchmark where a difference in the drag effect between the planar wavefronts of healthy and border-zone tissue was caused, displayed in Fig. S2A.

Lesser errors in LRTs were also observed at the sites of wavefront initiation, collision and the termination at the sealed ends, but were particularly large along the interface between healthy and border-zone regions in Experiment C. The reason for this large deviation is related to how diffusion is approximated by the PIE model during repolarization. Unlike the RD model where diffusion acts on the transmembrane voltage and indirectly affects LRTs, the PIE model locally applies diffusion to the relative cycle time, τ, and the APD to influence LRTs directly. These modeling differences lead to an overemphasized diffusion effect on APD near the interface between regions during the PIE simulation, as seen in Fig. S2B. This is confirmed by the relatively small discrepancies in LRT observed in the experiments A and B where diffusion plays a less dominant role.

C.2 Functional Reentry - Sensitivity

The sensitivity and variability of the reference RD and the PIE model during the functional reentry experiment was further investigated. For this purpose, the experiment was repeated for a set of perturbed initial conditions. The peak sodium current I_{Na} of the calibrated reference RD model was adjusted between -10% and $+10\%$ to cause a slight perturbation of the CV, while the PIE model was recalibrated to match

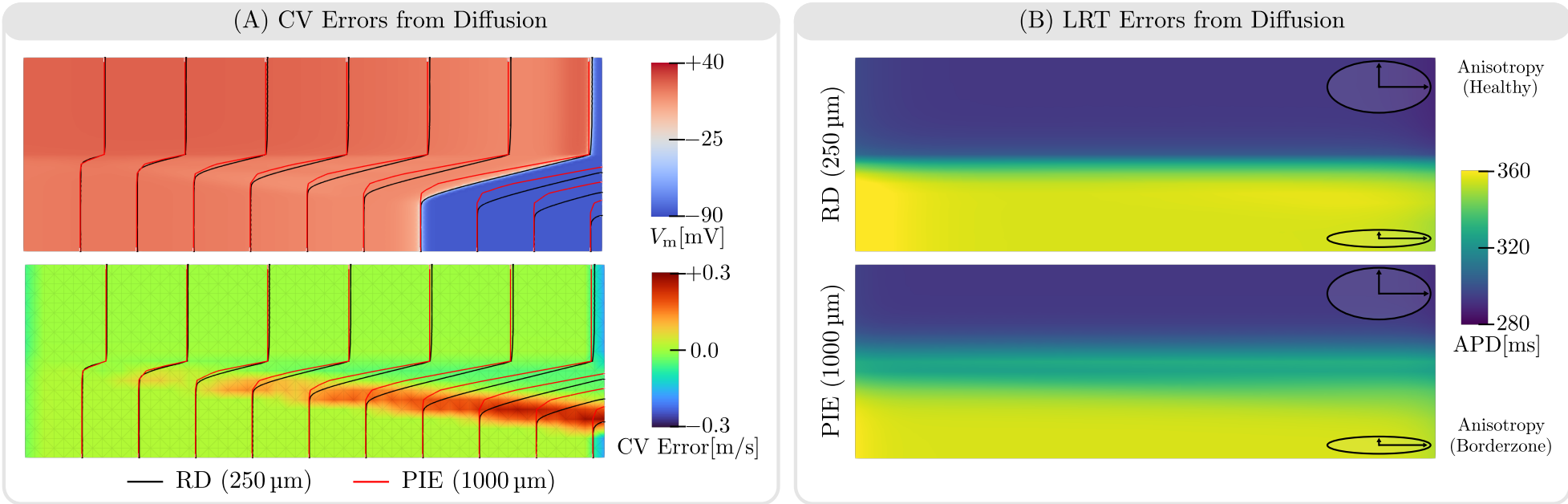

Fig. S2 Sources of discrepancies during the diffusion benchmark. (A) A comparison of the wavefront evolution between the reaction-diffusion (RD) and the Physics-Informed Eikonal (PIE) model based on local activation time (LAT) iso-surfaces, spaced in 10 ms intervals and visualized on top of the transmembrane voltage map, V_m, (top) and the conduction velocity (CV) error map (bottom). The majority of CV errors arise from the misaligned wavefront that is formed by the drag effect between the planar wavefronts of healthy and border-zone tissue. The misalignment of this wavefront causes an earlier activation in the PIE model along the propagation direction of the drag effect, leading to an overestimation of CVs. (B) A comparison of action potential duration (APD) maps between the RD and the PIE models under diffusion. Noticeable deviations due to modeling differences occur primarily near the interface of both regions where the diffusion effect acts the most.

the same EP configuration. The resulting transmembrane voltage maps generated by both models were qualitatively compared over the 5 s simulation window in Fig. S3.

Under perturbation of initial conditions, the PIE model experiences near identical variational behavior as the RD model during the first quarter of the simulation. From 1850 ms onwards, the activation pattern produced by the PIE model diverges moderately with time from the RD model in all variations, which is most noticeable when the peak sodium current is reduced by −10% (left row). The location of the phase singularity is noticeably more affected by the variation in CV in the PIE model, while the location remains mostly stable in the RD model.

As diffusion plays a significant role during the formation and maintenance of functional reentries, the observed discrepancies between models under varying initial conditions can be primarily attributed to their difference in implementation of the diffusion mechanic. The PIE model approximates the global diffusion of currents in RD models by applying local diffusion on the cycle time τ and APD, as described in the Methods section. While this approach reduced computational complexity, the exact diffusion behavior of RD models was not reproduced, in particular at the rotor core. Practically however, the divergence in variational solutions occurs within reasonable limits of uncertainty, considering the chaotic nature of functional reentries and long simulation period of 5 s. Overall, this experiment demonstrates that the PIE model is able to produce comparable perturbation behavior seen in RD models.

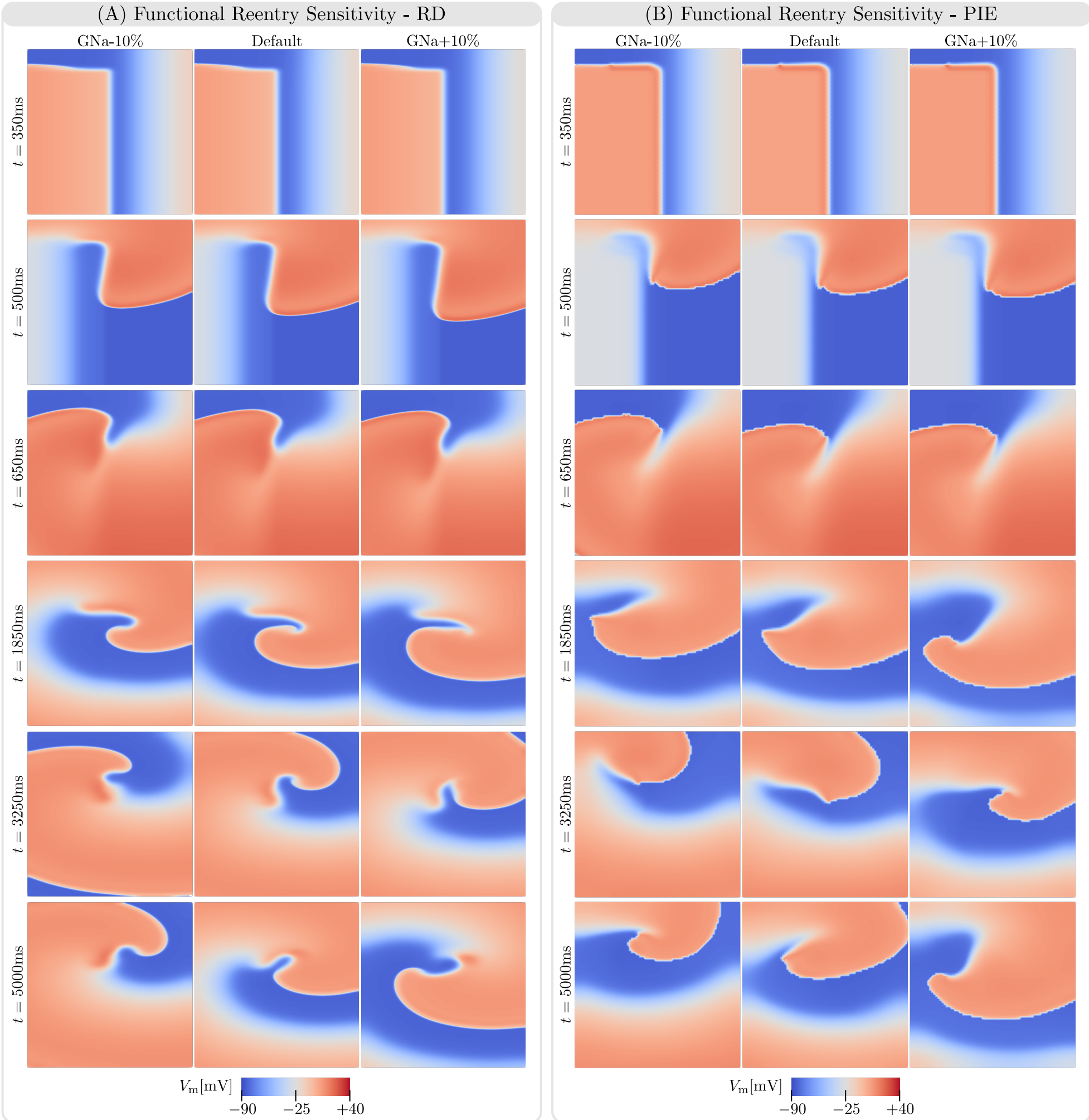

Fig. S3 Functional reentry sensitivity. Activation, repolarization and reentry pattern comparison between transmembrane voltage maps generated by (A) the reaction-diffusion (RD) and (B) the Physics-Informed Eikonal (PIE) model under perturbed settings during the functional reentry experiment. In both models, the maximal conductance G_{Na} for I_{Na} was altered by ±10% and reentry was sustained throughout the entire 5 second long simulation.

C.3 Whole-Heart - Impact of Spatial Resampling

The impact of spatial resampling on ECG fidelity during the whole-heart experiment was further investigated for the PIE model. For this purpose, the fine 250 µm mesh of the RD model was downsampled to three coarse resolutions, 1000 µm, 2000 µm and 3000 µm using MeshTool [14]. In particular, two resampling strategies were evaluated: (i) unconstrained uniform coarsening (ii) constrained resampling that preserves the topology of scar and border-zone tissue to maintain the reentry path length (see Fig. S4). The ECGs computed by the PIE model for each case, given in Fig. S5, were

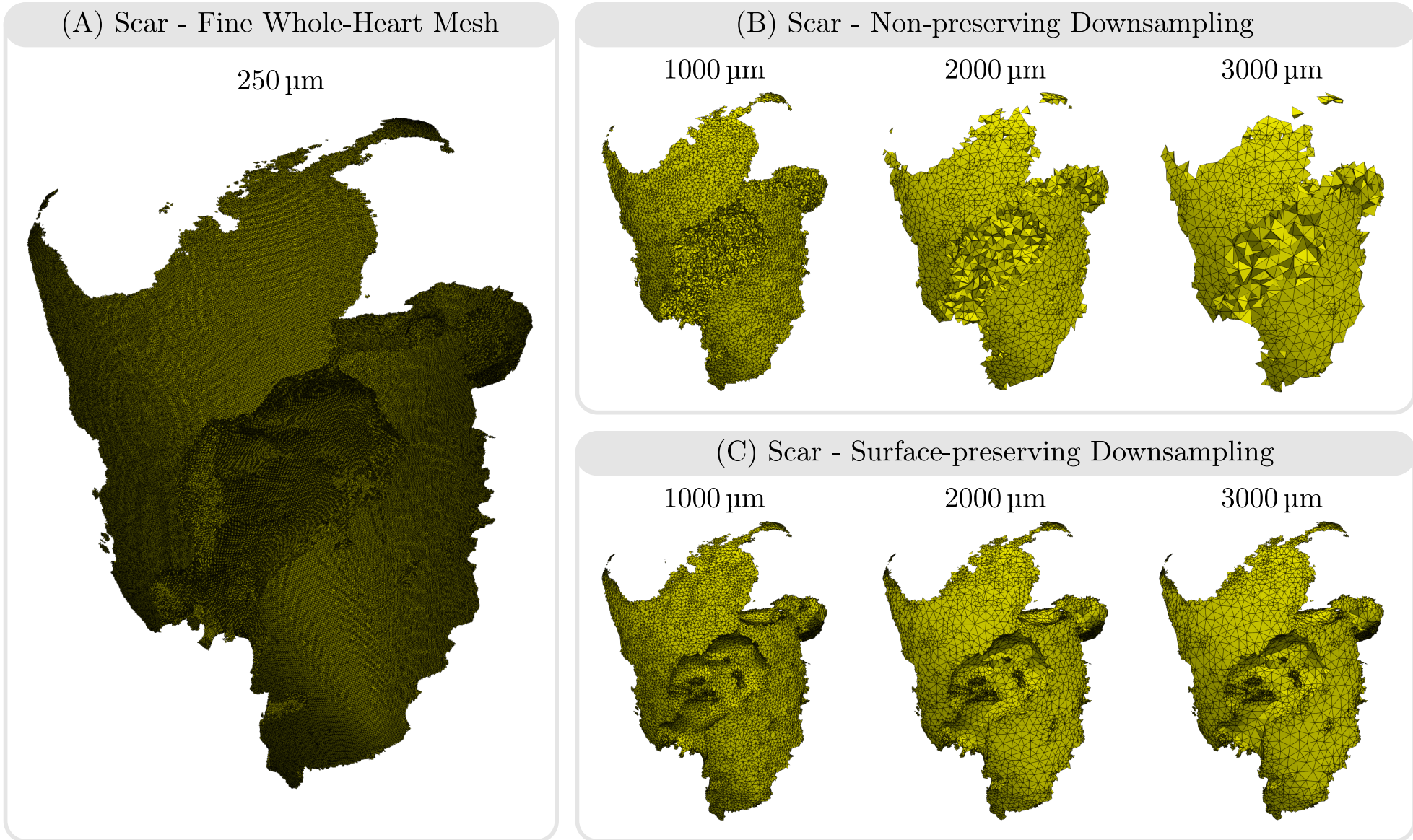

Fig. S4 Mesh downsampling comparison. Comparison of scar topology between different downsampling strategies employed in the whole-heart experiment. The fine 250 µm mesh of the reaction-diffusion (RD) model (A) was spatially downsampled without preserving (B) and with preserving (C) the fine surface details of the scar and surrounding border-zone. Each downsampling was successively performed using MeshTool [14] where surface correlation was set to 5% (B) and 95% (C) to control surface preservation.

quantitatively compared with the same metrics used in experiment (F), the dynamic time warping (DTW), the Pearson correlation coefficient (PCC) and the R-R interval. The quantitative results for each resolution and resampling strategy are reported in Table S3.

A comparison of ventricular tachycardia (VT) signatures generated from the unconstrained coarsened meshes (Fig. S5, top) shows clear ECG deterioration at 2000 µm due to temporal decoherence, also evident by the lower PCCs and increased R-R intervals. At 3000 µm, reentry could not be sustained to the end of the simulation since propagation was canceled at around 1000 ms. In contrast, VT was sustained at each resolution when the topology of scar and border-zone tissues have been preserved during resampling (Fig. S5, bottom). Identical R-R interval measured across resolutions indicate that temporal coherence was maintained. While DTW errors slightly increased with coarser mesh resolution, the measured PCCs remained within variance. Overall, a high correlation between ECGs of the RD and the PIE model was reported across mesh resolutions when the topology of scar and border-zone tissue was preserved.

It was shown that the high sensitivity experienced by reentrant activity can be a severe source of discrepancy on ECG fidelity, independent of the used model. Neglecting structural preservation during downsampling causes degradation of ECG fidelity or even cancellation of reentrant propagation upwards of 1000 µm due to alteration of reentry path length. Preserving local topological features at an increased resolution

Metric	Δx[µm]	Pres.	I	II	III	maVR	aVL	aVF	V1	V2	V3	V4	V5	V6	MEAN ± STD	Sust.
DTW [mV]	1000	×	0.652	1.606	0.706	0.883	**0.459**	0.913	1.281	**2.967**	0.973	0.999	1.337	1.912	1.224 ± 0.657	✓
	2000	×	0.969	1.374	2.850	**0.786**	0.846	1.899	1.667	**4.497**	2.176	2.919	1.323	1.343	1.888 ± 1.039	✓
	3000	×	-	-	-	-	-	-	-	-	-	-	-	-	-	×
	1000	✓	0.608	1.556	0.601	0.797	**0.402**	0.772	1.566	**3.238**	0.937	0.876	1.474	1.679	1.209 ± 0.741	✓
	2000	✓	0.998	1.855	1.029	0.992	**0.844**	1.074	3.454	**4.352**	1.976	2.112	2.693	2.512	1.991 ± 1.064	✓
	3000	✓	1.292	2.515	1.207	1.740	**0.901**	1.338	**5.562**	4.189	2.533	3.046	3.581	3.066	2.581 ± 1.340	✓
PCC [-]	1000	×	0.778	**0.531**	0.767	0.769	0.773	0.749	0.728	0.593	0.629	0.657	0.745	**0.786**	0.709 ± 0.081	✓
	2000	×	0.109	**0.173**	0.122	**0.092**	0.116	0.132	0.113	0.140	0.128	0.111	0.099	0.107	0.120 ± 0.021	✓
	3000	×	-	-	-	-	-	-	-	-	-	-	-	-	-	×
	1000	✓	0.925	**0.707**	0.911	0.925	0.919	0.893	0.899	0.839	0.846	0.863	0.917	**0.932**	0.881 ± 0.061	✓
	2000	✓	**0.952**	**0.770**	0.946	0.942	0.950	0.933	0.944	0.897	0.909	0.912	0.938	0.928	0.918 ± 0.048	✓
	3000	✓	**0.931**	**0.676**	0.926	0.908	0.930	0.911	0.929	0.862	0.876	0.874	0.908	0.898	0.886 ± 0.067	✓
R-R [ms]	250	-	327	333	333	327	327	333	333	332	331	328	327	327	330 ± 2	✓
	1000	×	327	335	335	328	327	335	337	334	334	327	328	329	331 ± 3	✓
	2000	×	344	349	345	344	344	345	349	347	347	342	344	344	345 ± 2	✓
	3000	×	344	406	398	328	337	398	391	351	353	343	363	359	364 ± 25	×
	1000	✓	326	335	335	327	326	335	337	334	334	326	327	328	331 ± 4	✓
	2000	✓	327	335	335	327	326	335	337	334	334	325	326	327	331 ± 4	✓
	3000	✓	325	335	335	327	327	335	335	334	334	325	326	328	331 ± 4	✓

Table S3 Mesh influence on whole-heart electro-cardiogram (ECG) measurements. Extended quantitative comparison between ECG lead signals computed by the reference reaction-diffusion (RD) and the Physics-Informed Eikonal (PIE) model based on the non-normalized dynamic time warping (DTW) distance measure [15, 16], the Pearson correlation coefficient (PCC) [17, 18] and measured R-R intervals during the whole-heart experiment (F). Resampled meshes ranging from 1000 µm to 3000 µm are marked when the topology of the scar and the border-zone has been structurally preserved (Pres). Lower DTW distance measures indicate a close match in morphology between signals, while the PCC describes the correlation of signals between -1 (anti correlation) and 1 (correlation). Bold entries mark the best and worst DTW and PCC results per row. The R-R intervals were measured as the interval between consecutive R-peaks, from which mean and standard deviation were computed across all leads. Simulations are marked wether reentry was sustained to the end or not (Sust). The 3000 µm non-preserved case failed to produce meaningful DTW and PCC measures as the reentry was not sustained.

prevents derivations in reentry path length such that ECG fidelity can be maintained at coarse resolutions. In this case, the observed and measured differences in ECGs signals were minor between resolutions, demonstrating that the reentry path was maintained and that the EP behavior of the PIE model behaves correctly across resolutions. Considering that RD models require high resolution meshes to maintain accuracy, employing topologically-preserving mixed resolution meshes during PIE simulations represents an effective strategy to maintain ECG fidelity, as well as reducing computational load which was demonstrated in Supplementary Sec. C.4.

C.4 Whole-Heart - Computational Demands

The computational demands of simulating the whole-heart experiment with the reference RD and the PIE model were assessed quantitatively by measuring their required execution times for calibration, initialization, simulation and lead-field reconstruction, respectively. The reference RD simulation was conducted at an resolution of 250 µm on the Austrian Scientific Cluster – utilizing four compute nodes with a total number of 192 Central Processing Unit (CPU) cores. Each compute node was equipped with two 24-core Intel Xeon Platinum 8174 CPUs, 96GB of system memory and AlmaLinux 8.8 as operating system. The PIE simulations were carried out at 1000 µm, 2000 µm and 3000 µm resolution on an Ubuntu 22.04 LTS workstation system equipped with

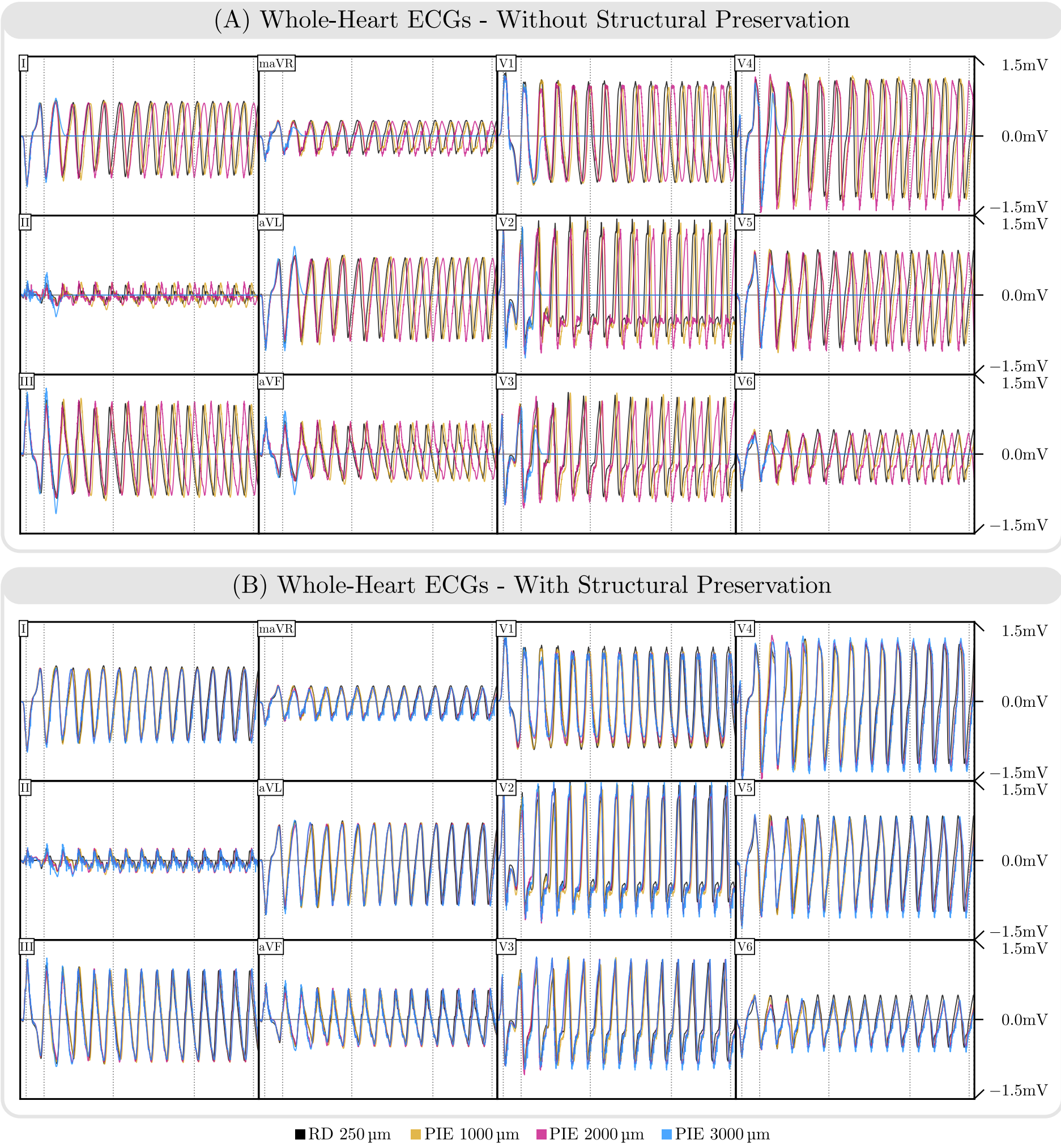

Fig. S5 Mesh influence on the whole-heart electro-cardiogram (ECG). Reconstructed 12-lead ECGs between the reaction-diffusion (RD) and Physics-Informed Eikonal (PIE) model using different spatial resampling strategies. Panel (A) shows the ECGs that were obtained without structural preservation of scar and border-zone tissues. Panel (B) shows resulting ECGs when the structure of scar and border-zone tissues was preserved. For each strategy, the fine 250 µm RD mesh was downsampled to resolutions of 1000 µm (golden), 2000 µm (magenta) and 3000 µm (blue). The resulting ECGs produced by the PIE model are compared to the ECG of the reference RD model (black) over the 5s simulation window.

32 cores of an AMD Threadripper PRO 5975WX CPU and 128GB of system memory. These meshes were employed during the investigation of spatial resampling strategies on ECG fidelity, as reported in Supplementary Sec. C.3.

The performance results are reported in Table S4. Calibration was executed within similar time frames since both methods utilized the same ForCEPSS [3] framework for each tissue type. The high initialization time required by the RD model stems from

Method	Resolution	CPUs	Nodes	Calib.	Init.	Sim. + LF	Sust.
RD	250 µm	192	42.5M	104 s	16 590 s	44 121 s	✓
PIE	1000 µm	32	2.8M	98 s	1 s	242 s + 74 s	✓
	2000 µm		1.9M			68 s + 32 s	✓
	3000 µm		0.7M			16 s + 13 s	×
PIE (pres.)	1000 µm	32	3.2M	98 s	1 s	221 s + 56 s	✓
	2000 µm		2.0M			68 s + 32 s	✓
	3000 µm		0.9M			34 s + 15 s	✓

Table S4 Run time comparison between models. Practical run time comparison between the PIE and the baseline RD model during simulation of the whole-heart experiment, which involves the time required for calibration (Calib.), initialization (Init.), simulation and lead-field reconstruction (Sim. + LF). For both models, calibration and initialization were carried out according to the ForCEPSS [3] standard. PIE simulations employ downsampled meshes ranging from 1000 µm to 3000 µm and are marked when the topology of the scar and border-zone has been preserved (pres.) and when reentry was maintained throughout the simulation (Sust.). During the reference RD simulation, the lead-field reconstruction was computed simultaneously due to memory limitations, while the PIE model applied the lead-field reconstruction as a post-processing step.

the procedure to reach steady-state for standardized EP simulation, as dictated by the ForCEPSS [3] standard. Acquiring steady-state involves nodal ionic model prepacing at a fixed cycle length, followed by simulating three consecutive beats in the entire whole-heart. In comparison, initialization and pre-pacing on the PIE model is computationally cheap since steady-state was already acquired by the calibration stage.

The RD model required ~17 hours in total to complete the 5 s long whole-heart simulation, while execution of the same simulation took only ~4 minutes with the PIE model at a resolution of 1000 µm, resulting in a speedup of ~275. Further reductions in spatial resolution reduced the execution time of the PIE model considerably. At a coarser resolution of 2000 µm, the execution time was further reduced by 3.5× to ~1 minute, corresponding to a speedup of ~822× towards the RD model. In case of the 3000 µm mesh, execution by the PIE model required only 35 s, reducing execution time again by 3.5× while reaching a speedup of ~1735× compared to the RD model.

Overall, the reported performance results demonstrate the computational advantage of the PIE model towards RD-based CEP models, which primarily emerges from the lifted spatial constraints. Near real-time execution of the PIE model was achieved on mixed resolution meshes where topological structures were preserved at locally sufficient resolution. Crucially, these results demonstrate that preserving topological structures at coarse but locally sufficient resolution is more efficient than enforcing a higher overall resolution since ECG fidelity was virtually unaffected (see Sec. C.3). Strategies to close the remaining gap towards true real-time execution include optimization of numerical solver settings and offloading parallel computations to Graphics Processing Unit (GPU) accelerators. The latter approach is particularly

promising, since the wavefront propagation and diffusion mechanism, the computationally most expensive components of the PIE model, greatly benefit from massive parallelization [12].

References

[1] Tung, L.: A Bi-Domain Model for Describing Ischemic Myocardial DC Potentials. Ph.D. thesis, Massachusetts Institute of Technology, Cambridge, MA, USA (1978). ndltd.org (oai:dspace.mit.edu:1721.1/16177)

[2] Bishop, M.J., Plank, G.: Bidomain ECG simulations using an augmented monodomain model for the cardiac source. IEEE Trans. Biomed. Eng. **58**, 2297–2307 (2011) https://doi.org/10.1109/TBME.2011.2148718

[3] Gsell, M.A.F., Neic, A., Bishop, M.J., Gillette, K., Prassl, A.J., Augustin, C.M., Vigmond, E.J., Plank, G.: ForCEPSS—A framework for cardiac electrophysiology simulations standardization. Comput. Methods Programs Biomed. **251**, 108189 (2024) https://doi.org/10.1016/j.cmpb.2024.108189

[4] Potse, M.: Scalable and Accurate ECG Simulation for Reaction-Diffusion Models of the Human Heart. Front. Physiol. **9** (2018) https://doi.org/10.3389/fphys.2018.00370

[5] Arevalo, H.J., Vadakkumpadan, F., Guallar, E., Jebb, A., Malamas, P., Wu, K.C., Trayanova, N.A.: Arrhythmia risk stratification of patients after myocardial infarction using personalized heart models. Nat. Commun. **7**, 11437 (2016) https://doi.org/10.1038/ncomms11437

[6] ten Tusscher, K.H., Panfilov, A.V.: Alternans and spiral breakup in a human ventricular tissue model. Am J Physiol Heart Circ Physiol. **291**, 1088–1100 (2006) https://doi.org/10.1152/ajpheart.00109.2006

[7] Bishop, M.J., Plank, G.: Stochastic virtual heart model predictions. Nat Cardiovasc Res **4**, 539–542 (2025) https://doi.org/10.1038/s44161-025-00641-1

[8] Neic, A., Campos, F.O., Prassl, A.J., Niederer, S.A., Bishop, M.J., Vigmond, E.J., Plank, G.: Efficient computation of electrograms and ECGs in human whole heart simulations using a reaction-eikonal model. J. Comput. Phys. **346**, 191–211 (2017) https://doi.org/10.1016/j.jcp.2017.06.020

[9] Zappon, E., Azzolin, L., Gsell, M.A.F., Thaler, F., Prassl, A.J., Arnold, R., Gillette, K., Kariman, M., Manninger, M., Scherr, D., *et al.*: An efficient end-to-end computational framework for the generation of ECG calibrated volumetric models of human atrial electrophysiology. Med. Img. Anal. **107**, 103822 (2025) https://doi.org/10.1016/j.media.2025.103822

[10] Gillette, K., Gsell, M.A.F., Prassl, A., Karabelas, E., Reiter, U., Reiter, G.,

Grandits, T., Stern, D., Urschler, M., Bayer, J., Augustin, C.M., Neic, A., Pock, T., Vigmond, E., Plank, G.: A Framework for the Generation of Digital Twins of Cardiac Electrophysiology from Clinical 12-leads ECGs. Med. Imag. Anal. **71**, 102080 (2021) https://doi.org/10.1016/j.media.2021.102080

[11] Pezzuto, S., Kal'avský, P., Potse, M., Prinzen, F.W., Auricchio, A., Krause, R.: Evaluation of a Rapid Anisotropic Model for ECG Simulation. Front. Physiol. **8** (2017) https://doi.org/10.3389/fphys.2017.00265

[12] Fu, Z., Kirby, R.M., Whitaker, R.T.: A fast iterative method for solving the eikonal equation on tetrahedral domains. SIAM J Sci Comput. **35**, 473–494 (2013) https://doi.org/10.1137/120881956

[13] Chinchapatnam, P.P., Rhode, K.S., King, A., Gao, G., Ma, Y., Schaeffter, T., Hawkes, D.J., Razavi, R.S., Hill, D.L.G., Arridge, S., Sermesant, M.: Anisotropic Wave Propagation and Apparent Conductivity Estimation in a Fast Electrophysiological Model: Application to XMR Interventional Imaging. In: MICCAI, pp. 575–583 (2007). https://doi.org/10.1007/978-3-540-75757-3_70

[14] Neic, A., Gsell, M.A.F., Karabelas, E., Prassl, A.J., Plank, G.: Automating image-based mesh generation and manipulation tasks in cardiac modeling workflows using Meshtool. SoftwareX **11**, 100454 (2020) https://doi.org/10.1016/j.softx.2020.100454

[15] Pearson, K.: VII. Note on regression and inheritance in the case of two parents. Proc. R. Soc. Lond. **58**, 240–242 (1895) https://doi.org/10.1098/rspl.1895.0041

[16] Zappon, E., Gsell, M.A.F., Gillette, K., Plank, G.: Quantifying anatomically-based in-silico electrocardiogram variability for cardiac digital twins. Comput. Biol. Med. **189**, 109930 (2025) https://doi.org/10.1016/j.compbiomed.2025.109930

[17] Sakoe, H., Chiba, S.: Dynamic programming algorithm optimization for spoken word recognition. IEEE Trans. Acoust. **26**, 43–49 (1978) https://doi.org/10.1109/TASSP.1978.1163055

[18] Mueen, A., Keogh, E.: Extracting Optimal Performance from Dynamic Time Warping. In: Proc. ACM SIGKDD Int. Conf., vol. 22, pp. 2129–2130 (2016). https://doi.org/10.1145/2939672.2945383
