# Supplementary material for: A physics-informed eikonal model for simulating arrhythmias in the human heart in real-time
Source: Nat Commun. 2026 Jul 29;17:9209. doi: 10.1038/s41467-026-76050-0 (PMC13522352; doi:10.1038/s41467-026-76050-0)
Supplement: Supplementary file 2 [file 41467_2026_76050_MOESM2_ESM.pdf]

nature portfolio

Corresponding author(s): Gernot Plank

Last updated by author(s): Jul 17, 2026

Reporting Summary

Nature Portfolio wishes to improve the reproducibility of the work that we publish. This form provides structure for consistency and transparency in reporting. For further information on Nature Portfolio policies, see our Editorial Policies and the Editorial Policy Checklist.

Statistics

For all statistical analyses, confirm that the following items are present in the figure legend, table legend, main text, or Methods section.

n/a	Confirmed	
☐	☒	The exact sample size (n) for each experimental group/condition, given as a discrete number and unit of measurement
☒	☐	A statement on whether measurements were taken from distinct samples or whether the same sample was measured repeatedly
☒	☐	The statistical test(s) used AND whether they are one- or two-sided<br>*Only common tests should be described solely by name; describe more complex techniques in the Methods section.*
☒	☐	A description of all covariates tested
☒	☐	A description of any assumptions or corrections, such as tests of normality and adjustment for multiple comparisons
☐	☒	A full description of the statistical parameters including central tendency (e.g. means) or other basic estimates (e.g. regression coefficient) AND variation (e.g. standard deviation) or associated estimates of uncertainty (e.g. confidence intervals)
☒	☐	For null hypothesis testing, the test statistic (e.g. F, t, r) with confidence intervals, effect sizes, degrees of freedom and P value noted<br>*Give P values as exact values whenever suitable.*
☒	☐	For Bayesian analysis, information on the choice of priors and Markov chain Monte Carlo settings
☒	☐	For hierarchical and complex designs, identification of the appropriate level for tests and full reporting of outcomes
☐	☒	Estimates of effect sizes (e.g. Cohen's d, Pearson's r), indicating how they were calculated

Our web collection on statistics for biologists contains articles on many of the points above.

Software and code

Policy information about availability of computer code

Data collection	All reference experiments using the RD model were simulated with the openCARP framework (version 18.0), which includes the software for the finite element solver, mesh generation, manipulation and the measurement of physiological features encoded in ForCEPSS. All codes for replication of RD simulations are freely available through https://opencarp.org/download/.
Data analysis	The code for the evaluation of the experiments is publicly available in the GitHub database under https://github.com/medunigraz/PIE-Model-Experiments and in the Zenodo database under accession code https://doi.org/10.5281/zenodo.17198150. The implementation of the PIE solver (version 1.0) requires proprietary libraries which cannot be publicly disclosed. Binary executables compiled for the major platforms (Linux, macOS or Windows) can be provided upon request within a complete Docker image for replicating the results of this study.

For manuscripts utilizing custom algorithms or software that are central to the research but not yet described in published literature, software must be made available to editors and reviewers. We strongly encourage code deposition in a community repository (e.g. GitHub). See the Nature Portfolio guidelines for submitting code & software for further information.

Data

Policy information about availability of data

All manuscripts must include a data availability statement. This statement should provide the following information, where applicable:

- Accession codes, unique identifiers, or web links for publicly available datasets
- A description of any restrictions on data availability
- For clinical datasets or third party data, please ensure that the statement adheres to our policy

The whole-heart model used in this study was carried over from a previous study which is publicly accessible under https://doi.org/10.1016/j.media.2021.102080. All experimental setups employed in this study and its supplementary material, the calibration data of the RD and the PIE model, the generated ECGs and rendered videos of each experiment are publicly available in the GitHub database under https://github.com/medunigraz/PIE-Model-Experiments. A complete dataset including the calibration data, the generated meshes, the anonymized human whole-heart model and respective lead-field data, computed simulation data and rendered videos of each experiment have been deposited in the Zenodo database under accession code https://doi.org/10.5281/zenodo.17198150.

Research involving human participants, their data, or biological material

Policy information about studies with human participants or human data. See also policy information about sex, gender (identity/presentation), and sexual orientation and race, ethnicity and racism.

Reporting on sex and gender	This study involved only computational modeling using a previously developed anatomical model and did not involve the recruitment of participants or the collection of new human data. Consequently, participant demographic characteristics, including race and ethnicity, were not relevant to this study.
Reporting on race, ethnicity, or other socially relevant groupings	This study involved only computational modeling using a previously developed anatomical model and did not involve the recruitment of participants or the collection of new human data. Consequently, participant demographic characteristics, including race and ethnicity, were not relevant to this study.
Population characteristics	This study involved only computational modeling using a previously developed anatomical model and did not involve the recruitment of participants or the collection of new human data. Consequently, participant demographic characteristics, including race and ethnicity, were not relevant to this study.
Recruitment	This study involved only computational modeling using a previously developed anatomical model and did not involve the recruitment of participants or the collection of new human data. Consequently, participant demographic characteristics, including race and ethnicity, were not relevant to this study.
Ethics oversight	The study complies with applicable research ethics requirements.

Note that full information on the approval of the study protocol must also be provided in the manuscript.

Field-specific reporting

Please select the one below that is the best fit for your research. If you are not sure, read the appropriate sections before making your selection.

☒ Life sciences ☐ Behavioural & social sciences ☐ Ecological, evolutionary & environmental sciences

For a reference copy of the document with all sections, see nature.com/documents/nr-reporting-summary-flat.pdf

Life sciences study design

All studies must disclose on these points even when the disclosure is negative.

Sample size	This study presents a computational methodology based on an anatomical model of a single male subject. As no biological or experimental samples were analyzed and no statistical comparisons were performed, no statistical methods were used to predetermine sample size.
Data exclusions	No data were excluded from the analyses and the experiments were not randomized.
Replication	The software for reproducing all experimental results is publicly available in the GitHub database under https://github.com/medunigraz/PIE-Model-Experiments and in the Zenodo database under accession code https://doi.org/10.5281/zenodo.17198150. All reference results can be reproduced with open source simulation software openCARP. For details we refer to the Data and Code Availability sections.
Randomization	No data were excluded from the analyses and the experiments were not randomized.
Blinding	This study presents a computational methodology based on an anatomical model of a single male subject. As no biological or experimental samples were analyzed and no statistical comparisons were performed, no statistical methods were used to predetermine sample size.

Reporting for specific materials, systems and methods

We require information from authors about some types of materials, experimental systems and methods used in many studies. Here, indicate whether each material, system or method listed is relevant to your study. If you are not sure if a list item applies to your research, read the appropriate section before selecting a response.

Materials & experimental systems

n/a	Involved in the study
☒	☐ Antibodies
☒	☐ Eukaryotic cell lines
☒	☐ Palaeontology and archaeology
☒	☐ Animals and other organisms
☒	☐ Clinical data
☒	☐ Dual use research of concern
☒	☐ Plants

Methods

n/a	Involved in the study
☒	☐ ChIP-seq
☒	☐ Flow cytometry
☒	☐ MRI-based neuroimaging

Plants

Seed stocks	Not relevant to our computational study.
Novel plant genotypes	Not relevant to our computational study.
Authentication	Not relevant to our computational study.
