# Supplementary material for: A physics-informed eikonal model for simulating arrhythmias in the human heart in real-time
Source: Nat Commun. 2026 Jul 29;17:9209. doi: 10.1038/s41467-026-76050-0 (PMC13522352; doi:10.1038/s41467-026-76050-0)
Supplement: Supplementary file 3 [file 41467_2026_76050_MOESM3_ESM.pdf]

nature portfolio

Peer Review File

A Physics-Informed Eikonal Model for Simulating Arrhythmias in the Human Heart in Real-Time

Corresponding Author: Professor Gernot Plank

This file contains all reviewer reports in order by version, followed by all author rebuttals in order by version.

Version 0:

Reviewer comments:

Reviewer #1

(Remarks to the Author)
This manuscript presents a physics-informed Eikonal (PIE) framework designed as a fast surrogate for reaction–diffusion (RD) cardiac electrophysiology models. The computational burden of physically-detailed computational models of cardiac electrophysiology has been an important long-lasting challenge in this field such that it is really difficult for systematic analysis on those models such as sensitivity analysis and uncertainty quantification. Authors developed a computational framework that coupled Eikonal equations with the finite state machine models to accelerate the simulation. The results show significant computational speed improvement. They provided evidence to demonstrate the accuracy of the model. The results are novel and promising but some concerns need to be addressed.

Concerns.

1. More calibration is needed. Appendix C Table C3 shows the validation error, however, a summary metrics may omit outliers or errors in some subsamples. It would be helpful for authors to show the validation with plots where x axis is the value of the RD (original complex) model, and y axis is the value of PIE (simplified) model. A good reference figures are Figure 1 in PMID 34665814. Additionally, more whole heart quantitative validation is needed to ensure the generalizability, for example ECG related metrics including QT interval RR interval etc. Also, I consider the validation is the foundation of all the results such that it is worthwhile to put in the first figure/tables in the manuscript rather than supplements.

2. Error accumulated over time. In Fig. 3, the ECG comparison shows that the first beat matches the RD reference most closely, while the discrepancy increases with subsequent beats. Why does this error accumulate over time, and how might it be mitigated? More importantly, how can we determine whether the accumulated error remains sufficiently small for the PIE model to be trusted as a surrogate for RD simulations? Is there a way—prior to running long simulations—to estimate over how many seconds the PIE predictions will remain accurate enough to faithfully replace the RD model?

3. It seems that the PIE strategy can be useful when the initial condition is limit cycle/equilibirium. Is there a strategy to generalize it into non-equlibrium dynamics. For example, the heart run for 1 min, 60Hz, then administer a certain drug, see how the heart reacts.

4. The model RD model is deterministic but should be chaotic particularly when it comes to modeling tachycardia/re-entry, will the PIE model also going to capture this chaotic behavior?

5. Will the model work with different diseased condition? For example, under the Ca overload, LQTS syndrome type 1 (IKs loss of function) or CPVT (RyR alteration).

6. Could PIE model be used for studies of channelopathies? For example, different LQTS1 mutations lead to distinct functional effects on IKs (encoded by KCNQ1), and clinically leading to Torsades de Pointes. Because each LQTS1 mutation produces a different degree of IKs loss-of-functon(PMID 21451124), my understanding is that PIE would currently require separate re-validation for each mutation, which may diminish the computational advantage. Would it be feasible to incorporate ion-channel kinetic parameters directly into PIE model? For instance, prior studies (PMID: 37705243) have mapped IKs-related parameters to APD using simple logistic models that relate channel conductance and kinetic properties to early afterdepolarization risk. It might also be possible to build models to map to CV restitution. If such parameter–

phenotype mappings could be embedded into the PIE/FSM framework, the approach might generalize across families of LQTS1 mutations (or other channelopathies), enabling accelerated whole-heart prediction across a spectrum of channel dysfunction.

(Remarks on code availability)

Reviewer #2

(Remarks to the Author)
This work introduces a Physics-Informed Eikonal (PIE) model implemented into a computational framework designed to reproduce key electrophysiological behaviors, including activation, repolarization, conduction velocity restitution, curvature effects and diffusion-mediated repolarization smoothing, as well as to simulate arrhythmias and clinical ECGs in near-real-time. This is promising and a great contribution to computational cardiology with potential for clinical application, addressing a critical bottleneck in cardiac digital twins: the current issue with high computational costs associated with full RD simulations.
I believe this manuscript is suitable for publication in Nature Communications; however, I suggest a few improvements below before it being accepted for publication (Minor Revisions).

Major Comments
1. Validation of reentry should use quantitative metrics beyond visual comparisons: The functional reentry experiment currently uses qualitative assessment and phase singularity tracking. Given the nonlinear sensitivities observed, perfect overlap is impossible, but this piece of validation would benefit from using some quantitative metrics. These could include a comparison of dominant frequency maps, trajectory metrics such as mean squared error and phase-field correlation over time. This would help assess whether the model could be used for mechanistic simulations. If quantitative metrics cannot be used, please provide a justification on how qualitative assessment is sufficient.

2. DTW and PCC are useful metrics to validate PIE results against ECG, but these may be insufficient. The ECG comparison presented in Table 1 shows good results overall, but some leads (e.g., V4–V6, mean PCC~0.71) show moderate discrepancies. No clinical threshold is provided to indicate what level of mismatch is acceptable for clinical tasks including guidance for ablation procedures. Additional metrics such as QRST integral differences would be beneficial for this piece of validation.

3. The manuscript repeatedly claims the PIE model provides “full physical fidelity”. This is an overstatement, since it still has some approximations. For instance: 1) Ionic currents are not resolved; 2) Diffusion is only locally approximated. These claims should then be lessened. The proposed PIE model offers high fidelity, but not full mechanistic fidelity.

4. There are several questions around model scalability and application in real-time. While speedups of up to 400x are very worthy, the practical real-time usage of the framework depends on a few computational aspects, including mesh resolution constraints, solver time refinement, and parallelization efficiency across CPU/GPU hardware.
- What is the minimal resolution to reproduce clinical ECGs in a reliable manner?
- How does PIE performance scale on GPUs, which are now standard for clinical modeling?

5. The authors acknowledge limitations but should include explicit text about scenarios where PIE should not be used, e.g., early after-depolarizations or drug-induced ionic changes.

(Remarks on code availability)
The code appears to be reasonably well-organized, though the level of reproducibility varies across different components. The repository does include a README file, and instructions are clear on the GitHub page. I was able to install the required packages and reproduce the experimental results.

Version 1:

Reviewer comments:

Reviewer #1

(Remarks to the Author)
The authors have addressed nearly all of my comments clearly and thoroughly, and I am satisfied with the revisions overall.

I have one remaining point regarding the response to my major concern 1a. The inclusion of the outlier plots in Figure 4 significantly improves the validation. However, the errors observed for certain experiments, particularly for CV under diffusion-dominated conditions and LRT in these settings, appear relatively large. Can authors provide additional clarification on the reasons/causes for these discrepancies.

(Remarks on code availability)

Reviewer #2

(Remarks to the Author)
The authors have made substantial revisions to the manuscript and have satisfactorily addressed all of my comments and concerns. I have no further comments and recommend the manuscript for publication.

(Remarks on code availability)
The code is appropriate and reusable, with clear instructions.

Version 2:

Reviewer comments:

Reviewer #1

(Remarks to the Author)
Authors has addressed the remaining question. I have no further questions or comments.

(Remarks on code availability)

Open Access This Peer Review File is licensed under a Creative Commons Attribution 4.0 International License, which permits use, sharing, adaptation, distribution and reproduction in any medium or format, as long as you give appropriate credit to the original author(s) and the source, provide a link to the Creative Commons license, and indicate if changes were made.
In cases where reviewers are anonymous, credit should be given to 'Anonymous Referee' and the source.
The images or other third party material in this Peer Review File are included in the article’s Creative Commons license, unless indicated otherwise in a credit line to the material. If material is not included in the article’s Creative Commons license and your intended use is not permitted by statutory regulation or exceeds the permitted use, you will need to obtain permission directly from the copyright holder.
To view a copy of this license, visit https://creativecommons.org/licenses/by/4.0/

Response to the Referee Reports for: A Physics-Informed Eikonal Model for Simulating Arrhythmias in the Human Heart in Real-Time

Thomas G. Schrotter[1,3], Matthias A. F. Gsell[1], Elena Zappon[1], Christoph M. Augustin[1,2,3], Aurel Neic[3], Simone Pezzuto[4,5], Gernot Plank[1,2,3,*,#]

[1]Gottfried Schatz Research Center, Division of Medical Physics and Biophysics, Medical University of Graz, Neue Stiftingtalstraße 6, 8010, Graz, Austria
[2]BioTechMed-Graz, Mozartgasse 12/II, 8010, Graz, Austria
[3]NumeriCor GmbH, Conrad-von-Hötzendorfstraße 26/2, 8010, Graz, Austria
[4]Department of Mathematics, University of Trento, Via Sommarive 14, 38123, Trento, Italy
[5]Euler Institute, Università della Svizzera italiana, Via Buffi 13, 6900, Lugano, Switzerland
[*]Corresponding author: gernot.plank@medunigraz.at

We, the authors, would like to thank the Reviewers for their time invested in reading our manuscript and for providing a helpful set of reviews. All of the suggestions and comments from the Reviewers have been thoroughly considered and, where appropriate and possible, we have changed the manuscript accordingly. In the sections below, we have responded individually to every concern and comment raised by the Reviewers.
For the sake of tracking changes implemented in our revision, both in the manuscript and the response, changes corresponding to concerns or comments of Reviewer 1 have been highlighted in red and changes of Reviewer 2 have been highlighted in blue. Changes independent of Reviewer comments have been highlighted in purple.

Reviewer's Responses to Questions

General changes

Reviewer 1

This manuscript presents a physics-informed Eikonal (PIE) framework designed as a fast surrogate for reaction–diffusion (RD) cardiac electrophysiology models. The computational burden of physically-detailed computational models of cardiac electrophysiology has been an important long-lasting challenge in this field such that it is really difficult for systematic analysis on those models such as sensitivity analysis and uncertainty quantification. Authors developed a computational framework that coupled Eikonal equations with the finite state machine models to accelerate the simulation. The results show significant computational

speed improvement. They provided evidence to demonstrate the accuracy of the model. The results are novel and promising but some concerns need to be addressed.
We thank the reviewer for their positive assessment and constructive comments.

Major Concerns:

1.a More calibration is needed. Appendix C Table C3 shows the validation error, however, a summary metrics may omit outliers or errors in some subsamples. It would be helpful for authors to show the validation with plots where x axis is the value of the RD (original complex) model, and y axis is the value of PIE (simplified) model. A good reference figures are Figure 1 in PMID 34665814.

We thank the Reviewer for their insightful comments and suggestions.

We followed the reviewer's suggestion and added a comprehensive quantitative validation to the Results in the main text (see Secs. Experiment (A) - p.10–11, l.445 – 475, Experiment (B) - p.11–12, l.477 – 511 and Experiment (C) - p.12, l.513 – 537) including outlier plots as used in Fig. 1 in PMID 34665814 (see revised Fig. 4). The new plots show consistent results between the RD and PIE solvers, specifically for LATs which agree very well in all calibration experiments. More notable outliers appeared in CV and LRT in specific areas such as sites of wavefront collisions, near stimulation sites and domain boundaries, respectively. Overall, the number of outliers between the models was small.

1.b Additionally, more whole heart quantitative validation is needed to ensure the generalizability, for example ECG related metrics including QT interval RR interval etc.

As a quantitative validation, we further added R-R interval measurements indicative of the VT cycle length (see revised Tab. 1 and C3), and corresponding quantitative comments in Secs. Experiment (F) - p.18, l.816. Only minor differences in R-R interval variability across the computed heartbeats were observed. QT measurements were not conducted as these are only meaningful during a normal rhythm where depolarization reflected in a QRS complex and repolarization associated to a T-wave are largely disjoint events. As activation and repolarization are present at the same time during VT, their respective contributions overlap in the ECG and cannot be discriminated.

1.c Also, I consider the validation is the foundation of all the results such that it is worthwhile to put in the first figure/tables in the manuscript rather than supplements.

We followed the Reviewer's advice and moved figures related to the validation analyses (before Fig. C2-4) to the main body of the manuscript (revised Fig. 2-4). The contents of former Tab. C3 have been incorporated into the revised Fig. 4.

2. Error accumulated over time. In Fig. 3, the ECG comparison shows that the first beat matches the RD reference most closely, while the discrepancy increases with subsequent beats. Why does this error accumulate over time, and how might it be

mitigated?
More importantly, how can we determine whether the accumulated error remains sufficiently small for the PIE model to be trusted as a surrogate for RD simulations?

We thank the Reviewer for raising this important issue which led to a substantial improvement of our results. Investigating this issue we identified the following key factors:

- **Differences in reentrant path length induced by mesh resolution.** The RD and PIE model are solved on meshes with different spatial resolutions. The coarser PIE mesh was derived from the higher resolution RD mesh by downsampling without preserving domain interfaces between scar, border zone and healthy tissue. This altered the topology of scar and border zones, associated with a change in the effective path length of the VT circuit.
  We investigated this effect by applying two downsampling strategies: (i) a boundary-preserving approach where scar and border zone geometry remained unaltered, and (ii) the previously used global coarsening approach that does not explicitly consider domain interfaces.
  Both qualitative and quantitative ECG comparisons demonstrate that error accumulation over time disappears for the boundary-preserving approach, yielding a near-perfect temporal alignment of RD and PIE ECG. As the obtained improvements in matching are significant, we now show in the main text the results using the boundary-preserving approach (refer to revised Fig. 7, and Secs. Experiment (F) - p.17–18, l.765–822) and discuss such results in the Discussion section (see p.22, l.969–995). For a detailed report on the effects of boundary-preservation versus global coarsening we refer to the Supplementary Sec. C.2.
- **Differences due to restitution behavior.** For calibration of the PIE model, APD restitution was measured using an S1-S2 protocol and CV restitution using a dynamic protocol. The dynamic protocol describes restitution for a constant cycle length, and S1-S2 for the immediate fast response after a change in DI following a limit cycle at the preceding S1. The following slow accommodation after an S2 is described by a so-called constant cycle restitution. These behaviors are characterized by a restitution portrait protocol according to Kalb et al. [1].
  As the sequence of DIs seen by cells in the VT simulation differs from those DIs seen during restitution measurements, discrepancies in APD and CV may arise. However, as our new results show, these are very minor. The observed discrepancy was clearly dictated by the difference in VT circuit path length due to non-preservation of scar and border zone boundaries.
  In future work, further advances to better represent restitution behaviors in the PIE solver will be investigated that better capture the memory effects featured by models of cellular dynamics. This can be achieved by a more comprehensive encoding of the restitution landscape within the finite state machine beyond a single restitution curve. We included a comment on the effect of the protocol in the calibration phase in the Discussion section (see p.25, l.1123–1148).

3. Is there a way—prior to running long simulations—to estimate over how many seconds the PIE predictions will remain accurate enough to faithfully replace the RD model?

The reviewer raises an interesting question. In principle, from a bound of the local error in CV, the duration until CV errors have accumulated such that the divergence in wave front arrival times increases beyond a prescribed tolerance can be estimated for specific conditions, such as the VT experiment in our study. However, a general *a priori* estimate of the error accumulation over time for the PIE solver is not possible. In fact, this is also not feasible for the RD model as any change in mesh resolution or numerical setting will lead to divergence over time. Due to dependency on numerous factors that cannot be quantified, an *a priori* estimate is also not possible.

4. It seems that the PIE strategy can be useful when the initial condition is limit cycle/equilibirium. Is there a strategy to generalize it into non-equilibrium dynamics. For example, the heart run for 1 min, 60Hz, then administer a certain drug, see how the heart reacts.

As the Reviewer correctly points out, the PIE strategy employs calibration under limit-cycle conditions. In principle, the PIE solver can be extended to accommodate such a scenario where physiological properties evolve over time. However, this would require a more complex state machine or a repetitive re-calibration which may reduce the computational advantages and increase complexity of the PIE solver. It is worth noting here that most current RD models, such as openCARP, also do not support time-varying parameters. Such simulations would require checkpointing, re-initialization and restart strategies, thus assuming a stepwise change in parameters. To clarify the applicability of the PIE framework in such non-stationary settings, we distinguish between scenarios that can already be addressed within the current methodology and those that require further methodological development.

- **Drug affects AP shape and restitution properties:** If a drug primarily affects AP shape and restitution properties, these effects can be incorporated within the existing PIE framework. The effect upon AP shape and restitution can be pre-computed during calibration, and the PIE model would then correctly predict activation and repolarization patterns and associated ECGs under these modified conditions for a limit cycle. Accounting also for the transient accommodation after drug administration, a step-wise approach with repetitive re-calibration is required. However, as calibration of the PIE solver is very lightweight, the current framework readily accommodates such scenarios.
- **Stochastic physiological effects:** Effects such as drug-induced ectopy or transient instabilities cannot be accounted for with the current PIE formulation and would therefore require further development. Possible strategies include (i) solving the ionic model on-the-fly with the PIE solver, which will significantly reduce the computational advantage, resulting in a performance comparable to a reaction-eikonal model [2], or (ii) employing surrogate reduced-order models to approximate on-the-fly the solution of the ionic model. This may include

gaussian process emulation or machine learning methods. The latter approach is particularly promising and is currently the focus of ongoing and future investigations [3].

Acknowledging the importance of these limitations of the PIE solver, we have explicitly added a discussion on these aspects and their implications in the limitations section of the manuscript (p.26–27, l.1151–1207).

5. The model RD model is deterministic but should be chaotic particularly when it comes to modeling tachycardia/re-entry, will the PIE model also going to capture this chaotic behavior?

The Reviewer correctly points out that solutions of RD models are highly sensitive to perturbations in initial conditions and parameters, and therefore exhibit chaotic, yet deterministic, behavior. As the PIE solver approximates emerging properties observed in the RD model such as restitution behavior, the PIE model is likewise sensitive to perturbations.

To investigate differences in the response to parameter perturbations between the RD and PIE model, we repeated the highly sensitive functional reentry simulations of Experiment (E). We perturbed ionic model parameters in the RD model, recalibrated the PIE model to the perturbed RD model and observed the spatio-temporal evolution of the rotor under perturbed conditions. We show the results of this perturbation experiment in the Supplementary Sec. C.1.

As shown in the Supplementary Fig. C2, the overall wavefront patterns differ between perturbed and reference conditions, but behavior is very similar between RD and PIE model, demonstrating the PIE models ability to reproduce trends qualitatively comparable to those observed in the RD model. Over time more pronounced discrepancies emerge due to the accumulation of approximation errors.

As such, a perfectly matching response between RD and PIE to parameter perturbations over prolonged observation intervals cannot be achieved. However, it is important to note that this is also not the case for different implementations of the same RD model. Any difference in discretization scheme, numerical settings such as the choice of space and time step or solver tolerances, or even minor differences in round-off errors (difference in CPUs or number of threads/cores) will cause discrepancies over time between two otherwise exactly identical RD models. As shown recently, owing to the high sensitivity in arrhythmic regimes, arrhythmia simulations of e.g. ventricular tachycardia [4] or atrial fibrillation [5] are always afflicted with such stochastic uncertainties.

As a consequence, drawing conclusions from individual simulations, be it RD or PIE model, is not warranted. In view of using models for predicting EP behaviors in industrial or even clinical applications, more consistent strategies are needed that adopt a statistical rather than a purely deterministic approach. Addressing stochastic effects requires many-query simulations in which parameters are perturbed to quantify sensitivity and estimate uncertainty, enabling conclusions to be drawn from ensemble behavior rather than from single simulations. For such many-query scenarios the PIE solver is particularly suitable owing to its superior

computational efficiency. We emphasize this consideration now more explicitly in the Discussion (p.24–25, l.1077–1112).

6. Will the model work with different diseased condition? For example, under the Ca overload, LQTS syndrome type 1 (IKs loss of function) or CPVT (RyR alteration).

We thank the Reviewer for their interesting question. In line with response (4), the PIE model can replicate disease scenarios that are reflected in anatomical or structural changes or by variations in EP parameters. As long as the behavior is deterministic, the PIE solver is able to reproduce such behavior. This includes disease conditions associated to gain/loss of function of ion channels, or effects of Calcium cycling upon the action potential, by accounting for these effects at the calibration stage.

However, stochastic effects leading to spontaneous triggered activity due to EADs or DADs cannot be reproduced by the PIE model in its current implementation. Since both Long QT Syndrome Type 1 (LQTS1) and Catecholaminergic Polymorphic Ventricular Tachycardia (CPVT) involve such stochastic or transient events, the PIE solver in its current form is not suitable.

Extending the capabilities of the PIE solver to accommodate such important disease scenarios is feasible. A more complex state machine is required to phenomenologically encode the stochastic behavior, combined with additional spatial correlation measurements to determine whether local trigger events synchronize across a tissue area that is larger than the liminal length to actually elicit an ectopic beat. While feasible, accommodating such scenarios in a PIE solver will increase solver complexity and increase computational costs, and require significant further research efforts.

7. Could PIE model be used for studies of channelopathies? For example, different LQTS1 mutations lead to distinct functional effects on IKs (encoded by KCNQ1), and clinically leading to Torsades de Pointes.

We thank the reviewer for the suggestion of extending the applicability of the PIE solver to channelopathies. Our current implementation of the PIE solver is not a universal replacement for a RD model and as such cannot accommodate the full bandwidth of emergent phenomena associated with channelopathies. However, the PIE solver can, in principle, be extended to capture most of the key emergent properties associated with channelopathies.

All the points suggested by the Reviewer have been considered. We elucidate current limitations and potential extension in the revised manuscript in the Discussion to provide a perspective on the further generalization of the PIE concept (see p.26, l.1151). A more detailed rationale is provided below in our response.

In line with our response (4), the PIE model is capable of reproducing effects of channelopathies that are deterministic, and fully governed by phenomena encoded in the finite state machine. However, limitations exist with regard to representing stochastic events such as triggered activity due to EADs or DADs, or other stochastic/transient instabilities, which are critical for predicting events like Torsades de

Pointes or arrhythmic episodes in LQTS1 or CPVT.
Accounting for spontaneous induction by triggered activity would require a more sophisticated state machine and a more expansive lookup of precomputed cellular dynamics. While such an extension is, in principle, feasible, further research is required to generalize the PIE concepts to these scenarios.

7.a Because each LQTS1 mutation produces a different degree of IKs loss-of-functon(PMID 21451124), my understanding is that PIE would currently require separate re-validation for each mutation, which may diminish the computational advantage.

The reviewer is correct, any type of mutation would require a separate recalibration. This would also be the case for the RD model where channel properties are adjusted to fit observations made for different mutations. However, this would not diminish the computational advantage of the PIE model. Unlike with other surrogate models such as e.g. neural networks, no extensive training is needed. Physical properties are measured on single cell or strand models within seconds, at negligible cost. We included such consideration in the Discussion section, p.26, l.1169.

7.b Would it be feasible to incorporate ion-channel kinetic parameters directly into PIE model? For instance, prior studies (PMID: 37705243) have mapped IKs-related parameters to APD using simple logistic models that relate channel conductance and kinetic properties to early afterdepolarization risk. It might also be possible to build models to map to CV restitution. If such parameter–phenotype mappings could be embedded into the PIE/FSM framework, the approach might generalize across families of LQTS1 mutations (or other channelopathies), enabling accelerated whole-heart prediction across a spectrum of channel dysfunction.

We thank the reviewer for the suggestion and the useful references, which we are referring to now in the revised manuscript. Indeed, the PIE solver makes use of parameter-phenotype mappings such as AP(DI,t), APD(DI), CV(DI), CV(curvature), etc, but in the current implementation, these are limited. Certainly, this can be extended to multiparametric maps, such as e.g. AP(DI,Gkr,Gks,...,t) or APD(DI,Gkr,Gks, ...). Such extensions are readily incorporated in a PIE framework, at the cost of increasing the complexity of the finite state machine and a more expensive calibration. However, even for very high-dimensional maps, calibration will be relatively cheap as no organ-scale training data are required.

Most challenging is the extension of the PIE concept to account for stochastic phenomena such as triggered activity leading to PVCs or arrhythmias such as TdP, while preserving computational efficiency. This may be achievable by using reduced-order methods that are able to represent a broad spectrum of cellular dynamics at cheap computational costs. Potential solutions include:

- **Multiparametric maps**: As suggested by the Reviewer, the FSM of the PIE solver can be informed with multiparametric maps that are pre-computed at the calibration stage, including probabilistic maps such as p(EAD), as used in (PMID: 37705243). While such an approach is readily incorporated in a PIE

framework, it increases parameter dimensionality to be precomputed, stored and evaluated, entailing an increase in code complexity and compute costs.

- **Surrogate models**: Emulators such neural network surrogate models can be used to compute transmembrane voltages on-the-fly for large parameter spaces as in [3]. Such an approach maintains tractable storage and moderate computational costs. However, it requires an expensive training phase and may be limited in its ability to generalize to unseen parameter combinations. Moreover, while the interrogation of a network may be cheaper than solving a complex set of stiff ODEs, this will add non-negligible costs compared to the efficient lookups used in this study.

For both approaches of evaluating cellular dynamics and related emergent properties, additional spatial-coherence metrics must be measured, as triggered activity on the organ scale is governed by the liminal length. That is, cells within a sufficiently large area must undergo a spontaneous depolarizing event with sufficient temporal synchronization to be able to overcome the repolarizing force of adjacent subthreshold tissue, as we have investigated previously for DADs under SR Calcium overload conditions (PMID: 25969391). Acknowledging the importance of this discussion, we have explicitly added a discussion at p.26–27, l.1169–1207.

Reviewer 2

This work introduces a Physics-Informed Eikonal (PIE) model implemented into a computational framework designed to reproduce key electrophysiological behaviors, including activation, repolarization, conduction velocity restitution, curvature effects and diffusion-mediated repolarization smoothing, as well as to simulate arrhythmias and clinical ECGs in near-real-time. This is promising and a great contribution to computational cardiology with potential for clinical application, addressing a critical bottleneck in cardiac digital twins: the current issue with high computational costs associated with full RD simulations. I believe this manuscript is suitable for publication in Nature Communications; however, I suggest a few improvements below before it being accepted for publication (Minor Revisions).

We thank the reviewer for their positive assessment and constructive comments.

Major Concerns:

1. Validation of reentry should use quantitative metrics beyond visual comparisons: The functional reentry experiment currently uses qualitative assessment and phase singularity tracking. Given the nonlinear sensitivities observed, perfect overlap is impossible, but this piece of validation would benefit from using some quantitative metrics. These could include a comparison of dominant frequency maps, trajectory metrics such as mean squared error and phase-field correlation over time. This would help assess whether the model could be used for mechanistic simulations. If quantitative metrics cannot be used, please provide a justification on how qualitative assessment is sufficient.

We thank the Reviewer for their useful suggestions. We have incorporated all additionally requested metrics, including dominant frequency maps, trajectory error and phase-field correlation over time. We opted to use the more intuitive euclidean distance metric over the suggested MSE to quantify the error in phase singularity trajectories. We provide these quantitative metrics now in the revised manuscript. Specifically, we summarize results now in the new Figure 6, and state in p.13–14 l.593–637:

"The qualitative behavior of the PIE and R-D model (Fig. 5B, left) was comparable, with wave fronts and wave backs remaining spatio-temporally correlated as they traversed the domain. The filament tracked at the surface followed comparable trajectories, covering a similar area and exhibiting a morphology consistent with previous reports [6]. The frequency analysis shown in Fig. 6A indicates that the PIE and RD models exhibit closely correlated dominant frequencies. However, the PIE model does not fully capture all frequency components of the R-D reentrant activity, resulting in more irregular dynamics compared to the R-D model. Frequency-related differences in reentrant behavior are also evident in the dominant frequency maps, where regions of elevated frequency are similarly localized in both PIE and R-D simulations, yet differ markedly in their spatial distribution and amplitude.
The rotor in the PIE model appears less stable than in the R-D model (Fig. 5, right), consistent with the temporal evolution of the phase singularity location error between the two models (Fig. 6B), averaging around 10 mm *and increasing over time. The phase evolution of the reentrant activity (Fig. 6C) is qualitatively similar between the models. However, the PIE solution exhibits slightly reduced smoothness*

in phase definition near the wavefront and waveback. These differences are further reflected in the temporal evolution of phase correlation, which quantifies divergence between the two solutions over time. Phase map correlation analysis indicates that agreement is lowest in the vicinity of the rotor core, while remaining comparable in regions distal to the core."

2. DTW and PCC are useful metrics to validate PIE results against ECG, but these may be insufficient. The ECG comparison presented in Table 1 shows good results overall, but some leads (e.g., V4–V6, mean PCC 0.71) show moderate discrepancies. No clinical threshold is provided to indicate what level of mismatch is acceptable for clinical tasks including guidance for ablation procedures. Additional metrics such as QRST integral differences would be beneficial for this piece of validation.

We thank the reviewer for the insightful comments regarding the quantitative assessment of ECGs. We agree that additional quantitative metrics can further substantiate the accuracy of the PIE solver with respect to ECG predictions.
In response, we implemented a methodological improvement that led to a substantial enhancement in ECG agreement (see revised Fig. 7, Secs. (F), p.17–18, l.765–822, and the Discussion section, p.22, l.968–994). A detailed analysis of the discrepancies in the previous submission revealed that changes in VT path lengths – caused by global mesh coarsening that did not respect domain boundaries – were the primary source of error.
We further included an additional quantitative validation of the updated ECG predictions based on R–R interval measurements, which are indicative of the VT cycle length (see revised Tab. 1). The ECGs generated by the PIE solver are now in excellent morphological agreement with those obtained from the reference RD simulations. The remaining differences are minimal and lie well within the range of both residual variability in measured ECGs and simulation-related uncertainties.
In light of the minimal differences observed in R–R intervals, we did not pursue QRST integral analysis. Instead, we provide a more detailed examination of the influence of mesh resolution on ECG accuracy (see new Supplementary Sec. C.2).

Regarding ECG mismatch, clinical use of morphology is largely confined to specific settings, such as pace mapping for localization near VT exit sites, often supported by proprietary algorithms (e.g. Biosense Webster's PaSo module within the CARTO system). To our knowledge, no standardized thresholds defining acceptable ECG mismatch exist. Based on our experience with pace mapping data, the level of agreement observed between PIE and RD ECGs would not be attainable in routine clinical practice.
For computational guidance of ablation, studies based on RD models typically do not report ECGs as these are often poorly correlated with patient recordings [7, 8]. Contributing factors include coarse spatial resolution, limited model calibration, omission of key anatomical structures (e.g. the conduction system) and the absence of torso models required for quantitatively accurate ECG prediction. Moreover, RD-derived ECGs are highly sensitive to mesh resolution, parameter choices and numerical implementation, leading to variability that exceeds the differences observed here between RD and PIE predictions [4].

From a clinical perspective, PIE-derived ECGs may more faithfully capture patient-specific electrophysiology as the lightweight formulation enables rapid calibration and exhibits reduced sensitivity to spatial resolution compared with RD models. Consistently, a reaction–eikonal approach achieved near-perfect agreement with patient ECGs [9].

3. The manuscript repeatedly claims the PIE model provides "full physical fidelity". This is an overstatement, since it still has some approximations. For instance: 1) Ionic currents are not resolved; 2) Diffusion is only locally approximated. These claims should then be lessened. The proposed PIE model offers high fidelity, but not full mechanistic fidelity.

We agree that describing the PIE model as offering "full physical fidelity" may overstate its capabilities. While PIE represents a powerful and novel approach with broad potential applications, we have revised the manuscript to temper this claim. We now describe PIE as providing "high physical fidelity" relative to RD models and have added a detailed discussion of its limitations, including physical phenomena captured by RD models but not represented in the PIE model (see p.24–27 l.1067–1207).

4. There are several questions around model scalability and application in real-time. While speedups of up to 400x are very worthy, the practical real-time usage of the framework depends on a few computational aspects, including mesh resolution constraints, solver time refinement, and parallelization efficiency across CPU/GPU hardware. - What is the minimal resolution to reproduce clinical ECGs in a reliable manner?

We thank the reviewer for their thoughtful assessment of the PIE model's practical performance.
We expanded our assessment of scalability depending on mesh size now with an evaluation of the impact of mesh resolution on model fidelity and computational demands by benchmarking the whole-heart experiment (F). We performed a systematic analysis of mesh resolution on ECG fidelity. As noted in the whole-heart VT experiment (F), ECG fidelity depends not only on resolution but also on the preservation of domain interfaces. For VT, this primarily concerns maintaining scar and border zone topology, which defines the VT circuit path length. We evaluated two coarsening strategies applied to the original 250 μm RD mesh: (i) global coarsening without preserving scar topology, and (ii) domain-boundary-preserving coarsening that maintains scar and border zone integrity. In both cases, average mesh spacing was increased to 1000 μm, 2000 μm, and 3000 μm (see Supplementary Sec. C.2).
With boundary-preserving mesh coarsening, ECG morphology is retained, showing high PCC and DTW agreement while substantially reducing computational cost. Simulations on the 3000 μm mesh ran 6.5× faster than on the 1000 μm mesh (34 s vs. 221 s). In contrast, global coarsening degraded ECG fidelity at resolutions coarser than 1000 μm and, for coarse meshes, altered VT sustenance.
The revised manuscript now reports whole-heart performance metrics (p. 20, l.821—916), the effect of mesh resolution on ECG fidelity (Supplementary Sec. C.2), and

computational cost (see Supplementary Sec. C.3). All new meshes and lead-field data are available via the online resources.

4.a How does PIE performance scale on GPUs, which are now standard for clinical modeling?
Regarding a GPU/CPU performance comparison: for compatibility and debugging reasons, the current development of the PIE model has focused on a CPU-based implementation without GPU support. Consequently, we are not yet able to provide a quantitative assessment of the model's scalability on GPUs. However, we note that wavefront propagation and diffusion, which constitute the most computationally expensive components of the PIE solver, are well suited to benefit from the massive parallelism offered by GPUs [10]. However, a GPU implementation requires a substantial effort, which we are unable to invest in this study, which is focused on introducing the conceptual framework and evaluating the potential. Fully optimizing performance by exploiting hardware acceleration is beyond the scope of this study, but we will certainly consider developing a GPU-based variant of the PIE model in the future.

5. The authors acknowledge limitations but should include explicit text about scenarios where PIE should not be used, e.g., early after-depolarizations or drug-induced ionic changes.

We thank the reviewer for highlighting this omission and have revised the manuscript accordingly. We now explicitly note that the PIE model is not a universal replacement for RD models. The discussion of PIE's limitations has been expanded (p.25–26, l.1151–1207), including scenarios where it is not applicable, such as stochastic ectopy driven by triggered activity and transient non-stationary phenomena like drug-induced ionic changes. We also complement this discussion with a perspective on potential extensions of the PIE model to accommodate a broader range of phenomena, including stochastic and non-stationary phenomena.

Minor Concerns:

Remarks on code availability:

The code appears to be reasonably well-organized, though the level of reproducibility varies across different components. The repository does include a README file, and instructions are clear on the GitHub page. I was able to install the required packages and reproduce the experimental results.

We are glad the reviewer could reproduce the results and have clarified the instructions accordingly.

References

[1] Kalb, S.S., Dobrovolny, H.M., Tolkacheva, E.G., Idriss, S.F., Krassowska, W., Gauthier, D.J.: The restitution portrait: A new method for investigating rate-dependent restitution. J Cardiovasc Electrophysiol. **15**, 698–709 (2004) https://doi.org/10.1046/j.1540-8167.2004.03550.x

[2] Neic, A., Campos, F.O., Prassl, A.J., Niederer, S.A., Bishop, M.J., Vigmond, E.J., Plank, G.: Efficient computation of electrograms and ECGs in human whole heart simulations using a reaction-eikonal model. J. Comput. Phys. **346**, 191–211 (2017) https://doi.org/10.1016/j.jcp.2017.06.020

[3] Grandits, T., Augustin, C.M., Haase, G., Jost, N., Mirams, G.R., Niederer, S.A., Plank, G., Varro, A., Virág, L., Jung, A.: Neural network emulation of the human ventricular cardiomyocyte action potential: A promising tool for more efficient computations in quantitative systems pharmacology studies. J. Pharmacol. Toxicol. Methods **135**, 107850 (2025) https://doi.org/10.7554/eLife.91911.2

[4] Bishop, M.J., Plank, G.: Stochastic virtual heart model predictions. Nat Cardiovasc Res **4**, 539–542 (2025) https://doi.org/10.1038/s44161-025-00641-1

[5] Deng, D., Murphy, M.J., Hakim, J.B., Franceschi, W.H., Zahid, S., Pashakhanloo, F., Trayanova, N.A., Boyle, P.M.: Sensitivity of reentrant driver localization to electrophysiological parameter variability in image-based computational models of persistent atrial fibrillation sustained by a fibrotic substrate. Chaos **27**(9) (2017) https://doi.org/10.1063/1.5003340

[6] Jacquemet, V.: An eikonal approach for the initiation of reentrant cardiac propagation in reaction–diffusion models. IEEE Trans. Biomed. Eng. **57**(9), 2090–2098 (2010) https://doi.org/10.1109/TBME.2010.2051156

[7] Arevalo, H.J., Vadakkumpadan, F., Guallar, E., Jebb, A., Malamas, P., Wu, K.C., Trayanova, N.A.: Arrhythmia risk stratification of patients after myocardial infarction using personalized heart models. Nat. Commun. **7**, 11437 (2016) https://doi.org/10.1038/ncomms11437

[8] Prakosa, A., Arevalo, H.J., Deng, D., Boyle, P.M., Nikolov, P.P., Ashikaga, H., Blauer, J.J.E., Ghafoori, E., Park, C.J., Blake, R.C., Han, F.T., MacLeod, R.S., Halperin, .R., Callans, D.J., Ranjan, R., Chrispin, J., Nazarian, S., Trayanova, N.A.: Personalized virtual-heart technology for guiding the ablation of infarct-related ventricular tachycardia. Nat. Biomed. Eng. **2**, 732–740 (2018) https://doi.org/10.1038/s41551-018-0282-2

[9] Grandits, T., Gillette, K., Plank, G., Pezzuto, S.: Accurate and efficient cardiac digital twin from surface ECGs: Insights into identifiability of ventricular conduction system. Med. Image Anal. **105**, 103641 (2025) https://doi.org/10.1016/j.media.2025.103641

[10] Fu, Z., Kirby, R.M., Whitaker, R.T.: A fast iterative method for solving the eikonal equation on tetrahedral domains. SIAM J Sci Comput. **35**, 473–494 (2013) https://doi.org/10.1137/120881956

Response to the Referee Reports for: A Physics-Informed Eikonal Model for Simulating Arrhythmias in the Human Heart in Real-Time

Thomas G. Schrotter[1,2], Matthias A. F. Gsell[1], Elena Zappon[1], Christoph M. Augustin[1,2,3], Aurel Neic[2], Simone Pezzuto[4,5], Gernot Plank[1,2,3,*,#]

[1]Gottfried Schatz Research Center, Division of Medical Physics and Biophysics, Medical University of Graz, Neue Stiftingtalstraße 6, 8010, Graz, Austria
[2]NumeriCor GmbH, Conrad-von-Hötzendorfstraße 26/2, 8010, Graz, Austria
[3]BioTechMed-Graz, Mozartgasse 12/II, 8010, Graz, Austria
[4]Department of Mathematics, University of Trento, Via Sommarive 14, 38123, Trento, Italy
[5]Euler Institute, Università della Svizzera italiana, Via Buffi 13, 6900, Lugano, Switzerland
[*]Corresponding author: gernot.plank@medunigraz.at

We, the authors, would like to thank the Reviewers for their time invested in reading our manuscript and for providing a helpful set of reviews. All of the suggestions and comments from the Reviewers have been thoroughly considered and, where appropriate and possible, we have changed the manuscript accordingly. In the sections below, we have responded individually to every concern and comment raised by the Reviewers.
For the sake of tracking changes implemented in our revision, both in the manuscript and the response, changes corresponding to concerns or comments of Reviewer 1 have been highlighted in red and changes of Reviewer 2 have been highlighted in blue. Changes independent of Reviewer comments have been highlighted in purple.

Reviewer's Responses

Reviewer 1

The authors have addressed nearly all of my comments clearly and thoroughly, and I am satisfied with the revisions overall.
We thank the reviewer for their positive assessment and constructive comments.

Remaining Concerns:

1.a I have one remaining point regarding the response to my major concern 1a. The inclusion of the outlier plots in Figure 4 significantly improves the validation. However, the errors observed for certain experiments, particularly for CV under diffusion-dominated conditions and LRT in these settings, appear relatively large. Can authors provide additional clarification on the reasons/causes for these discrepancies.

We thank the reviewer for raising their concerns and agree that more elaboration on the errors and outliers seen in Manuscript Fig. 4, in particular during the diffusion benchmark (C), is in order. We therefore complemented our results in the manuscript with more detail (see Manuscript p.12), supported by a dedicated supplementary section (see Supplementary Sec. C.1 - p.14–15) where we address the causes of the observed discrepancies. In general, these emerged from modeling differences between the RD and the PIE model and are summarized by the following insights:

- **CV errors**: Underestimations in CV, visualized by the blue regions in error maps, were observed at stimulation sites, wavefront collisions and sealed ends where the wavefront propagation terminates (see Manuscript Fig. 4, middle), reflected by outliers located in the right lower part of the outlier plots. At those sites, the RD model experienced a momentary acceleration of propagation caused by a diffusion-induced positive source-sink imbalance which the PIE model does not consider, except in presence of wavefront curvature, which causes the observed CV underestimations. In contrast, the observed overestimations, visualized by the red regions in error maps, emerged from misalignment of the wavefront formed by the drag effect between the planar wavefronts of healthy and border-zone tissue (see Supplementary Fig. C2A). In the PIE model, the wavefront angle appears less steep compared to the RD model, attributed again to the mentioned source-sink effect during diffusion, leading to an earlier activation along the propagation direction of the drag effect.
- **LRT errors**: The discrepancies in LRTs emerge predominantly from the simplified modeling assumptions of diffusion in the PIE model that causes a different distribution of APDs, in particular near the interface of healthy and border-zone tissue. In the PIE model, diffusion affects the APD linearly, while diffusion in RD models acts linearly on the transmembrane voltage, V_{m}, which causes nonlinear changes in APD. This results in an overemphasized diffusion effect on APD near the interface between both regions (see Supplementary Fig. C2B) and therefore a distortion of the LRTs when using the PIE model.

Reviewer 2

The authors have made substantial revisions to the manuscript and have satisfactorily addressed all of my comments and concerns. I have no further comments and recommend the manuscript for publication.
We thank the reviewer for their positive assessment of our revision.

Remarks on code availability:

The code is appropriate and reusable, with clear instructions.

We thank the reviewer for their positive assessment of the provided code.

Response to the Referee Reports for: A Physics-Informed Eikonal Model for Simulating Arrhythmias in the Human Heart in Real-Time

Thomas G. Schrotter[1,2], Matthias A. F. Gsell[1], Elena Zappon[1], Christoph M. Augustin[1,2,3], Aurel Neic[2], Simone Pezzuto[4,5], Gernot Plank[1,2,3,*,#]

[1]Gottfried Schatz Research Center, Division of Medical Physics and Biophysics, Medical University of Graz, Neue Stiftingtalstraße 6, 8010, Graz, Austria
[2]NumeriCor GmbH, Conrad-von-Hötzendorfstraße 26/2, 8010, Graz, Austria
[3]BioTechMed-Graz, Mozartgasse 12/II, 8010, Graz, Austria
[4]Department of Mathematics, University of Trento, Via Sommarive 14, 38123, Trento, Italy
[5]Euler Institute, Università della Svizzera italiana, Via Buffi 13, 6900, Lugano, Switzerland
[*]Corresponding author: gernot.plank@medunigraz.at

We, the authors, would like to thank the Reviewers for their time invested in reading our manuscript and for providing a helpful set of reviews. All of the suggestions and comments from the Reviewers have been thoroughly considered and, where appropriate and possible, we have changed the manuscript accordingly. In the sections below, we have responded individually to every concern and comment raised by the Reviewers.
For the sake of tracking changes implemented in our revision, both in the manuscript and the response, changes corresponding to concerns or comments of Reviewer 1 have been highlighted in red and changes of Reviewer 2 have been highlighted in blue. Changes independent of Reviewer comments have been highlighted in purple.

Reviewer's Responses

Reviewer 1

Authors has addressed the remaining question. I have no further questions or comments.
We thank the reviewer for their positive assessment of our revision.
